# Neutral sphingomyelinase mediates the co-morbidity trias of alcohol abuse, major depression and bone defects

**DOI:** 10.1038/s41380-021-01304-w

**Published:** 2021-09-28

**Authors:** Liubov S. Kalinichenko, Christiane Mühle, Tianye Jia, Felix Anderheiden, Maria Datz, Anna-Lisa Eberle, Volker Eulenburg, Jonas Granzow, Martin Hofer, Julia Hohenschild, Sabine E. Huber, Stefanie Kämpf, Georgios Kogias, Laura Lacatusu, Charlotte Lugmair, Stephen Mbu Taku, Doris Meixner, Nina Tesch, Marc Praetner, Cosima Rhein, Christina Sauer, Jessica Scholz, Franziska Ulrich, Florian Valenta, Esther Weigand, Markus Werner, Nicole Tay, Conor J. Mc Veigh, Jana Haase, An-Li Wang, Laila Abdel-Hafiz, Joseph P. Huston, Irena Smaga, Malgorzata Frankowska, Malgorzata Filip, Anbarasu Lourdusamy, Philipp Kirchner, Arif B. Ekici, Lena M. Marx, Neeraja Puliparambil Suresh, Renato Frischknecht, Anna Fejtova, Essa M. Saied, Christoph Arenz, Aline Bozec, Isabel Wank, Silke Kreitz, Andreas Hess, Tobias Bäuerle, Maria Dolores Ledesma, Daniel N. Mitroi, André M. Miranda, Tiago G. Oliveira, Erich Gulbins, Bernd Lenz, Gunter Schumann, Johannes Kornhuber, Christian P. Müller

**Affiliations:** 1 Department of Psychiatry and Psychotherapy, University Clinic, Friedrich-Alexander-University Erlangen-Nürnberg, Erlangen, Germany; 2 The Centre for Population Neuroscience and Stratified Medicine (PONS), ISTBI, Fudan University, Shanghai, China; 3 PONS Centre and SGDP Centre, Institute of Psychiatry, Psychology and Neuroscience, King’s College London, London, UK; 4 Department for Anesthesiology and Intensive Care, Faculty of Medicine, University of Leipzig, Leipzig, Germany; 5 Biomedical Center, Institute of Cardiovascular Physiology and Pathophysiology, Faculty of Medicine, Ludwig-Maximilians-Universität München, Munich, Germany; 6 Department of Psychosomatic Medicine and Psychotherapy, Friedrich-Alexander-University of Erlangen-Nürnberg, Erlangen, Germany; 7 School of Biomolecular and Biomedical Science, UCD Conway Institute, University College Dublin, Dublin, Ireland; 8 Center for Behavioral Neuroscience, Institute of Experimental Psychology, University of Düsseldorf, Düsseldorf, Germany; 9 Department of Drug Addiction Pharmacology, Maj Institute of Pharmacology, Polish Academy of Sciences, Kraków, Poland; 10 Division of Child Health, Obstetrics and Gynaecology, School of Medicine, University of Nottingham, Nottingham, UK; 11 Institute of Human Genetics, Friedrich-Alexander-University of Erlangen-Nuremberg (FAU), Erlangen, Germany; 12 Department of Biology, Animal Physiology, Friedrich-Alexander-University of Erlangen-Nürnberg, Erlangen, Germany; 13 Institute for Chemistry, Humboldt University, Berlin, Germany; 14 Department of Internal Medicine 3—Rheumatology and Immunology, Friedrich-Alexander-University of Erlangen- Nürnberg, Erlangen, Germany; 15 Deutsches Zentrum für Immuntherapie (DZI), Friedrich-Alexander-University of Erlangen-Nuremberg (FAU), Erlangen, Germany; 16 Department of Experimental and Clinical Pharmacology and Toxicology, Emil Fischer Center, Friedrich-Alexander-University of Erlangen-Nuremberg (FAU), Erlangen, Germany; 17 Preclinical Imaging Platform Erlangen, Institute of Radiology, University Hospital Erlangen, Erlangen, Germany; 18 Centro Biologia Molecular Severo Ochoa (CSIC-UAM), Madrid, Spain; 19 Life and Health Sciences Research Institute (ICVS), School of Medicine, Campus Gualtar, University of Minho, Braga, Portugal; 20 ICVS/3B’s—PT Government Associate Laboratory, Braga/Guimarães, Portugal; 21 Department of Molecular Biology, University of Duisburg-Essen, Essen, Germany; 22 Department of Surgery, College of Medicine, University of Cincinnati, Cincinnati, OH USA; 23 Department of Addictive Behavior and Addiction Medicine, Central Institute of Mental Health (CIMH), Medical Faculty Mannheim, Heidelberg University, Mannheim, Germany; 24 PONS Centre, Charite Mental Health, Dept. of Psychiatry and Psychotherapie, CCM, Charite Universitaetsmedizin Berlin, Berlin, Germany; 25 Centre for Drug Research, Universiti Sains Malaysia, Minden, Penang Malaysia

**Keywords:** Neuroscience, Addiction

## Abstract

Mental disorders are highly comorbid and occur together with physical diseases, which are often considered to arise from separate pathogenic pathways. We observed in alcohol-dependent patients increased serum activity of neutral sphingomyelinase. A genetic association analysis in 456,693 volunteers found associations of haplotypes of *SMPD3* coding for NSM-2 (NSM) with alcohol consumption, but also with affective state, and bone mineralisation. Functional analysis in mice showed that NSM controls alcohol consumption, affective behaviour, and their interaction by regulating hippocampal volume, cortical connectivity, and monoaminergic responses. Furthermore, NSM controlled bone–brain communication by enhancing osteocalcin signalling, which can independently supress alcohol consumption and reduce depressive behaviour. Altogether, we identified a single gene source for multiple pathways originating in the brain and bone, which interlink disorders of a mental–physical co-morbidity trias of alcohol abuse—depression/anxiety—bone disorder. Targeting NSM and osteocalcin signalling may, thus, provide a new systems approach in the treatment of a mental–physical co-morbidity trias.

## Introduction

Mental disorders are considered to be brain disorders arising from biological malfunctions in the central nervous system. Currently available pharmacotherapies focus solely on re-establishing brain homoeostasis. However, in clinical reality virtually all mental disorders show a high co-morbidity with peripheral organ dysfunctions. Thereby, peripheral dysfunctions interact with mental disorders and complicate disease progression and compromise treatment success [1].

Alcohol abuse is a wide spread phenomenon that may result in addiction. Alcohol abuse frequently co-occurs with affective disorders, such as major depression and anxiety disorders [2]. It is also often accompanied by physical diseases, such as osteoporosis [3]. In fact, all three disorders are highly comorbid and may represent a co-morbidity symptom trias with distinct mental and physical dimensions [4].

Sphingolipids are major components of cellular membranes in all organ systems, which shape functional structure and its highly dynamic plasticity [5–8]. In this paper, we test the hypotheses that naturally occurring variations in the activity of the enzyme neutral sphingomyelinase-2 (NSM) may be a common origin of the clinically highly relevant co-morbidity trias of alcohol abuse, major depression and bone defects. We hypothesised that respective traits may be associated with shared mutations in the NSM coding gene *Smpd3*, and confirmed this in humans. We used genetic and pharmacological animal models of reduced NSM activity to investigate how this would give rise to single symptoms of either alcohol abuse, emotional dysfunction, or bone mineralisation defects.

## Results

### NSM activity is enhanced in patients with alcohol use disorder

**Fig. 1:**
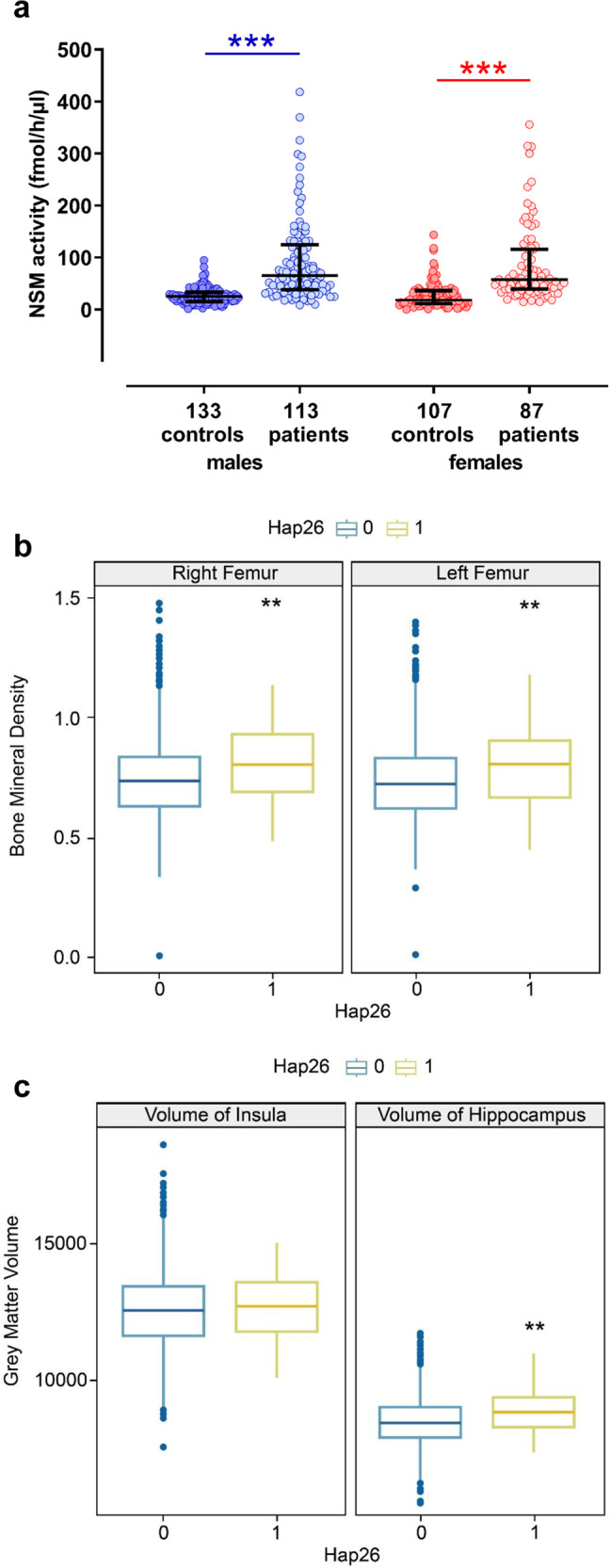
Association studies of neutral sphingomyelinase-2 (NSM) in humans. **a** NSM activity in serum is increased in patients with alcohol use disorder in early abstinence admitted for detoxification treatment compared to age-matched healthy controls (numbers for group size indicated at *x*-axis, ***P < 0.001). **b** A genetic haplotype association study in healthy humans showed significant associations of the NSM encoding *SMPD3* gene haplotype 26 (Hap26) with bone mineral density of the femur and (**c**) hippocampal, but not insula volume (*n* = 4515; 52.9% female; *P < 0.05; **P < 0.01 vs. non-Hap26 carriers).

Searching for blood markers of alcohol addiction in humans, we measured serum NSM activity of early-abstinent male and female patients diagnosed with alcohol use disorder upon hospital admission for detoxification treatment. In both, males and females, we observed a significantly enhanced NSM activity compared to healthy controls (Fig. 1a). This observation suggests an involvement of NSM in alcohol addiction.

### Association of *SMPD3* haplotypes in humans

Based on NSM activity as a biomarker signal for alcoholism, we tested for the associations of single nucleotide polymorphisms in the NSM-2 coding gene *SMPD3* with alcohol use in a population of 456,693 participants (56% female) with complete genotype and behavioural data from the UK Biobank, a large cohort of United Kingdom residents aged 40–69 years [9] (Table S1). Phased haplotypes were obtained using the SHAPEIT3 software [10] and extracted for the region covering the *SMPD3* gene (GRCh37, 16: 68,387,230–68,487,409). Haplotype phases with minor haplotype frequency (MHF) <0.01 were excluded, resulting in 26 haplotypes available for analysis (Table S2). We found 18 haplotypes that showed significant associations with at least one behavioural measure, with the alcohol variables having the most significant univariate associations. Our results suggest that *SMPD3* is associated with behavioural outcomes including alcohol consumption, as well as with anxiety and depressive symptoms (Table S3). Based on the previously identified role of osteoblast *SMPD3* expression in bone mineralisation [11–13], we determined the relationship between *SMPD3* and bone density. We conducted a univariate analysis between each haplotype phase and total bone mineral density (BMD) of the left and right femurs. We found significant associations between haplotype Hap26 and both the left and right femur BMD (Fig. 1b, Table S4).

Our findings in mice with reduced NSM function (see below) suggested a selectively altered development of the dorsal hippocampus (DH) and insular cortex (IC). Thus, we investigated this relationship in human participants. Using the same haplotype that had a significant association with bone density, Hap26, we conducted a Hotelling’s *t* test with grey matter volume of the hippocampus and IC. We found a significant association between Hap26 and grey matter volume and with DH, but not with IC volume (Fig. 1c). These findings suggest a strong link between the natural variance in the *SMPD3* gene with alcohol abuse, emotional behaviour and bone density. Part of this association may be explained by the developmental effects of NSM on DH maturation in humans.

### Blocking NSM reduces alcohol consumption

**Fig. 2:**
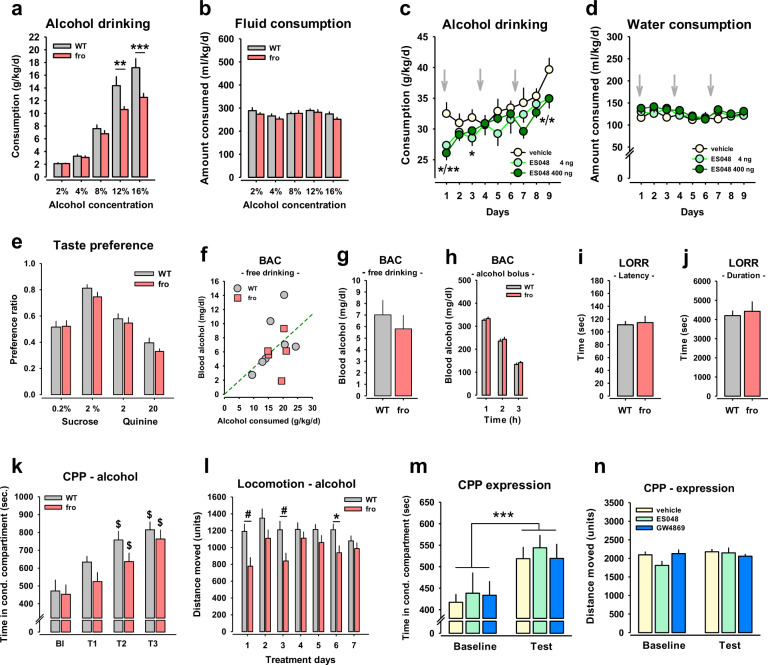
Neutral sphingomyelinase-2 (NSM) controls alcohol consumption, but not sedating and conditioned reinforcing effects in mice. **a** Mice with a heterozygous NSM knock out (fro) show reduced alcohol consumption in a two-bottle free-choice paradigm compared to wild type (WT) controls. **b** Lack of NSM function did not affect total fluid consumption in mice. Data are expressed as means ± s.e.m. (*n* = 8 per group; **P < 0.001; ****P < 0.001 vs. WT). **c** Alcohol drinking was reduced in C57Bl6J mice in a two-bottle free-choice drinking test by repeated treatment with the NSM inhibitor ES048 (i.p., grey arrows). **d** No effect of the ES048 treatment was observed on water consumption in these animals. Data are expressed as means ± s.e.m. (*n* = 10 per group; *P < 0.05; **P < 0.01 vs. vehicle control). **e** No role of NSM in taste preference. **f**, **g** Free-choice alcohol drinking yielded a proportional blood alcohol concentration (BAC) in heterozygous NSM deficient mice (fro) and wild type (WT) mice at individual level (*n* = 7 per group). **h** No difference in BAC after bolus injection of alcohol (3g/kg, i.p) in fro and WT mice (*n* = 9–10 per group). **i**, **j** Lack of NSM function has no effect on the sedating properties in the loss of the rightening reflex (LORR) of a single alcohol injection (3.5 g/kg; i.p.) as shown in latency and duration of sedating effects (*n* = 9 per group). **k** NSM has no role in the establishment of the conditioned reinforcing effects of alcohol in mice in a conditioned place preference (CPP) test, but (**l**) is required for locomotor activating effects of alcohol (2 g/kg, i.p.; Bl baseline, T test trial). Data are expressed as means ± s.e.m. (*n* = 11–15 per group; ^$^*P* < 0.05, vs. BL). **m** NSM is not involved in the expression of the conditioned reinforcing and conditioned locomotor effects of alcohol in mice as the two NSM inhibitors GW4869 and ES048 applied before CPP retrieval, did not affect CPP expression or (**n**) conditioned locomotor effects. Data are expressed as means ± s.e.m. (*n* = 9–10 per group; ***P < 0.01, ANOVA, effect of test trial).

A key behaviour of alcohol addiction is the consumption of easily available alcohol. We confirmed a role of NSM in voluntary alcohol consumption in a mouse model. In order to evaluate the effects of a reduced, but not absent NSM function, we tested *heterozygous* NSM-2 knock out mice (fro) [11] in a two-bottle free-choice alcohol consumption test [14, 15]. Thereby, fro mice showed a reduced consumption and preference of alcohol compared to wild type (WT) controls (Figs. 2a, S1a). There was no effect of NSM on total fluid intake (Fig. 2b). Reduced NSM activity had no effect on preference of sweet tasting solution or avoidance of bitter tasting solution in the mice (Fig. 2e). Neither did NSM affect alcohol bioavailability after drinking (Fig. 2f) nor after a bolus injection (Fig. 2g, h). We did not find a role of NSM in the sedating effects of alcohol as measured by the loss of the righting reflex latency (Fig. 2i) and duration (Fig. 2j).

In order to test whether the NSM role is an acute effect or depends on developmental function, as it would be the case in a natural mutation of the *Smpd3* gene, we tested the effects of an acute pharmacological inhibition of NSM activity on alcohol drinking. For this purpose, we determined an effective dose of the new NSM antagonist ES048 [16] in brain tissue (Fig. S2). In a separate test, ES048 attenuated previously established voluntary alcohol consumption in an intermittent access 20 vol.% alcohol-drinking test (Figs. 2c, S3) without changing overall fluid consumption (Fig. 2d). These findings suggest that attenuated NSM activity reduces voluntary alcohol consumption.

### No NSM control of the conditioned reinforcing effects of alcohol

A major driver of alcohol addiction is drug-seeking behaviour. This is based on the establishment and retrieval of the conditioned reinforcing effects of alcohol. We tested this in the establishment of an alcohol-induced conditioned place preference (CPP) in fro mice [17]. We found only a marginal effect of NSM activity reduction in the establishment of an alcohol CPP (Fig. 2k). However, NSM was required for the locomotor stimulant effects of alcohol (2 g/kg; i.p.) (Figs. 2l, S4–6). The expression of a previously established alcohol CPP was not affected by NSM antagonism with ES048 or GW4869 in mice (Fig. 2m). Neither did these treatments affect locomotor activity (Figs. 2n, S7–9). Altogether, these findings indicate that NSM is neither required for the establishment nor the retrieval of the conditioned reinforcing effects of alcohol, but is required for acute alcohol-induced locomotor stimulation.

### Reduced NSM activity has antidepressant and anxiolytic effects

**Fig. 3:**
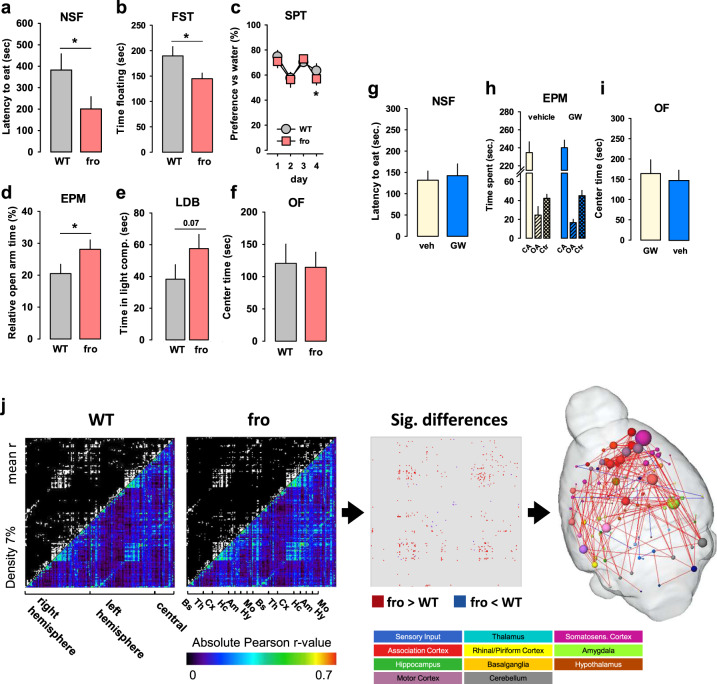
Neutral sphingomyelinase-2 (NSM) controls emotional behaviour. Mice with a heterozygous NSM-2 knock out (fro) show reduced depression-like and anxiety-like behaviour, but preserved hedonic tone compared to wild type (WT) controls. **a** Novelty suppressed feeding (NSF) test. **b** Forced swim test (FST). **c** Sucrose preference test (SPT). **d** Elevated plus maze (EPM). **e** Light–dark box test (LDB, *P* = 0.07). **f** Open field test (OF). Data are expressed as means ± s.e.m. (*n* = 8–11 per group; *P < 0.05 vs. WT). **g–i** Acute inhibition of NSM with GW4869 (GW, 2 mg/kg/day, i.p.) had no effect on emotional behaviour in naïve mice (*n* = 7 per group; OA open arm, CA closed arm, Ctr centre). **j** Differences in functional connectivity of fro and WT mice. By comparing symmetric mean functional connectivity matrices (left, lower triangle: mean positive correlation coefficients of all connections within the brain; upper triangle: binarized matrix containing only the 7% strongest connections) of female fro (*n* = 10) and WT mice (*n* = 10). Significant differences (middle) were calculated using network based statistics, *p* > 0.193). Significant differences are displayed as 3D-networks within a transparent brain surface (right) at the respective 3D positions of the centre of gravity of each brain structure. Colour-coded nodes represent one out of 206 single brain regions (orphan nodes are not shown). Edges between the nodes represent the significantly different connections (red: fro > WT; blue: fro < WT).

Our human genetic study suggested an association of natural variability in *SMPD3* and emotionality. We tested in mice how NSM would affect the depression- and anxiety-like phenotype of mice [18, 19]. We found a significant reduction of depression-like behaviour in fro mice in the novelty supressed feeding (NSF; Fig. 3a) test and in the forced swim test (FST; Fig. 3b), but no major change of hedonic tone in the sucrose preference test (SPT) (Fig. 3c). Anxiety-like behaviour was significantly reduced in the elevated plus maze (EPM; Fig. 3d) test and, as a trend, in the light–dark box test (LDB; Fig. 3e), but not in the open field (OF) (Fig. 3f). A reduction of NSM activity had little effects on general locomotor activity of mice in all tests (Figs. S10–12). No effects on depression-like or anxiety-like behaviours were observed when NSM activity was pharmacologically inhibited with the NSM antagonist GW4869 in mice in the NSF (Fig. 3g), the EPM (Fig. 3h), or the OF test (Figs. 3i, S13). These data suggest that reduced NSM activity attenuates depression-like and anxiety-like behaviour in adults as a developmentally mediated effect.

### Reduced NSM activity boosts connectivity in the neocortex

In order to identify a neuronal mechanism for the beneficial effects of a developmental NSM attenuation, a resting state fMRI analysis of the adult brain connectome was performed. It showed largely enhanced functional connectivity of the somatosensory-, motor-, and association cortices and the amygdala in the NSM deficient compared to WT mice (Fig. 3j). This may suggest that the impact of NSM on depression/ anxiety during development might be mediated by an enhanced connectivity between cortical regions and emotion processing amygdala.

### NSM control of bone mineralisation and osteocalcin signalling

Based on the human association of *SMPD3* with bone mineralisation, we tested how reduced NSM activity would affect bone density using bone-computed tomography (CT) in a mouse model. An NSM deficit did not change bone density of the scalp, spinal cord, femur, tibia, humerus, and radius/ulna (Fig. S14). Chronic free-choice alcohol consumption had no effect on bone density, and no interactions of NSM activity and alcohol effects were observed. These findings suggest that a heterozygous NSM depletion is not sufficient to induce obvious bone deficits in mice.

**Fig. 4:**
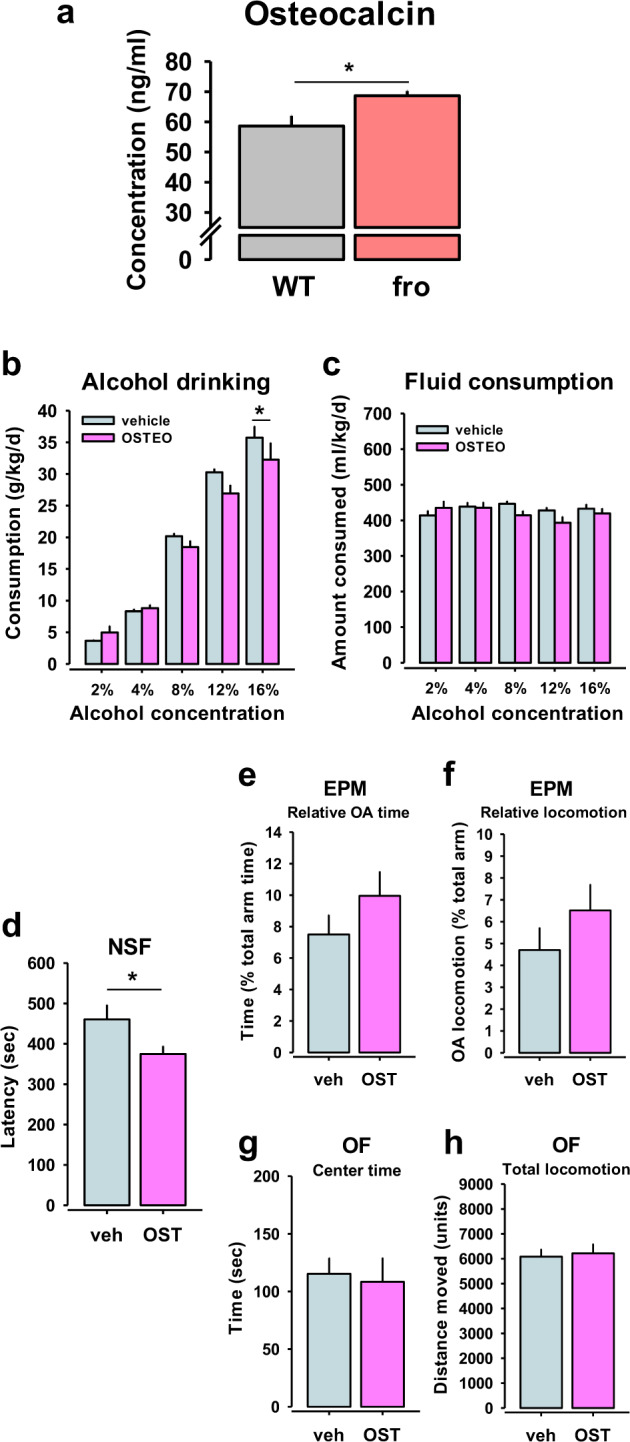
Neutral sphingomyelinase controls osteocalcin (OST) release. OST limits alcohol consumption and has beneficial effects on emotional behaviour. **a** Blood OST levels are upregulated in mice with reduced neutral sphingomyelinase (NSM) activity (fro) (*n* = 6 per group). Data are expressed as means + s.e.m. (*P < 0.05 vs. wild type (WT). **b** Chronic administration of OST (0.03 µg/30 g/h, s.c.) in naive C57Bl6J mice for 28 days via osmotic mini pumps attenuated alcohol consumption in a two-bottle free-choice test, but (**c**) did not affect total fluid intake (*n* = 8 per group). **d–g** Chronic OST had no effect on anxiety-related behaviour in mice in the elevated plus maze (EPM) or open field (OF) tests, but (**h**) reduced depression-like behaviour in the novelty suppressed feeding test (NSF) (*n* = 7–8 per group). Data are expressed as means ± s.e.m. (*P < 0.05, vs. vehicle (veh) control; OA open arm).

Since NSM was reported to be of crucial importance for bone mineralisation [11], we tested whether a developmental NSM deficit might trigger compensatory responses that maintain bone integrity [20]. We measured blood osteocalcin total levels. Carboxylated osteocalcin is an inductor of bone mineralisation by osteoblasts. Predominantly decarboxylated osteocalcin is released from osteoblasts. Once released to the blood, osteocalcin reaches receptors in peripheral organs. It may also cross the blood–brain barrier and reach the brain [21], where it can improve cognitive performance [22]. Here, we found that fro mice show an up-regulation of blood total osteocalcin levels (Fig. 4a). Besides rescuing bone development, this may suggest osteocalcin effects on behavioural control.

In order to identify a mechanistic link between NSM and osteocalcin production, we performed an RNA-Sequencing analysis in juvenile primary osteoblasts of fro and WT mice (Supplementary Note 1, Figs. S15–19). Results suggest that among the identified dysregulated pathways, in particular different expression of previously linked IL-6 and β_2_-adrenergic receptors might mediate the osteocalcin up-regulation during attenuated NSM activity.

### Alcohol drinking eliminates the advantageous effects of reduced NSM activity on emotional behaviour

**Fig. 5:**
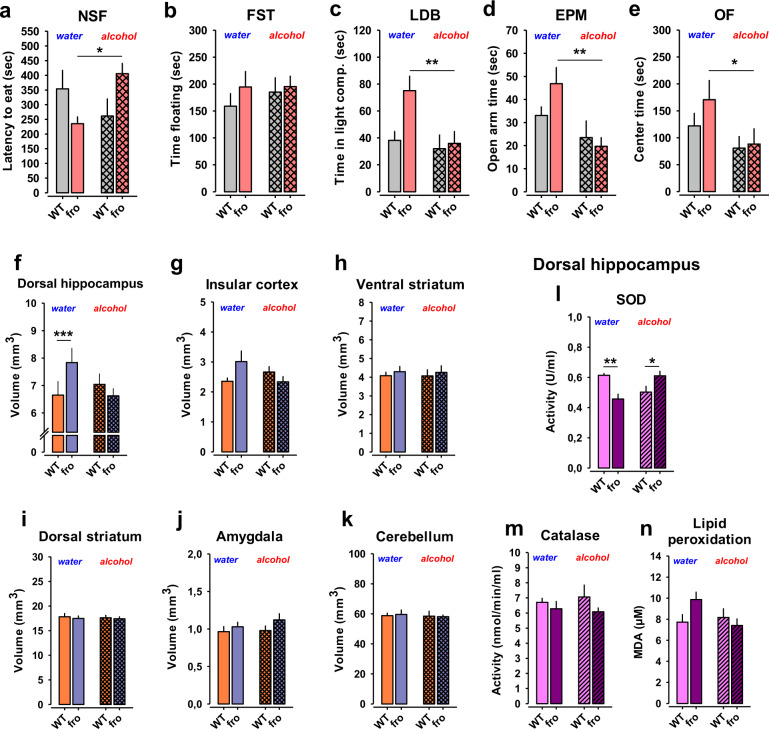
Neutral sphingomyelinase-2 (NSM) controls alcohol effects on emotional behaviour. **a** Free-choice alcohol consumption reversed the advantageous low-depression phenotype in female mice with reduced NSM activity (fro) in the novelty supressed feeding (NSF) test, but not in (**b**) the forced swim test (FST). Alcohol drinking eliminated the advantageous low anxiety phenotype of female fro mice in (**c**) the light–dark box test (LDB), (**d**) in the elevated plus maze test (EPM), and in (**e**) the open field test (OF) (*n* = 6–8 per group). Data are expressed as means + s.e.m. (*P < 0.05; **P < 0.01 vs. wild type (WT). **f** Fro mice show an enlarged volume of the dorsal hippocampus (DH) measured by magnet resonance imaging. Voluntary alcohol drinking reduced DH size in fro mice to WT levels mice. **g–k** There was no significant effect of reduced NSM activity or voluntary alcohol consumption on the size of other brain areas (*n* = 7–8 per group). Data are expressed as means + s.e.m. (***P < 0.001). **l** Superoxide dismutase (SOD) in the DH was significantly reduced in fro mice after water drinking. This effect was reversed after voluntary alcohol consumption (*n* = 7–8 per group). **m**, **n** There was no effect of NSM or alcohol on catalase activity or lipid peroxidation in the DH. All results show mean + SEM (*P < 0.05; **P < 0.01).

Next, we tested whether NSM mediates the interaction between the comorbid symptoms. We asked how NSM controls the effects of voluntary alcohol drinking on emotional state. We allowed fro and WT mice to voluntarily self-titrate their alcohol consumption for 6 weeks and tested the emotional state compared to mice that only drank water at all times. While voluntary alcohol drinking had little effects on emotional behaviour in WT mice, it enhanced depression-like and anxiety-like behaviour in fro mice in the NSF (Fig. 5a), LDB (Fig. 5c), EPM (Fig. 5d), and OF test (Fig. 5e), but not in the FST test (Fig. 5b). Thereby, alcohol consumption eliminated the emotional advantage of the fro mice in the EPM, LDB and OF tests, but not in the FST test (Figs. S20–22). These findings suggest that while reduced NSM activity causes a less depressed and less anxious emotional phenotype and elicits less alcohol drinking, it renders mice more susceptible to long-term negative emotional effects of alcohol. Altogether, these findings suggest that NSM controls the way in which alcohol drinking affects the emotional phenotype.

### Alcohol drinking eliminates the advantageous effects of reduced NSM activity on brain development

After observing an NSM control of the self-regulation of emotional state with alcohol, we searched for the neuronal mechanisms that may be responsible for this effect. We tested gross neuroanatomy of mice after chronic consumption of alcohol or water using magnet resonance imaging (MRI) [14, 15]. We found that the less depressed fro mice showed a significantly enhanced DH volume (Fig. 5f), and a tendency for an increase in the IC compared to WT mice (Fig. 5g), two areas highly related to emotional behaviour [23, 24] and alcohol abuse [15, 25, 26]. No significant changes were seen in the other investigated brain areas (Fig. 5h–k). This may explain the improved control of depression and anxiety levels in the fro mice. Chronic alcohol consumption decreased DH and IC volumes in fro to the level of WT mice (Fig. 5f, g), which is in line with the depressogenic and anxiogenic effect of alcohol drinking in these mice. The chronic alcohol consumption in this schedule had no effect on brain area volume in WT mice (Fig. 5f–k). The reduction in NSM activity reduced superoxide dismutase (SOD) activity in the DH compared to WT mice (Fig. 5l). This effect was reversed after alcohol consumption. No effect on catalase activity or lipid peroxidation were observed (Fig. 5l, n). These findings identify the DH as a locus where NSM mediates the interaction of alcohol with the emotional phenotype with altered oxidative stress management as a potential mechanism.

### NSM controls synaptic morphology in the hippocampus

**Fig. 6:**
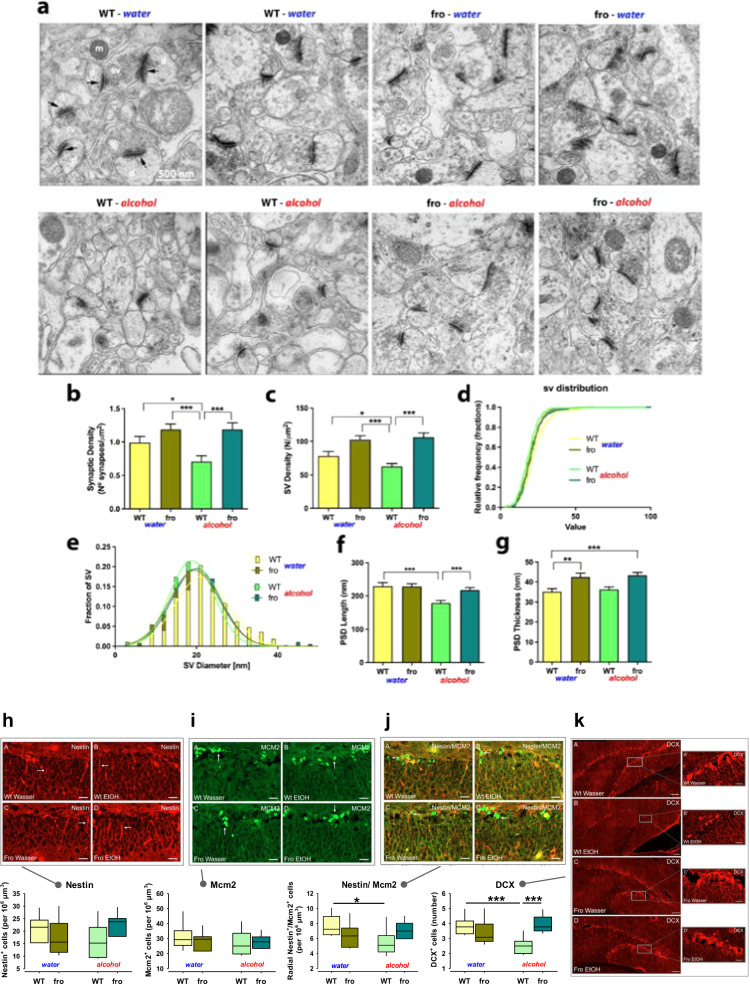
Neutral sphingomyelinase-2 (NSM) controls synaptic structure of mice and changes after alcohol (EtOH) consumption. **a** Representative electron micrographs of synapses in the dorsal hippocampus (DH) CA1 region in fro and wild type (WT) mice (black arrows indicate post-synaptic densities; d dendrite, sv synaptic vesicles, m mitochondria). **b** Synaptic density. **c** Synaptic vesicle density (*n* = 53 synapses). **d**, **e** Synaptic vesicle diameter (363 WT, 274 WT + EtOH, 589 fro and 654 fro + EtOH) vesicles. **f** Post-synaptic density length (46 WT, 58 WT + EtOH, 72 fro and 72 fro + EtOH post-synaptic densities). **g** Post-synaptic density thickness. Data are expressed as means + s.e.m. (*n* = 3 per group; *P < 0.05; **P < 0.01; ***P < 0.001). **h–k** Female fro mice do not show altered neurogenesis in the DH, but reduced susceptibility to the suppressive effects of alcohol (*n* = 4 per group; DCX-doublecortin). Data are expressed as means + s.e.m. (*P < 0.05; ***P < 0.001).

To further characterise the effects of NSM on DH structure, we used electron microscopy after chronic alcohol consumption. A reduction of NSM activity alone led to an increase in post-synaptic density (PSD) thickness, which may contribute to the observed overall increase in DH volume (Fig. 6a). Alcohol drinking reduced synaptic density (Fig. 6b), synaptic vesicle density (Fig. 6c, d, e), and PSD length (Fig. 6f), but had no effect on PSD thickness in WT mice (Fig. 6g). Reduced NSM activity completely prevented the adverse effects of chronic alcohol consumption on synaptic morphology in the DH. These findings are in line with beneficial effect of reduced NSM function alone, but may counteract DH volume loss after chronic alcohol consumption.

### NSM controls susceptibility of hippocampal neurogenesis to alcohol

As the DH is a developmental focus of NSM activity, which is susceptible to self-administration of alcohol and subsequent regulation of emotional state, we tested how NSM controls hippocampal neurogenesis. In female WT mice, chronic alcohol consumption in a free-choice paradigm had no effect on Nestin (neural stem cells and early intermediate progenitor cells; Fig. 6h) or Mcm2 (proliferating cells; Fig. 6i) expression alone, but significantly reduced Nestin/Mcm2 (activated neural stem cells; Fig. 6j) and doublecortin (DCX; immature neurons) expression (Fig. 6k). In line with previous reports in humans [27] and animals [28, 29], chronic voluntary alcohol consumption reduces the number of activated stem cells (Nestin/Mcm2), newborn neurons and neuroblasts (DCX) in mice. There was no difference between fro and WT mice when drinking only water, which suggests that a reduction of NSM activity has no effect on hippocampal neurogenesis. However, reduced NSM activity appeared to protect from negative alcohol effects on neurogenesis and may, thus counteract DH volume loss after chronic alcohol consumption.

### NSM controls monoaminergic signalling and responses to alcohol

**Fig. 7:**
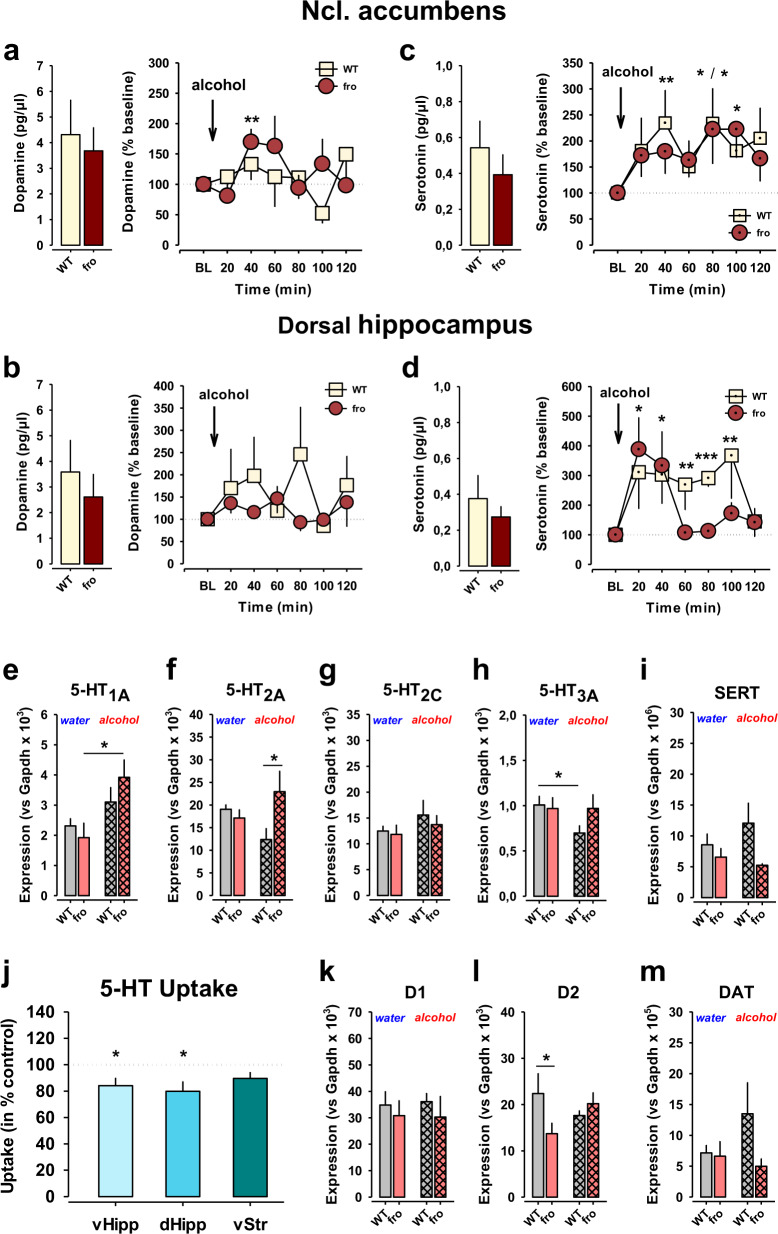
Neutral sphingomyelinase-2 (NSM) controls monoaminergic signalling in the brain. **a–d** Reduced NSM activity in mice (fro) has no effect on basal extracellular levels of dopamine (DA) and serotonin (5-HT) in the nucleus accumbens (Nac) and dorsal hippocampus (DH), but enhances DA response to alcohol (2 g/kg, i.p.) in the Nac (*n* = 16–27 per group). Data are expressed as means + s.e.m. of percent baseline (BL) (*P < 0.05; **P < 0.01; ***P < 0.001 vs. BL). **e–i** Effects of reduced NSM function and alcohol drinking on regulation of 5-HT receptor and transporter (SERT) mRNA expression in the ventral striatum (vStr) of mice. **j** Sphingomyelinase treatment inhibits 5-HT uptake in synaptosomes from ventral hippocampus (vHipp), dorsal hippocampus (dHipp), but less so in the vStr of mice. Values are expressed as percent of control ± s.e.m. (*n* = 5–9 per group). Data are expressed as means + s.e.m. of control levels taken as 100% (*P < 0.05; **P < 0.01 vs. control). **k–m** Effects of reduced NSM function and alcohol drinking on regulation of dopamine D1 and D2 receptor and transporter (DAT) mRNA expression in the vStr of mice (*n* = 3–6 per group). Data are expressed as means + s.e.m. (*P < 0.05; **P < 0.01).

The pharmacological reinforcing action of alcohol and transition to addiction are under the control of the dopamine (DA) and serotonin (5-HT) systems [30, 31]. Previously, a strong interaction of acid sphingomyelinase (ASM) with brain monoamine tissue level control and dynamic responses was reported. Enhanced ASM activity and ceramide abundance reduced tissue DA and 5-HT levels in the nucleus accumbens (Nac) and DH of mice, which resulted in a depressive phenotype [19, 23]. This went along with potentiated DA responses to alcohol in both structures [32]. Voluntary alcohol drinking reduced ASM hyperactivity, normalised monoamine tissue levels and attenuated the depression [19]. Here we used in vivo microdialysis to test extracellular monoamine levels and responses to an acute alcohol challenge in mice in previously identified key areas of NSM action. We did not observe differences in the basal extracellular level of DA and 5-HT in the DH or Nac when compared between fro and WT mice (Fig. 7a–d). Acute alcohol administration increased extracellular DA levels. This effect was enhanced in the Nac (Fig. 7a), but attenuated in the DH of the fro mice (Fig. 7b). The alcohol-induced peak increase in extracellular 5-HT levels in the Nac (Fig. 7c) and DH (Fig. 7d) was not changed in fro compared to WT mice.

While alterations in neurotransmitter levels indicate an NSM control of monoaminergic signalling in reward and reinforcement related brain areas, synaptic throughput also requires receptor and transport mechanisms [33]. Monoamine receptor conformation and function as well as transporter efficacy crucially depend on sphingolipid membrane domains [34, 35]. Therefore, we measured mRNA expression levels of major receptors and transporters involved in dopaminergic and serotonergic signalling in the ventral striatum (vStr) of mice. We found that chronic alcohol drinking decreased *5-HT*_*3A*_ receptor mRNA expression, but did not affect expression of *5-HT*_*1A*_, *5-HT*_*2A*_, *5-HT*_*2C*_ (Fig. 7e–h) and *DA D1* and *D2* receptor mRNA in WT mice (Fig. 7k–l). Reduced NSM activity led to a reduction in *D2* receptor mRNA expression. However, it also prevented the alcohol effects on *5-HT*_*3A*_-receptor mRNA, but enhanced mRNA expression of *5-HT*_*1A*_ receptors. The expression of the DA transporter (*DAT*) and 5-HT transporter (*SERT*) were affected neither by alcohol nor by NSM deficiency (Fig. 7i, m). In contrast, enhancing sphingomyelinase activity resulted in a significant reduction of 5-HT uptake in synaptosomes of the ventral hippocampus (VH) and DH, but not in vStr of mice (Fig. 7j).

Sphingolipids form lipid rafts and signalling platforms at the synapse [36], which harbour monoamine and other receptor proteins [34, 35, 37]. Thus, we investigated the vStr lipidome after voluntary alcohol or water consumption [38]. Neither reduced NSM activity nor voluntary alcohol drinking yielded a significant effect on lipid landscape in the vStr (Figs. S23–26).

Although Nac DA responses to alcohol are enhanced in fro mice, reduced D2 receptor expression may balance NSM control of the incentive properties of alcohol. This may explain why the conditioned reinforcing effects are not altered in mice with reduced NSM activity.

### Osteocalcin reduces alcohol consumption

Reduced NSM activity led to an increase in blood osteocalcin. In order to test whether the bone–brain signal interacts with alcohol consumption, we tested how increased osteocalcin would modulate alcohol consumption. In naïve mice, osteocalcin treatment for 28 days (0.03 µg/h, s.c.), using osmotic mini pumps, reduced alcohol consumption (Fig. 4b), but did not affect total fluid consumption (Fig. 4c).

### Osteocalcin has antidepressant, but not anxiolytic effects

We further tested how increased osteocalcin would modulate affective behaviour. In naïve mice, osteocalcin treatment for 28 days (0.03 µg/h, s.c.), using osmotic mini pumps, had an antidepressant effect in the NSF (Fig. 4d), and a trend towards anxiolytic effects in the EPM (Fig. 4e), while it did not change general locomotor activity in the EPM (Fig. 4f) or OF (Figs. 4g, h, S27). As osteocalcin effects emerged largely in the same direction for bone mineralisation (protection), alcohol drinking (protection), and antidepressive action (enhanced), it may also suggest osteocalcin as a potential treatment specifically for this symptom trias in humans (Figs. S28, 29).

## Discussion

Alcohol addiction, major depression/anxiety and osteoporosis are considered as separate diseases, which show a high co-morbidity. Here we discovered a shared genetic basis for this symptom trias. This affects the behavioural phenotypes and endophenotypes of each single disorder as well as some of their interactions. We identified spontaneous genetic variations in the *SMPD3* gene, which are related to alcohol abuse, affective behaviour, and bone mineralisation in humans. Furthermore, an *SMPD3* haplotype polymorphism was related to hippocampal size in humans. A functional analysis of NSM activity in genetic and pharmacological mouse models showed that chronic attenuation of NSM activity decreased alcohol consumption. Pharmacological challenge experiments confirmed this role of NSM in alcohol drinking. NSM is not required for the establishment or the expression of alcohol’s conditioned rewarding effects. Also the sedating properties of alcohol are not under NSM control. Mice with reduced NSM activity showed less depression- and anxiety-like behaviour. NSM is highly expressed in the brain [39, 40]. Mice without NSM function in neurons showed a perturbed Golgi secretory pathway, dysproteostasis and impaired learning and memory [41, 42]. In the present study, mice with partially reduced NSM function drank less alcohol, possibly because alcohol reduced their affective advantage, i.e. it had predominantly depressogenic and anxiogenic effects. This suggests an NSM control of the relationship between alcohol consumption and self-regulation of emotional states as shown in clinical and preclinical studies [43–45]. The reason why NSM deficient mice still consumed alcohol was most likely the enhanced dopaminergic and preserved serotonin activation, which counteracted the aversive emotional effects.

The beneficial affective phenotype coincided with an enhanced DH volume and enhanced neocortex-amygdala resting state connectivity. The increased DH volume may be supported by the observed enlargement of synaptic structures. NSM has its highest expression in the hippocampus where it is highly active during development. NSM enhances the excitability of hippocampal CA1 neurons [46, 47]. Individual differences in NSM activity in this brain region predispose for efficient learning and adaptations in particularly appetitively motivated long-term memory tasks [42, 48, 49]. An enhanced capacity of the DH may serve better stress resistance and reduce depression-like behaviour, and provide resilience during initiation of alcohol use [50]. This supports the human association of *SMPD3* with hippocampal volume. We observed that voluntary alcohol drinking did not only have depressogenic effects, but also reduced DH volume to control level. A reduction in NSM activity had no major effects on hippocampal neurogenesis. However, it changed sensitivity to alcohol’s effects in that it attenuated sensitivity to the suppressive effects of alcohol on neurogenesis.

**Fig. 8:**
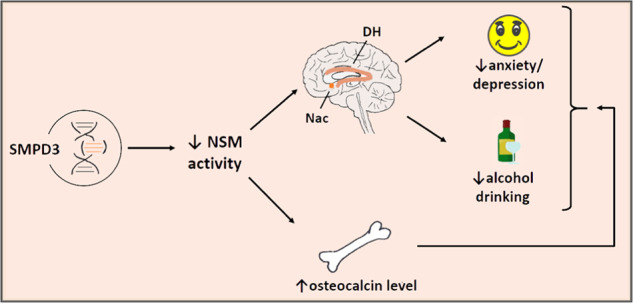
Schematic link between the *SMPD3* gene, behaviour and bone function. Neutral sphingomyelinase (NSM) and its coding gene *SMPD3* is a single source for multiple pathways interlinking alcohol abuse—depression/anxiety—bone disorder (DH dorsal hippocampus, Nac nucleus accumbens).

In humans, we found a *SMPD3* association with BMD, which expands a previously reported role in bone mineralisation from fragilitas ossium symptom patients to healthy subjects [11]. We found an up-regulation of osteocalcin, a potential compensatory signal, in the blood of NSM deficient mice. An RNA-Sequencing analysis of osteoblasts suggested several NSM-dependent pathways that may account for this effect, including IL-6 receptors and β2-adrenoceptors, which can drive osteocalcin signalling [51, 52]. Osteocalcin regulates bone mineralisation [53]. It is released from osteoblasts into circulation. Besides its effects in other target organs and on metabolism, it is also a signal from the skeletal system to the brain. It can cross the blood-brain barrier and reaches osteocalcin- and orphan receptors in the hippocampus, ventral tegmental area and other brain structures [54]. A role of osteocalcin and some of its receptors in learning and memory as well as in emotional behaviour and alcohol abuse was previously reported [21, 55, 56]. In this study, chronic osteocalcin treatment reduced alcohol consumption and improved the emotional phenotype alike. In that the bone–brain signal mimicked the fro phenotype in those behaviours (Fig. 8).

NSM is a crucial member of the sphingomyelinase pathway of ceramide systems. However, compensatory changes in the activities of other enzymes of this pathway, particularly ASM or acid and neutral ceramidases, might also be involved in the observed behavioural endophenotype. These enzymes were recently shown to contribute to drug addiction [19, 32, 57], emotional phenotypes [8, 58], and cognitive behaviours in rodents [42, 59]. However, NSM is the only enzyme of ceramide synthesis and metabolism shown to mediate bone–brain signalling in the co-morbidity trias of alcohol addiction—depression/anxiety—osteoporosis.

## Conclusions

We therefore propose *SMPD3* and its coded protein NSM as a joint base for the frequently comorbid symptom trias of alcohol addiction—depression/anxiety—osteoporosis. NSM mediates single disease symptoms as well as their reciprocal interaction along distinct brain pathways and by bone-derived osteocalcin signalling. Targeting of NSM and osteocalcin may, thus, serve as a suitable treatment for the co-morbidity trias of alcohol addiction—depression/anxiety—osteoporosis.

## Methods

### Neutral sphingomyelinase activity in serum of patients with alcohol dependence

A sex-balanced cohort of 200 alcohol-dependent in patients seeking withdrawal treatment was recruited for the bi-centric, cross-sectional, and prospective Neurobiology of Alcoholism (NOAH) study in parallel to 240 age-matched healthy controls following a multi-step screening procedure in 2013–2014 [60, 61]. NSM activity in the serum was determined as described previously [62, 63] (Supplementary Methods).

### Human association study

#### Participants

456,693 participants (56.0% female) with complete genotype and behavioural data were drawn from UK Biobank, a large cohort of United Kingdom residents aged 40–69 years [9]. UK Biobank obtained informed consent from all participants (ref: 11/NW/0382; UK Biobank Resource under Application Number 26503).

#### Genotyping and phased haplotypes

A total of 488,377 blood samples were genotyped on the UK BiLEVE Axiom array (*N* = 49,950) and the UK Biobank Axiom array (*N* = 438,427) [64]. Phased haplotypes were obtained using the SHAPEIT3 software [10] and extracted for the region covering the SMPD3 gene (GRCh37, 16:68,387,230–6,848,7409). Haplotype phases with minor haplotype frequency (MHF) <0.01 were excluded, resulting in 26 haplotypes available for analysis (Table S1). Alcohol intake frequency, drinker status, nervous feelings and worrier/anxious feelings were measured using touch-screen questionnaires. Total BMD of the left and right femurs were derived from dual-energy X-ray absorptiometry. Structural MRI data was acquired [65] (Supplementary Methods).

### Alcohol-related behaviour in mice

#### Animals

Transgenic female mice with a reduction in neutral sphingomyelinase (NSM) activity (heterozygous NSM knock out, fro, fragilis ossium) and female WT mice from the same litter aged 8–15 weeks were used in the experiment [11]. Only heterozygous NSM knock out were used in these experiments. Carriers of a homozygous NSM knock out mutation develop severe osteogenesis imperfecta [11], while a heterozygous mutation is more likely to be represent the natural variance of NSM activity. Furthermore, as previous studies on the brain–bone signalling were solely performed on female animals [22, 55], our study also focused on females. All experiments were carried out in accordance with the National Institutes of Health guidelines for the humane treatment of animals and the European Communities Council Directive (86/609/EEC) and approved by the local governmental commission for animal health (Regierung von Unterfranken). Alcohol drinking, taste preference blood alcohol concentration and the loss of righting reflex were measured as previously described [19, 66–68] (Supplementary Methods).

#### Alcohol drinking—pharmacology

The effects of an acute inhibition of NSM by a specific inhibitor ES048, kindly provided by EMS and CA, on the drinking pattern was studied on naïve C57Bl6J female mice (*n* = 30) in the model of intermittent 20 vol.% alcohol consumption [16, 69] (Supplementary Methods).

### Alcohol conditioned place preference

The establishment of CPP for alcohol was tested in treatment naive adult fro and WT mice (*n* = 11/15/group) [19, 70]. In order to test whether the NSM effect on the CPP development is an acute effect or long-term genetically driven effects based on developmental lack of NSM function, we tested the effects of an acute pharmacological inhibition of NSM activity on this parameter. For this purpose, C57Bl6J mice (*n* = 9/10/group) were treated with two selective NSM inhibitors, ES048 (400 ng/kg) and GW4869 (2 mg/kg), as described above (Supplementary Methods).

### Emotional behaviour in fro mice

8–14-week-old female mice (*n* = 11/group) were tested using a battery of behavioural tests in the following order: open field, light–dark box, elevated plus maze, novelty suppressed feeding, forced swim, and SPTs [14, 15, 18, 19] (Supplementary Methods).

### Emotional behaviour—pharmacology

To check the effects of an acute pharmacological inhibition of NSM on the emotional state of animals, naïve C57Bl6J female mice (*n* = 8/group) were continuously administered with a selective NSM inhibitor GW4869 in the dose of 2 mg/kg/day (i.p.) via Alzet osmotic mini pumps. After the implantation, the animals were single housed and left for recovery for 6 days. Thereafter the animals were tested in a battery of behavioural tests including the open field, elevated plus maze, and novelty supressed feeding test as described above. Mice were returned to their home cages at the end of each test and allowed to recover for at least 1 day before further testing. Behaviours for all tests were recorded for subsequent scoring [71, 72].

### Mouse resting state fMRI

FMRI measurements were conducted on a 4.7 T small-animal MRT. A fast whole-brain coronal T2-weighted rapid acquisition relaxation enhanced sequence was recorded. The Pearson correlation coefficient r was calculated between the average time course of each seed region and those of all voxels within the brain. Significant correlations were determined for each correlation volume using false discovery rate (*q* = 0.05) (Supplementary Methods).

### Osteocalcin measurement

Circulating levels of osteocalcin (bone Gla protein) were measured in the blood serum of naïve female fro and WT mice (*n* = 8–10/group).

### Osteocalcin treatment in mice

Recombinant mouse osteocalcin (Cusabio; MBS948782) in the dose of 0.03 µg/h administered continuously to female C57Bl6J mice (*n* = 8/group) via Alzet osmotic mini pumps. Thereafter the animals were tested in a battery of behavioural tests including the open field, elevated plus maze, and novelty supressed feeding as described above. After the last behavioural test, the animals were exposed to alcohol on the model of two-bottle free-choice paradigm [71, 72] (Supplementary Methods).

### RNA-seq analysis in mouse osteoblasts

Primary osteoblasts were isolated from calvariae of 2-3-day-old neonatal WT and fro mice. RNA was isolated from the osteoblasts using the RNeasy Micro Kit (Qiagen). Sequencing libraries were generated from 1 µg high quality RNA using the TruSeq Stranded mRNA Kit (Illumina, San Diego, U.S.A.). Libraries were sequenced on a HiSeq 2500 platform (Illumina, San Diego, U.S.A.). Differentially expressed genes were determined using a negative binomial model (Supplementary Methods).

### Emotional behaviour after alcohol drinking

Alcohol drinking was established in naïve female fro and WT mice (*n* = 7/8/group) using a two-bottle free-choice drinking paradigm. After 12 days drinking of 16 vol.% alcohol, animals were tested in a battery of behavioural tests including open field, elevated plus maze, novelty suppressed feeding, and forced swim test. The mice continued drinking 16 vol.% alcohol in their home cages throughout the whole testing [19, 63].

### Mouse structural MRI and bone CT

Anatomical small-animal magnetic resonance imaging (MRI) was used to investigate the effects of alcohol on the volume of DH, IC, ventral and dorsal striatum, amygdala, and cerebellum in animals with NSM hypoactivity. Alcohol drinking was established in naïve female Fro and WT mice (*n* = 7/8/group) using a two-bottle free-choice drinking paradigm. After 12 days drinking of 16 vol.% alcohol, animals were exposed to MRI. Using CT, bone density was determined in various osseous structures by assessing the Hounsfield Units of the respective bone (Supplementary Methods).

### Oxidative stress measurements

The effects of alcohol on the oxidative stress in the brain was evaluated in female fro and WT mice (*n* = 7/8/group). Alcohol drinking was established in naïve animals using a two-bottle free-choice drinking paradigm [19]. After 12 days drinking of 16 vol.% alcohol, animals were sacrificed and the brains were isolated. Superoxide dismutase and catalase activity were measured [73, 74] (Supplementary Methods).

### Electron microscopy of hippocampus slices

The effects of alcohol on the morphology of hippocampal synapses were evaluated in female fro and WT mice (*n* = 3/group). Alcohol drinking was established in naïve animals using a two-bottle free-choice drinking paradigm. After 12 days drinking of 16 vol.% alcohol, fro mice and WT littermates were intracardially perfused with PBS and glutaraldehyde. Serial ultrathin sections of the CA1 region were collected and examined with a transmission electron microscope (Supplementary Methods).

### Neurogenesis measurement in mice

The effects of alcohol on neurogenesis in the hippocampus was evaluated in female fro and WT mice (*n* = 4/group). Alcohol drinking was established in naïve animals using a two-bottle free-choice drinking paradigm. After 12  days drinking of 16 vol.% alcohol, animals were intracardially perfused with PBS and fixative and the brains were isolated. Stage-specific marker expression of Nestin, MCM2, and DCX were assessed in the coronal sections containing medial dentate gyrus (Supplementary Methods).

### In vivo microdialysis in mice

The experiment was performed on naïve female fro and WT mice (*n* = 7–10/group). Two guide cannulas were aimed at the DH and the Nac [14, 32, 75]. First three miocrodialysis samples were used to measure baseline quantities of the neurotransmitters DA and 5-HT [71]. After 1 h, three samples were collected as a baseline for the alcohol stimulus followed by an i.p. injection of alcohol, and further six samples were collected. Once the microdialysis was completed, animals were sacrificed, brains isolated, and the localisation of the probes was verified. All samples were analyzed using HPLC with electrochemical detection [32, 71] (Supplementary Methods).

### Monoamine receptor mRNA expression in the mouse brain

Alcohol drinking was established in naïve female fro and WT mice using a two-bottle free-choice drinking paradigm. After 12 days drinking of 16 vol.% alcohol, animals were sacrificed and ventral striatum was isolated. Total RNA was isolated and quantitative real-time PCR was performed (Supplementary Methods).

### Serotonin uptake in mouse brain synaptosomal preparations

Crude synaptosome fractions were prepared [76] in that mouse brains were rapidly removed and dissected. Ventral and DH and ventral striatum were homogenised. Counts for SERT specific [3H]-5-HT uptake were determined (Supplementary Methods).

### Lipidomics

Alcohol drinking was established in naïve female fro and WT mice using a two-bottle free-choice drinking paradigm. After 12 days drinking of 16 vol.% alcohol, animals were sacrificed and DH was isolated. Lipidomic profiling was performed using Ultra Performance Liquid Chromatography-Tandem Mass Spectrometry [77–80] (Supplementary Methods).

### Statistical analyses

Data were examined with one- or two-way analysis of variance, for repeated measures where appropriate. For single group and time point effects, pre-planned comparisons were calculated using Bonferroni-corrected LSD tests or *t* tests. A *P* value of 0.05 was considered indicative of statistical significance.

## Supplementary information

Supplementary material

Supplementary tables
