# Supplementary material for: Neutral sphingomyelinase mediates the co-morbidity trias of alcohol abuse, major depression and bone defects
Source: Mol Psychiatry. 2021 Sep 28;26(12):7403–16. doi: 10.1038/s41380-021-01304-w (PMC8872992; doi:10.1038/s41380-021-01304-w)
Supplement: Supplementary file 1 — Supplementary material [file 41380_2021_1304_MOESM1_ESM.pdf]

Supplementary information

Supplementary Methods

Neutral sphingomyelinase activity in serum of patients with alcohol dependence

A sex-balanced cohort of 200 alcohol-dependent in-patients seeking withdrawal treatment was recruited for the bicentric, cross-sectional, and prospective Neurobiology of Alcoholism (NOAH) study in parallel to 240 age-matched healthy controls following a multi-step screening procedure in 2013-2014 [60,61]. The study was approved by the Ethics Committee of the Medical Faculty of the Friedrich-Alexander University Erlangen-Nürnberg (ID 81_12 B, 19 April 2012). Blood drawings were performed in the morning and during 24 to 72 hours of abstinence in patients, and serum samples were frozen at -80°C within 2h. NSM activity in the serum per time and per volume was determined using the fluorescent substrate BODIPY-FL-C12-SM (N-(4,4-difluoro-5,7-dimethyl-4-bora-3a,4adiaza-s-indacene-3-dodecanoyl) sphingosyl phosphocholine, Invitrogen/Life Technologies) as described previously [62,63].

Supplementary Methods

Human association study

Participants

456,693 participants (56.0% female) with complete genotype and behavioural data were drawn from UK Biobank, a large cohort of United Kingdom residents aged 40-69 years [9]. UK Biobank obtained informed consent from all participants (Ref: 11/NW/0382). This research has been conducted using the UK Biobank Resource under Application Number 26503.

Genotyping and Phased haplotypes

A total of 488,377 blood samples were genotyped on the UK BiLEVE Axiom array (N=49,950) and the UK Biobank Axiom array (N=438,427). Details on genotyping, quality control and haplotype estimation procedures can be found on the UK Biobank website (https://biobank.ctsu.ox.ac.uk/crystal/label.cgi?id=100314) and Bycroft et al (2018) [64]. Phased haplotypes were obtained using the SHAPEIT3 software [10] and extracted for the region covering the SMPD3 gene (GRCh37, 16: 68387230 – 68487409). Haplotype phases with minor haplotype frequency (MHF) <0.01 were excluded, resulting in 26 haplotypes available for analysis (**Table S1**).

Behavioural Measures Participants completed touch-screen questionnaires during their visit to the assessment centre. Details for each variable is described on the UK Biobank Data showcase (http://biobank.ctsu.ox.ac.uk/crystal/) under the respective Data Field. Alcohol intake frequency was measured by asking the participants “About how often do you drink alcohol” and was coded on a 5-point scale ranging from “Daily or almost daily” to “Never”. Alcohol drinker status was coded on a 3-point scale (0: Never, 1: Previous, 2: Current). Nervous feelings and Worrier/Anxious feelings were measured by asking the participant “Would you call yourself a nervous person” and "Are you a worrier" respectively and were coded as binary variables (1: Yes, 0: No). Frequency of depressed mood was measured by asking the participant “Over the past two weeks, how often have you felt down, depressed or hopeless” and was coded on a 4-point scale ranging from “Not at all” to “Nearly every day”.

Supplementary Methods

Human association study

Physical Measurements Of the initial sample 4515 participants (52.9% female) who had complete genetic, bone mineral density and neuroimaging data were included in the analyses involving physical measures.

Bone Mineral Density Total bone mineral density of the left and right femurs were derived from dual-energy X-ray absorptiometry (DXA). Details on the procedure for DXA measurement and QC protocol can be found on the UK Biobank Data showcase under Category 103.

Neuroimaging Structural MRI data was acquired using a standard Siemens Skyra 3T running VD13A SP4, with a standard Siemens 32-channel RF receive head coil. Details on acquisition protocols, image processing pipeline and derived measures can be found on the UK Biobank Brain Imaging Documentation (biobank.ctsu.ox.ac.uk/crystal/docs/brain_mri.pdf). This study made use of imaging-derived phenotypes (IDPs) generated by an image-processing pipeline developed and run on behalf of UK Biobank [65]. Grey matter volume of the insula and hippocampus were generated using the FAST (FMRIB's Automated Segmentation Tool) grey matter segmentation.

Data Analysis All analyses controlled for the effects of recruitment site, age, gender and 10 UK Biobank genetic principal components (to further control for ethnic stratification). Analyses involving brain structure controlled for total intracranial volume. All analyses were performed with R, version 3.5.1 (https://cran.r-project.org), and a significant threshold set at <0.05 was applied.

Haplotype analyses For each haplotype phase, a Hotelling's t-test was conducted to analyse the relationship between haplotype and the behavioural outcomes. Univariate t-tests were conducted between each haplotype phase and total bone mineral density of the left and right femur respectively. A Hotelling's t-test was also conducted to determine the relationship between Hap26 and grey matter volume of the insula and hippocampus. FDR-corrected p-values are reported.

To determine the relationship between SMPD3 and bone density, we conducted a univariate analysis between each haplotype phase and total bone mineral density (BMD) of the left and right femurs. We found significant associations between Hap26 and both the left and right femur BMD (left: t=3.109, p=0.04909; right: t=3.329, p=0.04563; **Table S4**, **Figure 1**).

Considering that SMPD3 KO mice exhibited bone deficiencies and different sized dorsal hippocampus and insular cortex, we went on to investigate this relationship in human participants. Using the same haplotype that had a significant association with bone density, Hap26, we conducted a Hotelling's t-test with grey matter volume of the insula and hippocampus. We found a significant association between Hap26 and grey matter volume (F=3.63, p=0.02645), with hippocampal volume being the significant region of interest (F=7.24, p=0.007147; **Figure 1**).

Supplementary Methods

Animals

The mice were kept under a normal light-dark cycle (2-5 per cage or single housed when required by the experiment; light off 07:00 pm – 07:00 am).

Supplementary Methods

Alcohol-related behaviour in mice

Alcohol drinking Alcohol drinking pattern was tested in naïve fro and WT mice (n=8/group) using a two-bottle free-choice drinking paradigm. Mice were single housed and each cage was equipped with two bottles constantly available, one of which contained tap water and the other bottle contained alcohol in various concentrations. After an acclimatization period of two weeks to establish a water drinking baseline, animals received alcohol at increasing concentrations of 2, 4, 8, 12, and 16 vol.% for 4 days each. Bottles were changed and weighed daily. The consumed amount of alcohol and water relative to body weight and the preference vs. water were measured [19,66,67].

Tate preference Sucrose (0.2 and 2%) and quinine (2 and 20 mg/dl) preferences were measured in a two-bottle free-choice test vs. water three days after the last alcohol exposure. Each dose was offered for 3 days with the position of the bottles being changed and weighed daily with a one-day wash out between the sucrose and quinine testing [19,66,67].

Blood alcohol determination Alcohol naïve female fro (n=5) and WT mice (n=4) were injected with alcohol (3.0 g/kg, 20ml/kg, i.p.) and 20 µl blood samples were taken from the submandibular vein 1, 2, and 3 hours after the injection. The blood samples were directly mixed with 80 µl 6.25 % (w/v) trichloroacetic acid. After centrifugation 15 µl of the supernatant were subjected to enzymatic alcohol determination using the alcohol dehydrogenase method as described elsewhere [68].

Loss of righting reflex Alcohol naïve female fro (n=6) and WT (n=5) animals were injected with 3.5g/kg (i.p.) alcohol in saline (20ml/kg) to induce a loss of the righting reflex (LORR), and immediately placed in an empty cage. LORR was registered after the animal became ataxic and stopped moving for at least 30 seconds. Then the animal was placed on its back. Recovery from alcohol administration was considered when the animal was able to right itself three times within a minute. A 2h cut off was used. Time taken for the animal to lose its righting reflex, and time to recovery from alcohol's effect were recorded [19,66].

Supplementary Methods

Alcohol-related behaviour in mice

Alcohol drinking – pharmacology The effects of an acute inhibition of NSM by a specific inhibitor ES048 (kindly provided by E.M. Saied and Ch. Arenz, Institute of Chemistry, Humboldt-University, Berlin) on the drinking pattern was studied on naïve C57Bl6J female mice (n=30) in the model of intermittent 20 vol.% alcohol consumption. The animals were single housed, while two drinking bottles with water were constantly available for the first two weeks. Then, 20 vol.% alcohol drinking was induced for 6 weeks in an intermittent access schedule where animals received alcohol in one bottle only Thursday, Saturday, and Monday of each week for 24 hours. During other days, animals had access to 2 bottles with tap water. The experiment tested the effects of ES048 during ongoing consumption, i.e., during alcohol access. After 6 weeks of induction of stable alcohol consumption, each animal received an intraperitoneal injection of ES048 dissolved in saline (4 or 400 ng/kg) or vehicle (n=10 mice/group) once a week 1 h before the introduction of 20% alcohol. The procedure was repeated for 3 weeks. The dose of ES048 was pretested on cultured T-cells [16] and living animals (**Suppl. Figure 4**). The consumed amount of alcohol relative to body weight and the preference vs. water were measured during the whole experiment [69].

Supplementary Methods

Alcohol conditioned place preference

The establishment of conditioned place preference (CPP) for alcohol was tested in treatment naive adult fro and WT mice (n=11/15/group). The two TSE Place Preference test boxes (TSE Systems) with 3 compartments were composed of non-transparent polyvinyl chloride with standard inside dimensions (40 × 15 cm). The floor of the left chamber was covered with a smooth black rubber mat and the floor of the right chamber was covered with a patterned black rubber mat, whereas the centre chamber was not covered and coloured white. The system automatically recorded the number of entries, the time spent in the compartment and the distance moved in each compartment for each trial using high-resolution infrared sensors. An unbiased design was used in which half of the mice were conditioned to their preferred compartment, and half to their non-preferred compartment. Animals were injected with either saline or alcohol (2 g/kg, 10 ml/kg, i.p.) immediately before each trial, and placed into the CPP boxes for 20 min for conditioning/pseudo-conditioning trials [19,70].

The CPP experiment involved four phases: a habituation trial (one session), a pre-conditioning testing (baseline, Bl), conditioning trials (14 sessions) and preference tests (3 sessions, T1–T3). Habituation: A habituation session was intended to acclimatize the mice to the test procedure and apparatus prior to commencing the experiment. Mice were injected with saline and placed in the centre compartment with free access to all three compartments for 20 min. Baseline test: The pre-test was designed to establish a baseline level of preference for each individual. Mice were conditioned to either their preferred or non-preferred compartment using a counterbalanced experimental design. Mice were injected with saline and placed in the centre compartment with free access to all three compartments for 20 min. Conditioning trials: Conditioning trials were performed in pairs; odd numbered pairings were conditioned with alcohol (2 mg/kg, i.p.) and even numbered pairings were conditioned with saline, balanced across groups. All animals received seven pairings with saline and seven pairings with alcohol. Mice were injected with either saline or alcohol and introduced into one of the two conditioning compartments, with restricted access, for 20 min. Preference tests: Preference tests were systematically performed after one (T1), three (T2) and seven (T3) conditioning/pseudo-conditioning trials to monitor the time course of CPP establishment. Before each test, mice were injected with saline and placed in the centre compartment with free access to all three compartments for 20 min. The time spent in the conditioning/pseudo-conditioning compartment, the entries into these compartments, and locomotor activity were measured to estimate mechanisms driving a potential CPP [70].

Supplementary Methods

Emotional behavior in fro mice

All tests were performed on separate days between 09:00 and 16:00 h. Mice were tested in a pseudorandom order and were moved to the behavioral suite adjacent to the housing room at least 1 hour before testing. Each test apparatus was cleaned with 70% ethanol between subjects to avoid any olfactory cues influencing behaviors. Mice were returned to their home cages at the end of each test and allowed to recover for at least 2 days before further testing. Behaviors for all tests were recorded for subsequent scoring [18,19].

Open Field test Each mouse was placed in a square white acrylic arena (50×50 cm), facing an outer wall, for 20 min and allowed to freely explore the arena. White light of 100 lx was evenly distributed across the arena during testing. Video recordings were taken and analyzed using Biobserve Viewer III (Biobserve GmbH, Germany). A virtual square of equal distance from the periphery (36×36 cm) was defined as the „central zone" in order to determine the number of entries, and time (s) spent in the central zone. Distance moved in the outer and central zones (cm), number of entries and time spent in the central zone were registered.

Elevated Plus Maze The elevated plus maze was constructed from black opaque acrylic with white lining on the floor, each arm measuring 30×5 cm and the central platform 5×5 cm. One set of arms, opposing one another, were enclosed completely by a wall of opaque acrylic, 15 cm high, while the other set was open with a ledge of 0.5 cm either side of the arms. The open arms were brightly lit (100 lx), while the closed arms were partially protected from light (15-20 lx). The maze was elevated 50 cm from the ground on a transparent acrylic stand. Each mouse was placed on the central platform, facing a closed arm, and allowed to freely explore the maze for 5 min. Biobserve Viewer III tracking software (Biobserve GmbH, Germany) was used to record locomotor activity during the test (distance moved in the open and closed arms), and the number of entries into the closed and open arms and time spent in them. An arm entry was counted when two paws had entered an arm, and an arm exit was determined when two paws had left the arm.

Light dark box For the light-dark test, a box of white acrylic was used (50×50 cm). The box was partitioned with a white acrylic wall, so that one-third of the total area was dark („dark chamber"). The remaining two thirds were brightly lit (100 lx) with white light, which served as the „light chamber". A small entry door within the partition (5×7 cm), allowed mice to move freely between chambers. Each mouse was placed into the dark chamber facing the end wall parallel to the partition. Activity was recorded for the following 5 min using Biobserve Viewer III (Biobserve GmbH, Germany). Locomotor activity (distance moved in cm) and time (s) in the dark and light chambers, and latent period of first entrance into the light chamber were measured. A single transition was counted when two paws had entered a chamber.

Supplementary Methods

Emotional behavior in fro mice

Novelty suppressed feeding Animals were deprived from food for 24 h before novelty-suppressed feeding test. After food deprivation each mouse was put in the corner of a square white acrylic arena (50×50×50 cm), facing an outer wall. White light of 100 lx was evenly distributed across the arena during testing. A piece of standard food was placed in the center of the arena. Video recordings were taken and analyzed using Biobserve Viewer III (Biobserve GmbH, Germany). The time (s) before a mouse began eating after the fasting period (24 h) and the distance moved before eating were registered.

Forced swim test For the forced-swim test, each mouse was placed into a glass transparent cylinder (17 cm diameter, 18 cm height) filled with water (12 cm, 25±1°C) for 15 min. Then an animal was returned to the home cage. After 24 h mice were again placed in this cylinder with water for 5 min. The latency of first floating, and total floating time during days 1 and 2 were recorded manually.

Sucrose preference test Animals were single housed and had access to two bottles with water 7 days before the sucrose preference test. At day 8, water in one bottle was replaced by 2% sucrose solution, and the position of bottles with water and sucrose solution was changed daily during the next 4 days. The weight of animals was measured before and after the test, volume of water and sucrose solution was estimated daily. Sucrose preference in % of drunken fluid during baseline and testing was calculated [14,15,19].

Supplementary Methods

Mouse Resting State fMRI

FMRI measurements were conducted on a 4.7 T small animal MRT (Bruker Biospec, Bruker BioSpin MRI GmbH, Ettlingen, Germany). The MRT was equipped with a 200 mT/m gradient system, an actively decoupled RF-coil system for excitation and was operated by ParaVision software (V. 5.1.1, Bruker BioSpin MRI GmbH, Ettlingen, Germany). Animals were anesthetized for 4 min in 5% isoflurane in medical air. After induction of anaesthesia, an i.p.-catheter was set up in the right lower quadrant of the abdomen using a 27G-venipuncture set with wings, fixed securely with tape. The mouse was mounted on a specifically developed acrylic cradle (Bruker BioSpin MRI GmbH, Ettlingen, Germany), and the syringe was fixed at the side of the scanner wall to ensure injection without moving the cradle. An integrated water heating system helped to keep the body temperature constant at 37 °C. The head of the animal was firmly fixed by the front teeth in a nose-mouth mask that prevented head movement. Low concentrations (0.7-1.5 %) of isoflurane and oxygen/air were supplied for constant anaesthesia during the whole imaging process. If necessary, anaesthesia was adjusted during the measurement to keep the breathing rate between 90 and 120 breaths/min, known to result in minimal head movement but also best BOLD-contrast. The eyes of the animal were covered with an eye and nose ointment (Bepanthen, Bayer Vital GmbH, Leverkusen, Germany) in order to prevent exsiccation damage. For MR signal detection and good signal-to-noise ratio, a 3 cm 4-channel array head coil (Bruker BioSpin MRI GmbH, Ettlingen, Germany) was used. To verify the correct positioning of the animal inside the scanner, initial scout images as well as typical adjustments to correct field inhomogeneities (shimming) were performed prior to the fMRI measurements. A fast gradient Echo Planar Imaging sequence (TR=100 ms; TEeff=25.3 ms; FOV=15 mm * 15 mm; 1 slice; slice thickness 0.5 mm; matrix 64*64 voxel) was performed with 300 repetitions. Played as a video, it allowed detecting head movement of the animal. If so, the animal was remounted, and the positioning process restarted. A fast whole-brain coronal T2-weighted rapid acquisition relaxation enhanced sequence (RARE; TR=2000 ms; TEeff=56 ms; k-space averaging 4; RAREFactor = 8; FOV = 15 mm * 15 mm; 22 slices; slice thickness 0.5 mm; matrix 128*128 voxel) was recorded. It was used to position 22 axial slices covering the brain from Bregma -2.06 mm to 1.42 mm using anatomical landmarks (distal end of the lateral ventricle). This volume was scanned once using axial RARE (TR=2649 ms; TEeff=56 ms; k-space averaging 4; RAREFactor=8; FOV=15 mm * 15 mm; 22 slices; slice thickness 0.5 mm; matrix 128*128 voxel), to verify correct positioning. Afterwards, one volume of 22 axial slices with an in-plane resolution of 0.234 mm * 0.234 mm was acquired using gradient Echo Planar Imaging (TR=2000 ms; TEef=25.3 ms, FOV=15 mm * 15 mm; slice thickness 0.5 mm; matrix 64*64 voxel) to reassure good image quality. If inevitable, the FastmapScout macro implemented in ParaVision 5.1 was used to correct local field inhomogeneities.

Supplementary Methods

Mouse Resting State fMRI

Pre-processing, after discarding the first 2 volumes of the datasets avoiding MR saturation effects, comprised slice scan-time correction (ascending interleaved, interpolation method cubic spline) and motion correction to eliminate the minimal mouse head movement (registration to first brain volume; trilinear detection and sinc interpolation) performed in Brainvoyager QX (Brain Innovation, Maastricht, Netherlands; V 2.8.2.2523). Further analysis was performed in MagnAn (BioCom GbR, Uttenreuth in IDL, Harris Geospatial Solutions). First, a low pass filter at 0.1 Hz was applied to remove physiological noise. Despite careful positioning of the animals within the scanner, subject position is still slightly variable. To allow group analysis, the mice were registered to an anatomical reference using affine grey value-based volume registration with 6 degrees of freedom (translation x-, y-, and z-axis, rotation z-axis, isotropic scale in x-y-axis) followed by slice-wise translation and isotropic scaling. An in-house modified digital 3D Paxinos mouse brain atlas was used to identify 206 separate brain regions. Individual brain masks were used to confine the analysis to voxels inside the brain. A seed region, containing at most 5 voxels, was placed at the center of mass of each brain region for multi-seed region analysis. The Pearson correlation coefficient r was calculated between the average time course of each seed region and those of all voxels within the brain. This approach yielded one correlation volume per seed region for each animal and each timeframe. Significant correlations were determined for each correlation volume using false discovery rate (q=0.05). After converting r-values to Fisher-z-values, the correlation values of significant voxels belonging to the same brain region were averaged. For each seed, this yielded one row in an asymmetric connectivity matrix, summarizing the correlation between all seed regions to all brain structures. To ensure topological comparability, the matrices were normalized to the same density of connections. Per matrix, only the 7% strongest connections (i.e., highest r-values) were used for statistical comparison. Network based statistics (NBS) were applied to detect significant differences in RSpre between fro and WT mice: first, a homoscedastic t-test was calculated voxel-wise between the group matrices of fro and wt. The largest component of interconnected nodes at a significance level of a=0.05 was determined and its number of connections was kept. Next, the animals were assigned randomly to two groups, and t-tests were recalculated (1000 permutations). The number of comparisons yielding larger components than the original, primary t-test (i.e. more kept connections) divided by 1000 (number of permutations), gave a new p-value corrected for multiple comparison. Only if this p-value was below 0.05, the whole component was accepted as significant between groups.

Supplementary Methods

Osteocalcin treatment in mice

Mini pumps were filled with the solution of osteocalcin in 1x PBS and implanted into the lower back of animals under isoflurane anaesthesia (Isoflurane; Baxter Germany GmbH; 5% induction, 2% maintenance). Control animals were implanted with vehicle-containing pumps. After the implantation, the animals were single housed with 2 water bottles and left for recovery for 6 days. Thereafter the animals were tested in a battery of behavioural tests including the open field, elevated plus maze, and novelty supressed feeding as described above. Mice were returned to their home cages at the end of each test and allowed to recover for at least 1 day before further testing. Behaviors for all tests were recorded on videotape for subsequent scoring. After the last behavioral test, the animals were exposed to alcohol on the model of 2-bottle free-choice paradigm as mentioned above. The concentration of alcohol was gradually increased from 2 to 16 vol.% every 2 days, the concentration of 16 vol.% was kept for 5 days. Then the animals were sacrificed by cervical dislocation [71,72].

Supplementary Methods

RNA-Seq analysis in mouse osteoblasts

Primary osteoblasts were isolated from calvariae of 4-6 day old neonatal wild type and knock out mice. Calvariae were digested in 1 ml α-MEM, containing 0,1 % collagenase A and 0,2 % dispase II. The cells were suspended in osteoblast culture medium (αMEM + GlutaMAX, 10 % FCS, 1 % PS, 1 % L-Glutamine). To induce osteogenic differentiation, we seeded 2x104 calvarial cells on a 6-well plate. When the cells became confluent, osteoblast culture medium was replaced by osteogenic induction medium (αMEM + GlutaMAX, 10 % FCS, 1 % PS, 50 µg/ml L-ascorbic acid 2-phosphate, 10 mM β-Glycerolphosphate). After 21 days RNA was isolated from the osteoblasts using the RNeasy Micro Kit (Qiagen) according to manufacturers' instructions. Isolated RNA was dissolved in RNAse-free water and stored at -80 °C.

The quality of isolated RNA samples was determined using the Agilent 2100 Bioanalyzer equipped with an Agilent RNA 6000 Nano kit and related software (Agilent, Santa Clara, CA). Sequencing libraries were generated from 1 µg high quality RNA using the TruSeq Stranded mRNA Kit (Illumina, San Diego, U.S.A.) according to manufacturer's instructions. Libraries were sequenced on a HiSeq 2500 platform (Illumina, San Diego, U.S.A.) as 101 bp single-end reads to a depth of at least 40 million reads. Reads were converted to FASTQ format while masking Illumina adapter sequences (bcl2fastq v2.17.1.4, Illumina, San Diego, U.S.A). The data was additionally filtered to remove reads with more than 50% masked bases (fqtrim v0.9.5). The filtered reads were mapped to the Mus musculus reference genome GRCm38 (mm10), Ensembl gene annotation 93, using the splice-aware aligner STAR v2.6.0c. Mapped reads were quantified as reads per gene while excluding exons shared between more than one gene (subread featureCounts v1.5.1). Based on this quantification, differentially expressed genes were determined using a negative binomial model as implemented in DESeq2 v1.24.0 under R v3.6.0. Results from significance tests were corrected for multiple testing (Benjamini-Hochberg).

Network analysis was performed with the R package, weighted gene co-expression networks (WGCNA) on normalized and transformed data obtained from the RNA-seq. Genes with excessive zero count (80% of total sample) were removed and genes with variance above the median variance were retained for the network analysis. We used a robust correlation measure bicor to create a correlation matrix containing all pair-wise correlations between all genes across all samples.

Supplementary Methods

Mouse structural MRI and bone CT

Free-choice consumption Alcohol drinking was established in naïve female Fro and WT mice (n=7-8/group) using a two-bottle free-choice drinking paradigm as described above (Müller et al., 2017). After 12 days drinking of 16 vol.% alcohol, animals were exposed to MR imaging. A small-animal MRI system (ClinScan 70/30, Bruker BioSpin MRI GmbH, Ettlingen, Germany) was used for image acquisition. Animals were anesthetized with isoflurane (1.5%) prior to the procedure. During the MRI scan an animal monitoring system was used for surveillance of heart and lung functions. The body core temperature was kept constant during the procedure at 37 °C using a heated pad. The volume and the signal intensity of the structures given above were calculated by measuring the respective regions of interest using , Chimaera's segmentation tool (Chimaera GmbH). The following T2 Turbo Spin Echo sequence was used: Time of acquisition 14:03 min, voxel size 0.078 x 0.078 x 0.4 mm, TE:44 ms, TR: 5660 ms, slice thickness 0.4 mm, 30 slices.

Using computed tomography (CT), bone density was determined in various osseous structures (skull, spine, femur, tibia, humerus, radius and ulna) by assessing the Hounsfield Units (HU) of the respective bone. For acquisition, a whole-body protocol was performed on a dedicated micro CT scanner (Inveon, Siemens) with the following parameter: Projections 180, settle time 1000 ms, pixel size 51 µm, voltage 80 kV, current 500 µA, exposure 1100 ms.

Supplementary Methods

Oxidative stress measurements

After 12 days drinking of 16 vol.% alcohol, animals were sacrificed and the brains were isolated. Frozen brain tissues were homogenized in a polyphosphate buffer solution (pH=7.5). Oxidative stress markers were determined using a commercial assay kits according to manufacture protocols. Superoxide dismutase (SOD) activity was measured using Superoxide Dismutase Assay Kit (706002; Cayman Chemical, USA), which utilizes a tetrazolium salt for detection of superoxide radicals generated by xanthine oxidase and hypoxanthine. SOD activity was determined as the amount of enzyme ability that exhibit 50% dismutation of the superoxide radical and is expressed as [U] per ml of tissue homogenates [U/ml]. Catalase activity was measured using Catalase Assay Kit (707002; Cayman Chemical, USA), which utilizes the peroxidatic function of catalase for the determination of enzyme activity. The method is based on the reaction of the enzyme with methanol in the presence of an optimal concentration of H2O2. The formaldehyde produced is measured colorimetrically with 4-amino-3-hydrazino-5-mercapto-1,2,4-triazole. This chromogen specifically forms a bicyclic heterocycle with aldehydes, which upon oxidation changes from colorless to purple color. One unit is defined as the amount of enzyme that will cause the formation of 1 nmol of formaldehyde per minute at 25˚C [nmol/min/ml]. Lipid peroxidation was performed using Thiobarbaturic Acid Reactive Substances (TBARS) Assay Kit (700870; Cayman Chemical, USA). The measurement of TBARS is a well-established method for screening and monitoring lipid peroxidation. The malondialdehyde (MDA), a naturally occurring product of lipid peroxidation, and TBA under high temperature (90-100˚C) and acidic conditions formed the MDA-TBA adduct measured colorimetrically at 540 nm. The data are calculated using a standard curve of different MDA concentrations and presented as MDA [μM] [73,74].

Supplementary Methods

Electron microscopy of hippocampus slices

The effects of alcohol on the morphology of hippocampal synapses were evaluated in female fro and WT mice (n=3 animals/group). Alcohol drinking was established in naïve animals using a two-bottle free-choice drinking paradigm as described above. After 12 days drinking of 16 vol.% alcohol, fro mice and WT littermates were intracardially perfused with PBS and fixative (4% paraformaldehyde [PFA] and 2% glutaraldehyde in PBS). Brains were fixed in 4% PFA overnight and sectioned in 200-µm-thick slices. Hippocampal sections were postfixed in 1% osmium tetroxide (in 0.1 M cacodylate buffer), dehydrated in ethanol and embedded in Epon (Sigma-Aldrich, 45359-1EA-F). Serial ultrathin sections of the CA1 region were collected on pioloform-coated, single-hole grids, and stained with uranyl acetate and lead citrate. The sections were examined with a transmission electron microscope (JEM1010, jeol, Akishima, Tokyo, Japan). Synapses from CA1 were identified by position and sampled randomly and photographed at a 20000x magnification with a CMOS 4 k TemCam-F416 camera (TVIPS, Gauting, Germany). Images were quantified using ImageJ software (National Institutes of Health, Bethesda, MD, USA), and SV density was calculated as the number of vesicles per m2 within 10 nm of the presynaptic membrane and not more than 300 nm from the active zone border.

Supplementary Methods

Neurogenesis measurement in mice

Primary antibodies from rabbit anti-MCM2 (1:500, Cell Signaling Technologies), from mouse anti-Nestin (1:500; Millipore) and from guinea pig anti-DCX (1:250; Millipore) and secondary anti-rabbit 488 (1:1000; Jackson ImmunoResearch), anti-mouse Cy3 (1:1000; Jackson ImmunoResearch) and anti- guinea pig Cy3 (1:1000; Jackson ImmunoResearch) were used for staining on free-floating 40 µm brain coronal sections. Sections were blocked in TBS with 10% normal donkey serum and 0.3% Triton-X-100 at RT for 1 h. Primary antibodies was applied in the same solution for 72 h at 4°C. Upon three-times rinsing with TBS and 1 h blocking in TBS with 3% FCS and 0.3% Triton-X-100 secondary antibody in the same solution were applied at 4°C overnight. After extensive washing in TBS, nuclei were stained with DAPI (Sigma), washed again with TBS and mounted on coverslips with Prolong Gold (Thermo Fisher Scientific Invitrogen). Images were acquired using 20x objective lens on a fluorescence microscope equipped with SIM (Apotome.2, Zeiss, Jena, Germany). Acquisition, processing and analysis were performed using Zen series software (Zeiss, Jena, Germany). Stage-specific marker expression (Nestin, MCM2, DCX) was assessed in the coronal sections containing medial dentate gyrus. Nestin and MCM2 were quantified in 3D volumes allowing morphological identification of radial-glia like stem cells, while DCX positive cells were counted on 2D images. 2 sections per animal and 4 animals per group were quantified in a blinded fashion.

Supplementary Methods

In-vivo microdialysis in mice

The experiment was performed on naïve female fro and WT mice (n=7-10/group). For cannula implantation, the mice were deeply anaesthetized with isoflurane (4-5% for initiation and 2-3% for maintenance, O_2 at 2 L/min) and 1% lidocaine, 0.1ml, (s.c.) for local anesthesia. Two guide cannulas (Microbiotech/se AB) were aimed at the DH (A: -2.1; L: ±1.2; V: -1.1) and the Nac (A: +1.2; L: ±1.6; V: -4.3; angle ±10º from midline) using coordinates relative to bregma [75]. The cannulas were fixed using two anchor screws (stainless steel, d=1.4mm) and dental cement. After the surgery 0.01 ml Rimadyl (5 mg/kg Carprofen) per 10 g body weight and 0.1 ml physiological saline were given s.c. for analgesia. Animals were kept warm during and after the surgery, then returned to their home cages and monitored daily. The animals were given at least 5 days between the surgery and dialysis and were handled daily for 5 min/mouse [14,32].

On the day of the experiment, microdialysis probes with membrane length of 1 mm were inserted into the guide cannulae under a short (1-2 min) isoflurane narcosis (O_2 at 2 L/min, Isoflurane at 3-5%). After probe insertion, each animal was placed into a Plexiglas chamber (21 x 21 x 30 cm) with water ad libitum and room temperature 19-22 ºC. The probes were connected to a microinfusion pump (CMA 400, Carnegie) and were perfused with artificial cerebrospinal fluid (Carl Roth GmbH) at room temperature and the flow rate of 1.5 µl/min. The perfusion was allowed to stabilize for two hours until a stable baseline was achieved, and then the samples started being collected. They were collected every 20 min into vials with 2.73 µl of antioxidant (0.1 M perchloric acid) and 500 pg dihydroxybenzylamine as internal standard (Sigma/ Carl Roth GmbH). First three samples were used to measure baseline quantities of the neurotransmitters DA, and 5-HT [71]. After 1 h, three samples were collected as a baseline for the alcohol stimulus followed by an i.p. injection of alcohol (2 g/kg, v_{inj}=10 ml/kg) and further six samples were collected. Once the microdialysis experiments were completed, the animals were sacrificed by cervical dislocation. The brains were isolated, and the localization of the probe was verified. Only samples from the animals with probes placed within the DH and Nac were considered for further analysis.

All samples were analyzed using HPLC with electrochemical detection to measure DA and 5-HT levels. The column was an ET 125/2, Nucleosil 120-5, C-18 reversed phase column (Macherey & Nagel). The mobile phase consisted of 75 mM NaH_2PO_4, 4 mM KCl, 20 µM EDTA, 1.5 mM sodium dodecylsulfate, 100 µl/L diethylamine, 12% alcohol and 12 % acetonitrile adjusted to pH 6.0 using phosphoric acid (Carl Roth GmbH). The electrochemical detector (Intro) was set at 500 mV vs. an ISAAC reference electrode (Antec) at 30°C. This setup allows simultaneous measurement of DA, 5-HT, and NE [32,71].

Supplementary Methods

Monoamine receptor mRNA expression in the mouse brain

After 12 days drinking of 16 vol.% alcohol, animals were sacrificed and ventral striatum was isolated and immediately frozen on dry ice. The samples were stored at -80°C until further analysis. Total RNA was isolated from pieces of mouse brain tissue (<30 mg) using a TissueLyser LT bead mill (Qiagen, Hilden, Germany) and peqGOLD Trifast reagent (Peqlab, Erlangen, Germany) according to the manufacturers’ instructions, followed by RNA purification using the RNeasy Mini Kit (Qiagen) according to manufacturers' instructions. cDNA was generated from 1 µg RNA using the SuperScript VILO cDNA Synthesis Kit (ThermoFisher) according to manufacturers' instructions. Quantitative real-time PCR was performed using a LightCycler 480 real-time PCR system (Roche, Germany) in taqman format. In detail, qPCR reactions contained 5 µl TaqMan FastStart Advanced Master Mix (ThermoFisher), 0.5 µl of FAM/VIC 20x taqman probe, 2 µl RNA-free water, and 2.5 µl diluted cDNA (corresponding to 12.5 ng RNA) in a total volume of 10 µl. Temperature profile used was: 35 cycles of amplification (95°C for 30 s, 95°C for 15 s, 60°C for 60 s) followed by 40°C for 10 s. Threshold cycles (Ct) were determined with the second derivative maximum method and relative mRNA expression levels were calculated using the LightCycler 480 software (release 1.5.0). The following TaqMan probes were used for the reaction: SERT, Assay ID Mm00439391_m1; 5-HT1a: Assay ID Mm00434106_s1; 5-HT2a: Assay ID Mm00555764_m1; 5-HT2c: Assay ID Mm00434127_m1; 5-HT3a: Assay ID Mm00442874_m1; DAT, Assay ID Mm00438388_m1; D1: Assay ID Mm02620146_s1; D2: Assay ID Mm00438545_m1; as reference Gapdh was used (ThermoFisher).

Supplementary Methods

Serotonin uptake in mouse brain synaptosomal preparations

Mouse brains were rapidly removed and dissected. Individual brain regions, i.e. ventral and dorsal hippocampus and ventral striatum were homogenised in ice-cold homogenisation buffer (0.32 M sucrose, 5 mM Tris pH 7.4 and 1 mM EDTA buffer) using a Dounce homogeniser with a loose-fitted pestle. Homogenates were transferred to centrifuge tubes and large cell debris were removed by centrifugation at 1,300 g for 5 min at 4 °C. Supernatants were transferred to new tubes and centrifuged at 17,000 g at 4 °C for 10 min. The resulting P2 pellet was washed once in homogenisation buffer and centrifuged as before, aliquots for protein assay (BCA) were collected. The remaining crude synaptosomes were resuspended in ice-cold transport buffer (TB, 10 mM HEPES, 150 mM NaCl, 2 mM KCl, 1 mM CaCl2, 1 mM MgCl2, 10mM glucose) and diluted. Aliquots of synaptosomes (25-50µg/well) were pre-incubated at 37ºC in 200µL TB transport buffer containing 100mU/mL bacterial sphingomyelinase (BSM) in 24-well plates for 60 minutes. [3H]-5-HT (PerkinElmer) was then added to each well to give a final concentration of 20nM. The incubation was terminated after 10 min by addition of ice-cold transport buffer containing 1µM escitalopram and filtration onto a Microbeta 1450 Filtermat A using an Inotech Harvester. Filters were rapidly washed three times with transport buffer and dried overnight. Dried filters were immersed in scintillation fluid and counted by scintillation spectrometry using a Wallac Microbeta counter. Counts for SERT specific [3H]-5-HT uptake were determined by subtracting counts from parallel assays in the presence of 1µM escitalopram.

Supplementary Methods

Lipidomics

Lipidomics profiling was performed using Ultra Performance Liquid Chromatography-Tandem Mass Spectrometry (UPLC-MS) at the Biomarkers Core Laboratory, at Columbia University Irving Institute for Clinical and Translational Research, New York City, NY, USA. Lipid extracts were prepared from flash frozen samples using a modified Bligh and Dyer method [77], spiked with appropriate internal standards, and analysed on a platform comprising Agilent 1260 Infinity HPLC integrated to Agilent 6490A QQQ mass spectrometer controlled by Masshunter v 7.0 (Agilent Technologies, Santa Clara, CA). Glycerophospholipids and sphingolipids were separated with normal-phase HPLC as described before [78]. An Agilent Zorbax Rx-Sil column (2.1 x 100 mm, 1.8 µm) maintained at 25°C was used under the following conditions: mobile phase A (chloroform: methanol: ammonium hydroxide, 89.9:10:0.1, v/v) and mobile phase B (chloroform: methanol: water: ammonium hydroxide, 55:39:5.9:0.1, v/v); 95% A for 2 min, decreased linearly to 30% A over 18 min and further decreased to 25% A over 3 min, before returning to 95% over 2 min and held for 6 min. Separation of sterols and glycerolipids was carried out on a reverse phase Agilent Zorbax Eclipse XDB-C18 column (4.6 x 100 mm, 3.5 um) using an isocratic mobile phase, chloroform, methanol, 0.1 M ammonium acetate (25:25:1) at a flow rate of 300 µl/min. Quantification of lipid species was conducted using multiple reaction monitoring (MRM) transitions [78,79,80], under both positive and negative ionization modes in conjunction with referencing of appropriate internal standards: PA 14:0/14:0, PC 14:0/14:0, PE 14:0/14:0, PG 15:0/15:0, PI 17:0/20:4, PS 14:0/14:0, BMP 14:0/14:0, APG 14:0/14:0, LPC 17:0, LPE 14:0, LPI 13:0, Cer d18:1/17:0, SM d18:1/12:0, dhSM d18:0/12:0, GalCer d18:1/12:0, GluCer d18:1/12:0, LacCer d18:1/12:0, D7-cholesterol, CE 17:0, MG 17:0, 4ME 16:0 diether DG, D5-TG 16:0/18:0/16:0 (Avanti Polar Lipids, Alabaster, AL). Lipid levels for each sample were calculated by summing up the total number of moles of all lipid species measured by all three LC-MS methodologies, and then normalizing that total to mol %. The final data are presented as mean mol % with error bars showing mean ± s.d.

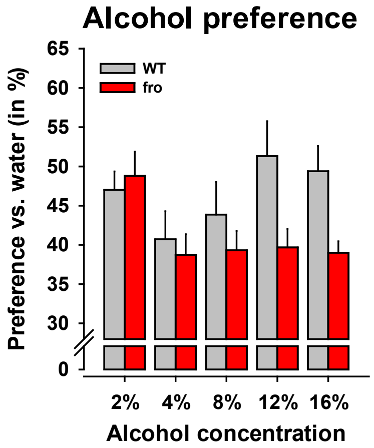

Figure S1 Neutral sphingomyelinase determines alcohol consumption in mice. Mice with a heterozygous neutral sphingomyelinase-2 (NSM) knock out mice (fro mice) show reduced alcohol preference in a two-bottle free choice paradigm compared to wild type (WT) controls.

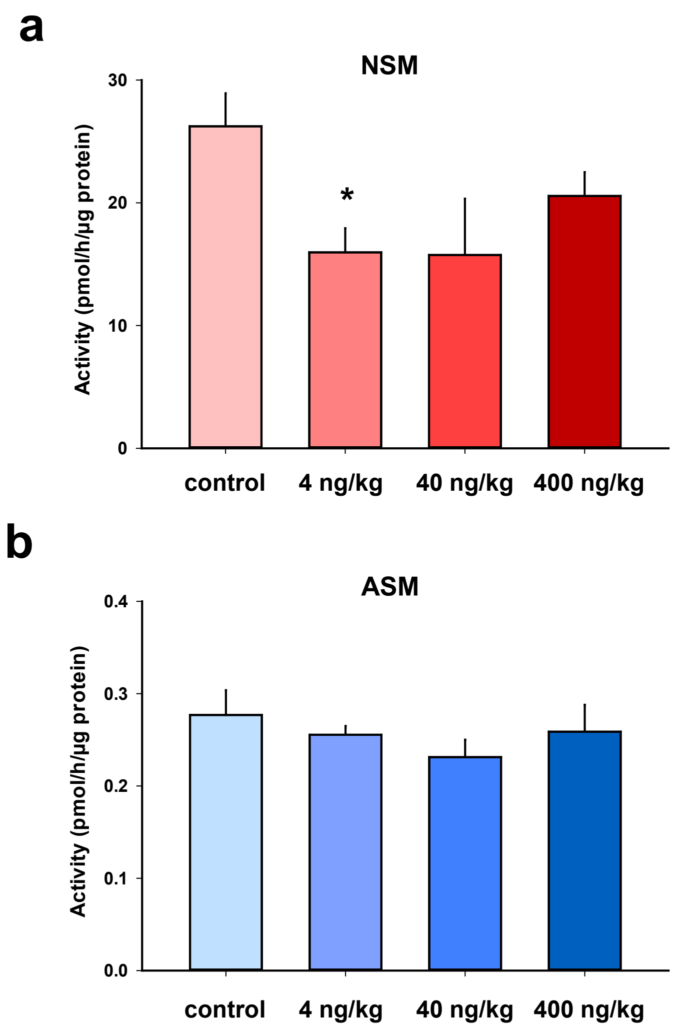

Figure S2 Effects of ES048 on sphingomyelinase activity in mouse hippocampal tissue after i.p. treatment. **a**, ES048 is an inhibitor of neutral sphingomyelinase (NSM) activity. **b**, It has no effects on acid sphingomyelinase (ASM) activity.

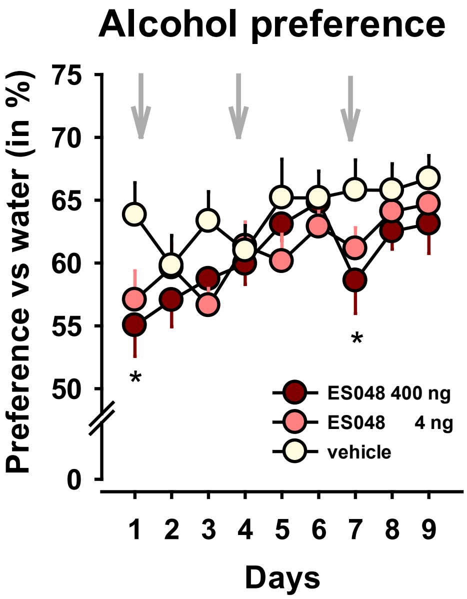

Figure S3 In mice, the NSM inhibitor ES048 (ng/kg; i.p.) attenuated alcohol preference in a previously established alcohol in an intermittent access 20 vol. % alcohol drinking test (grey arrows indicate treatment times; treatment *p<0.05 vs vehicle).

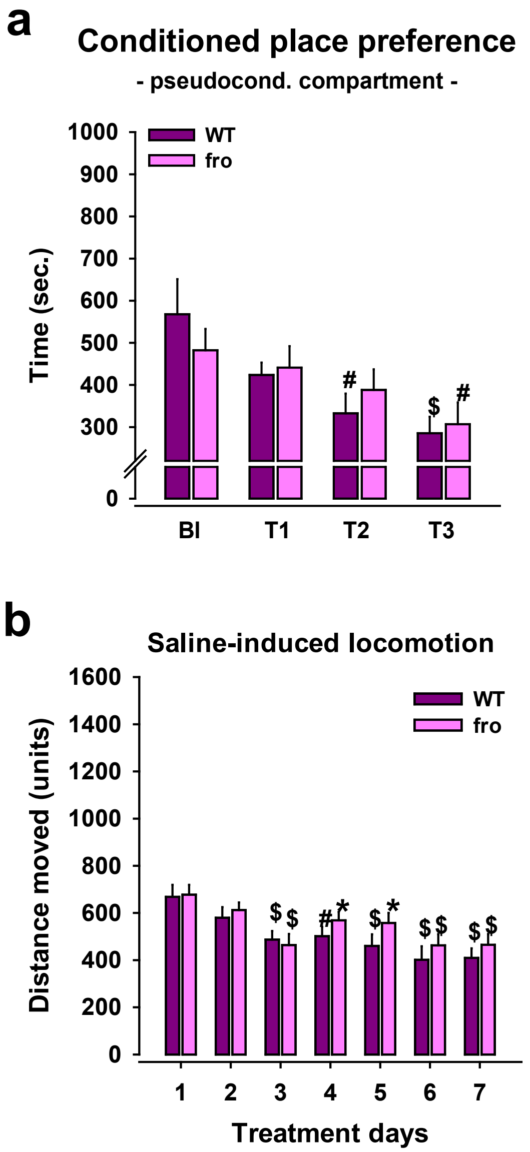

Figure S4 Neutral sphingomyelinase-2 controls establishment of the conditioned reinforcing effects of alcohol in mice. **a,** Decline of time spent in the pseudoconditioning compartment during establishment of an alcohol-induced conditioned place preference in heterozygous neutral sphingomyelinase-2 (NSM) knock out (fro) and wild type (WT) mice. **b**, Locomotor activity during pseudoconditioning trials after saline (i.p.) treatment (Bl-baseline; T-test trial; $^{*}p<0.05$; $^{\#}p<0.01$; $^{\$}p<0.001$ vs Bl or day 1).

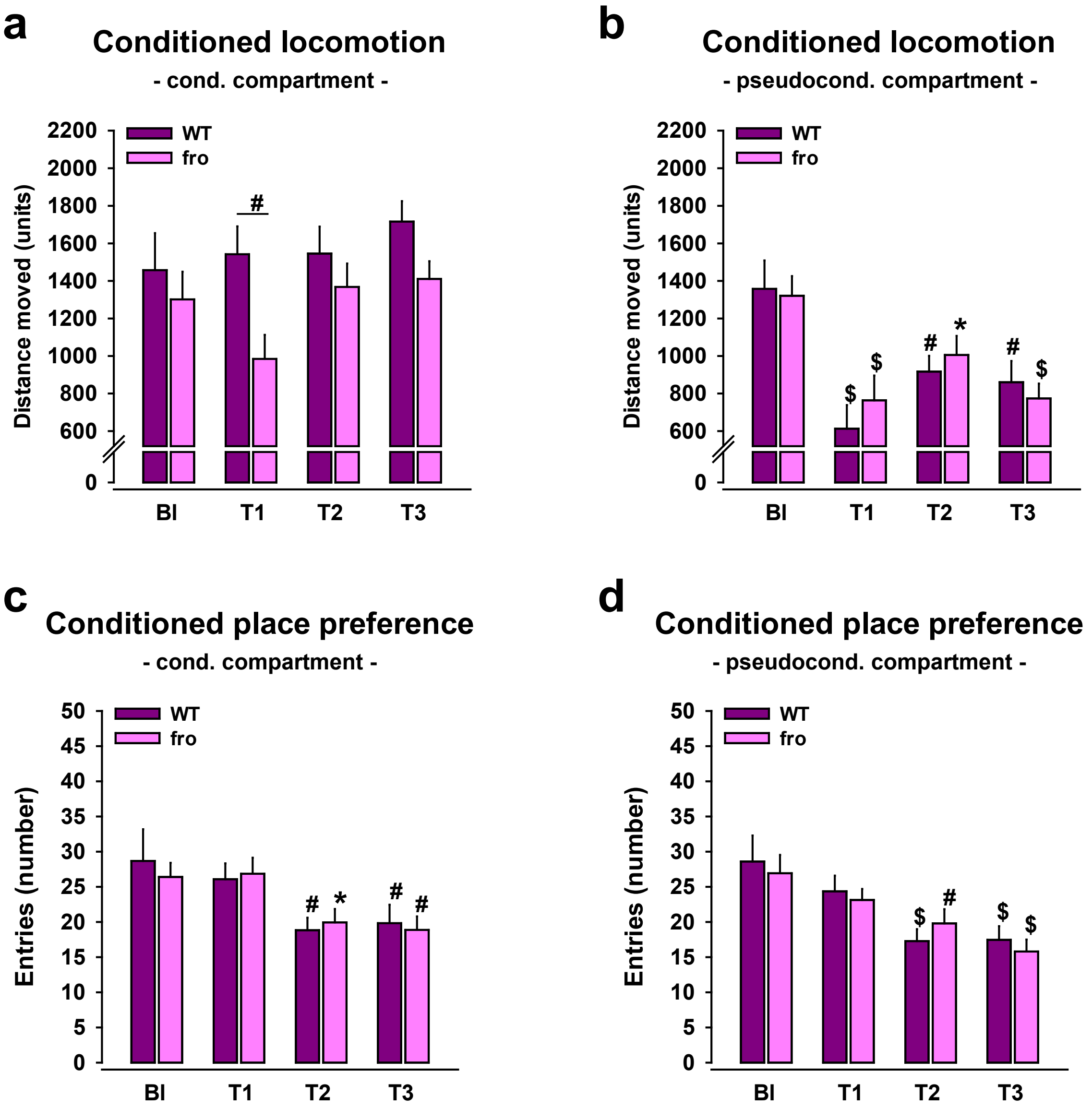

Figure S5 Neutral sphingomyelinase-2 controls establishment of the conditioned reinforcing effects of alcohol in mice. **a-b,** Locomotion during test trials in conditioned and pseudoconditioned compartment during establishment of an alcohol-induced conditioned place preference in heterozygous neutral sphingomyelinase-2 (NSM) knock out (fro) and wild type (WT) mice. **c-d**, Entries into compartments during CPP testing (Bl-baseline; T-test trial; $^{*}p<0.05$; $^{\#}p<0.01$; $^{\$}p<0.001$ vs Bl).

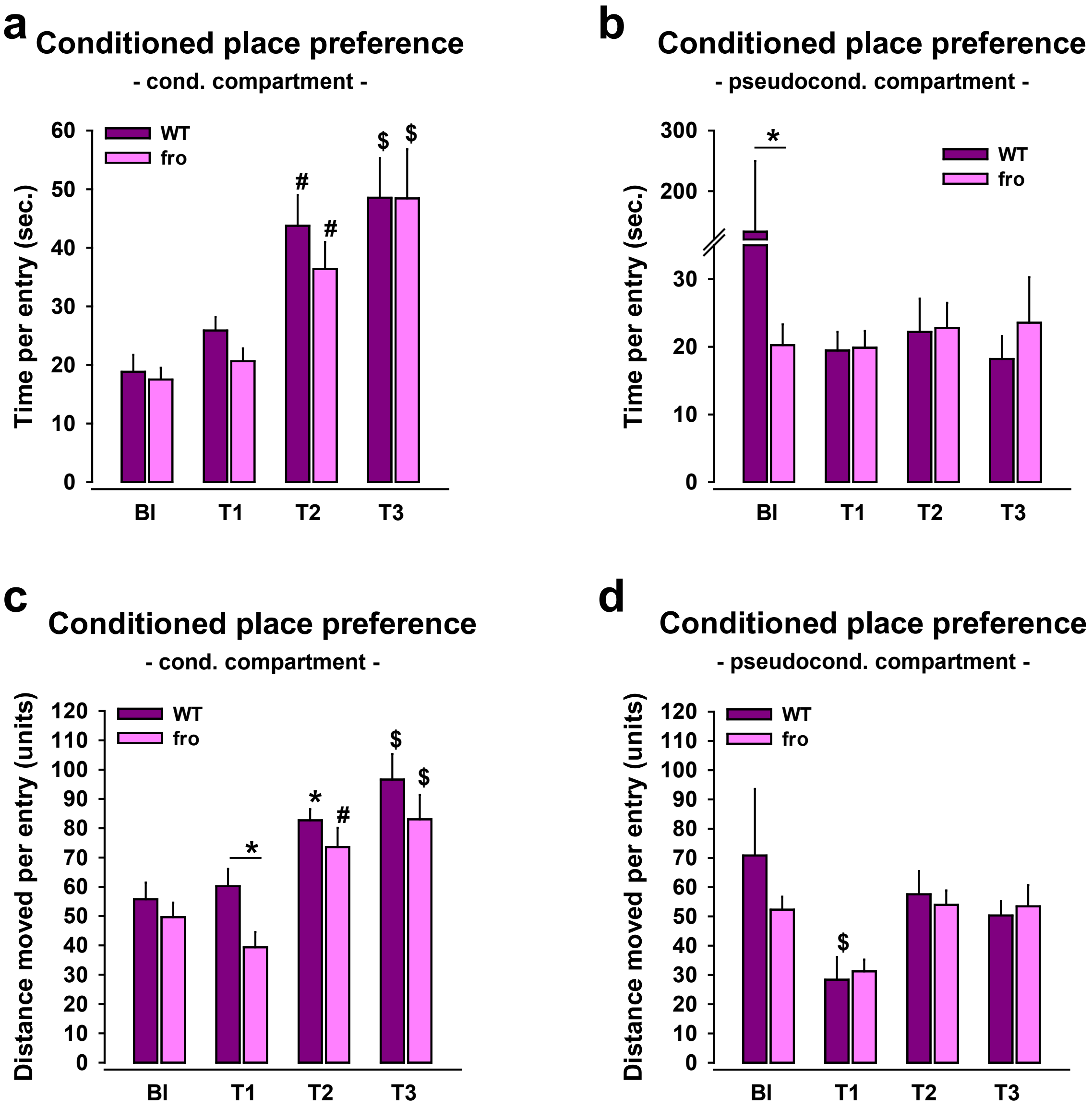

Figure S6 Neutral sphingomyelinase-2 controls establishment of the conditioned reinforcing effects of alcohol in mice. **a-b,** Time per entry during test trials in conditioned and pseudoconditioned compartment during establishment of an alcohol-induced conditioned place preference in heterozygous neutral sphingomyelinase-2 (NSM) knock out (fro) and wild type (WT) mice. **c-d**, Distance moved per entry into compartments during CPP testing (Bl-baseline; T-test trial $^{*}p<0.05$; $^{\#}p<0.01$; $^{\$}p<0.001$ vs Bl).

a

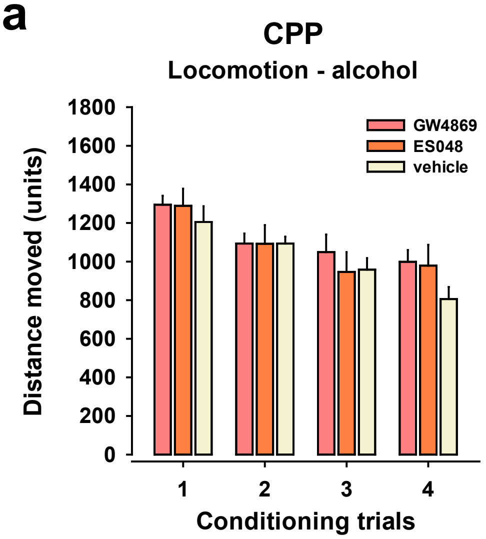

b

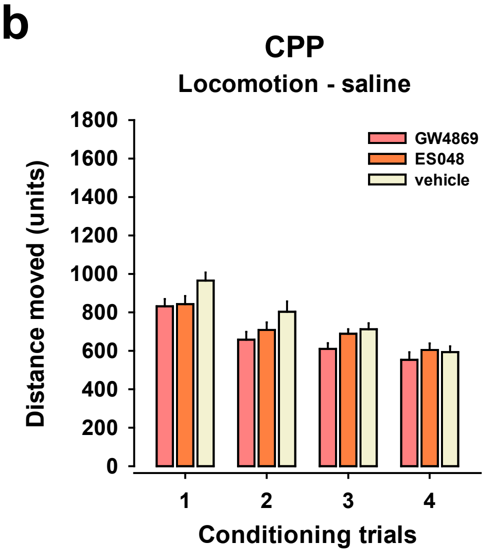

Figure S7 No role of neutral sphingomyelinase-2 in the expression of alcohol conditioned rewarding effects in mice **a-b**, Locomotion after alcohol or saline in the conditioning and pseudoconditioning trials of an alcohol alcohol-induced conditioned place preference (CPP) in heterozygous neutral sphingomyelinase-2 (NSM) knock out (fro) and wild type (WT) mice.

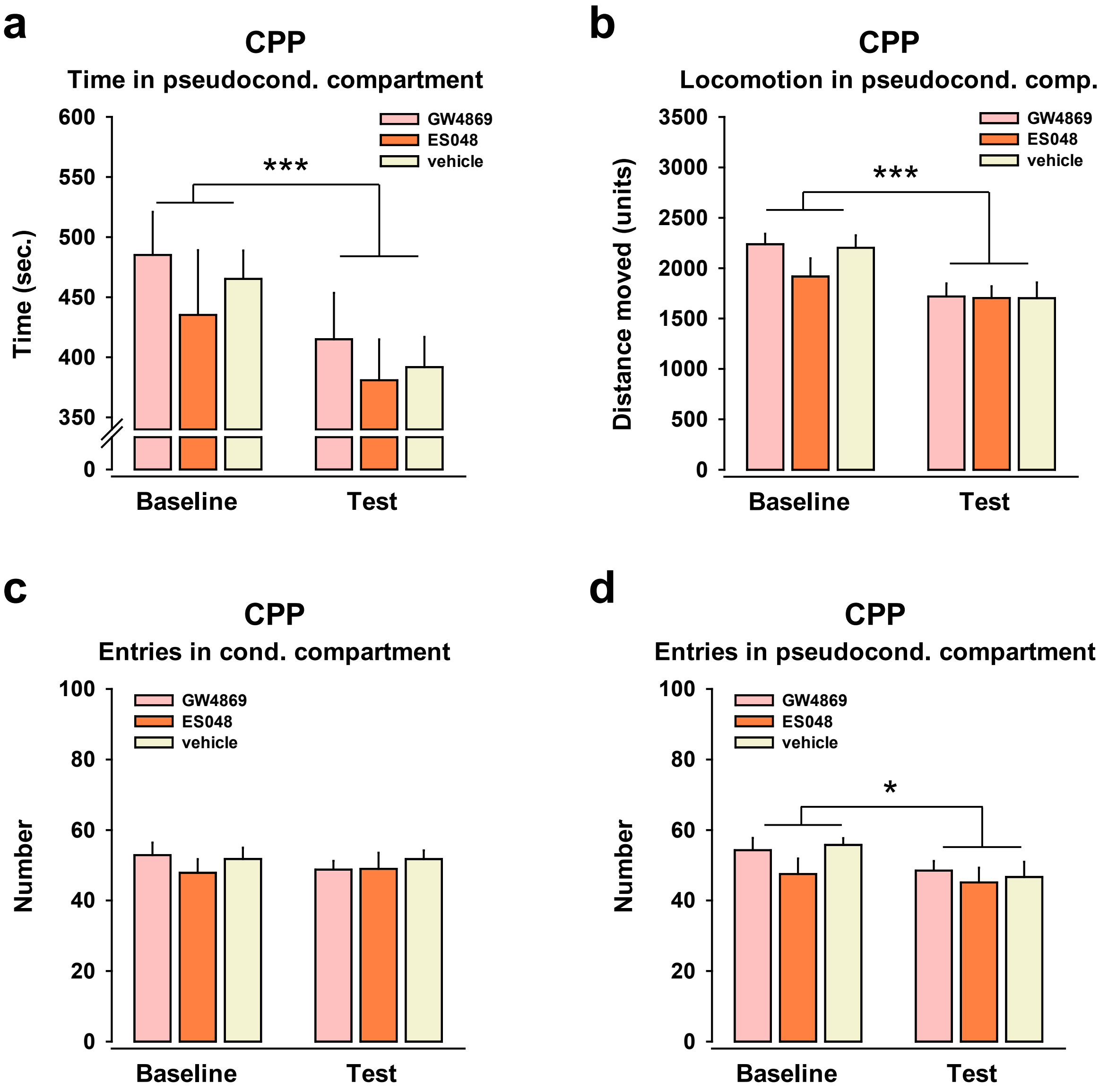

Figure S8 No role of neutral sphingomyelinase-2 in the expression of alcohol conditioned reinforcing effects in mice. **a,** Time in pseudoconditioning compartment during test trials of an alcohol-induced conditioned place preference. Mice were treated with NSM inhibitors before the test trial. **b**, Locomotion in pseudoconditioning compartment during test trials. **c-d**, Entries in compartments during test trials (*p<0.05; ***p<0.001; two-way ANOVA, effect of time).

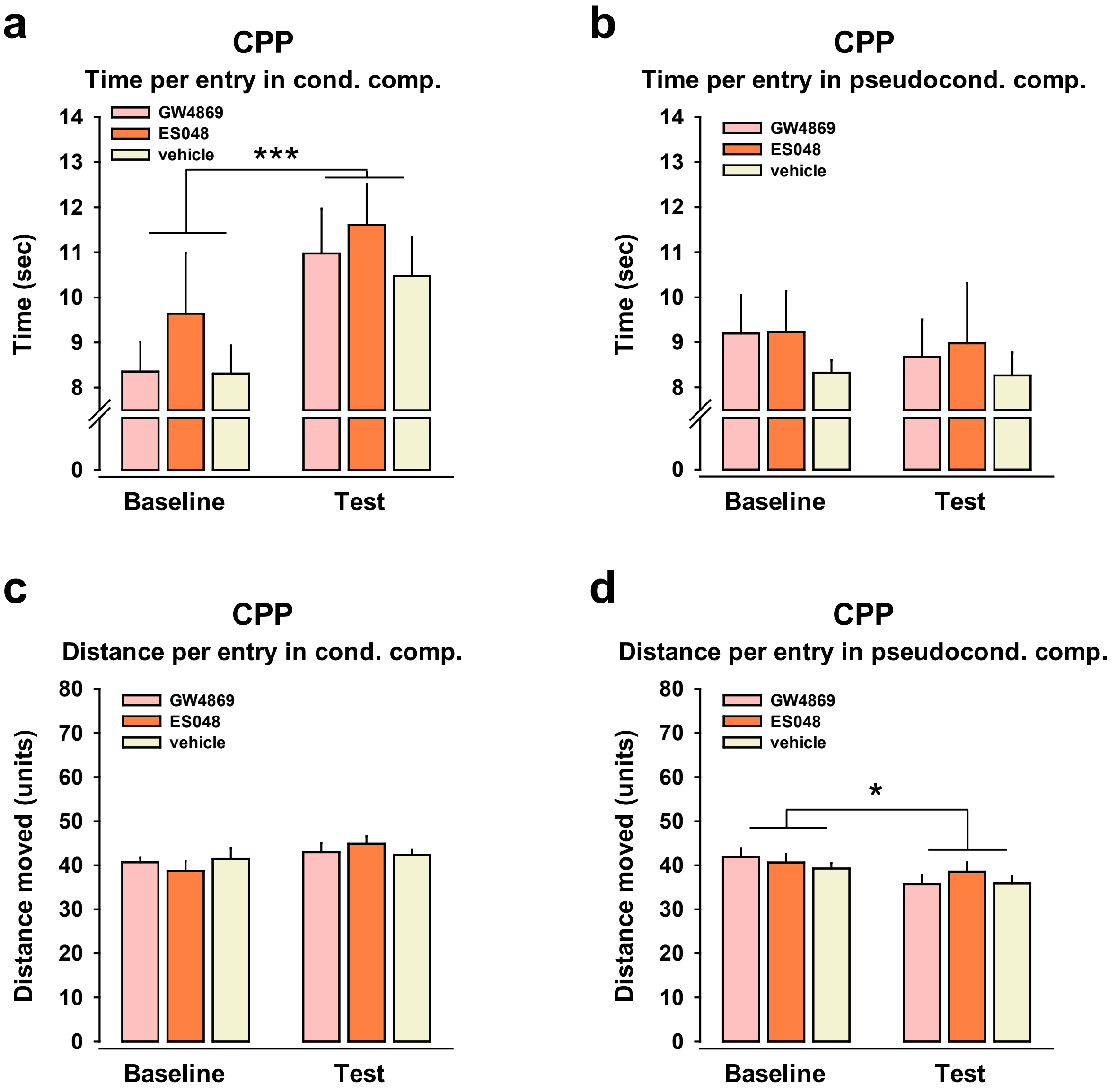

Figure S9 No role of neutral sphingomyelinase-2 in the expression of alcohol conditioned reinforcing effects in mice. **a-b,** Time per entry in conditioning and pseudoconditioning compartments during test trials of an alcohol-induced conditioned place preference. Mice were treated with NSM inhibitors before the test trial. **c-d**, Distance moved per entries in compartments during test trials (*p<0.05; ***p<0.001; two-way ANOVA, effect of time).

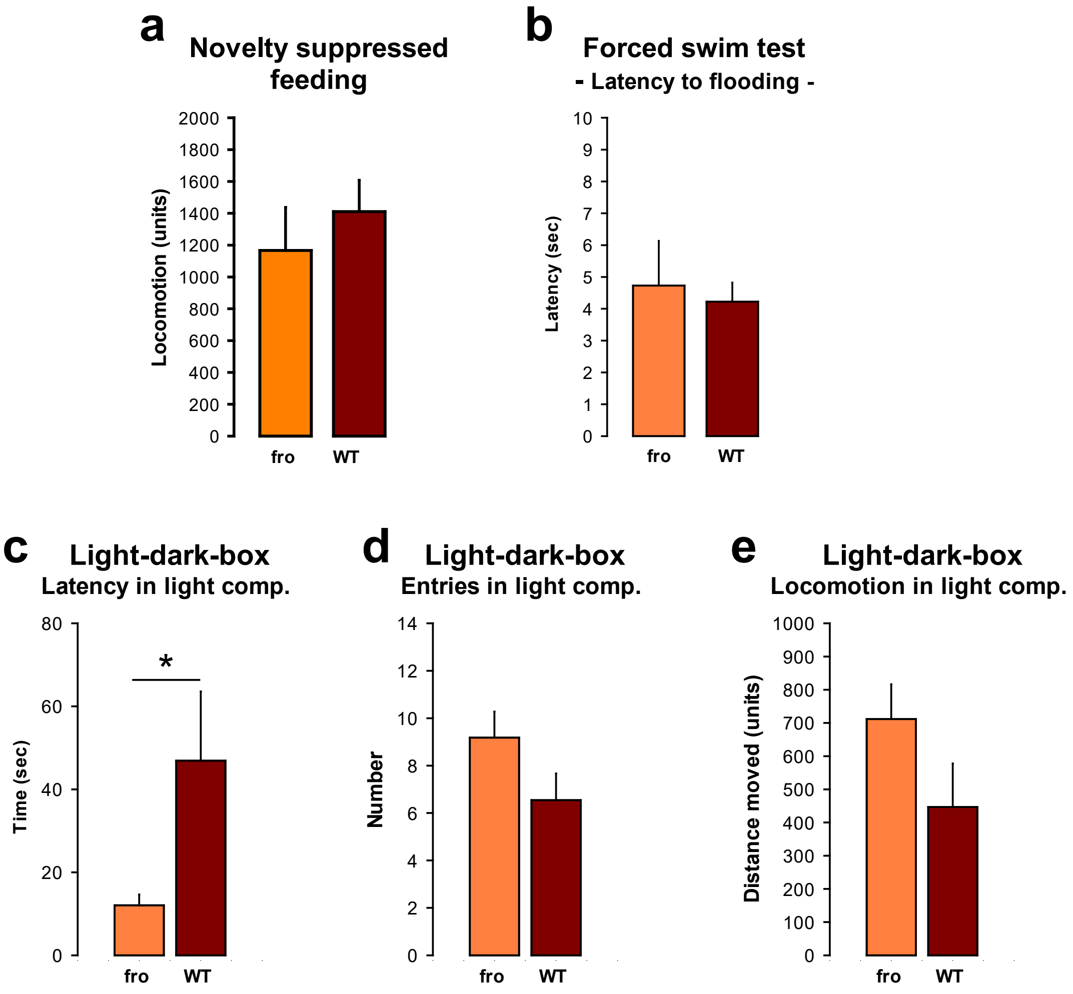

Figure S10 The role of neutral sphingomyelinase-2 (NSM) in emotional behaviour in mice tested in mice with a heterozygous NSM knock out (fro). **a,** Novelty suppressed feeding test. **b,** Forced swim test. **c-e,** Light-dark-box test (WT-wild type; *p<0.05).

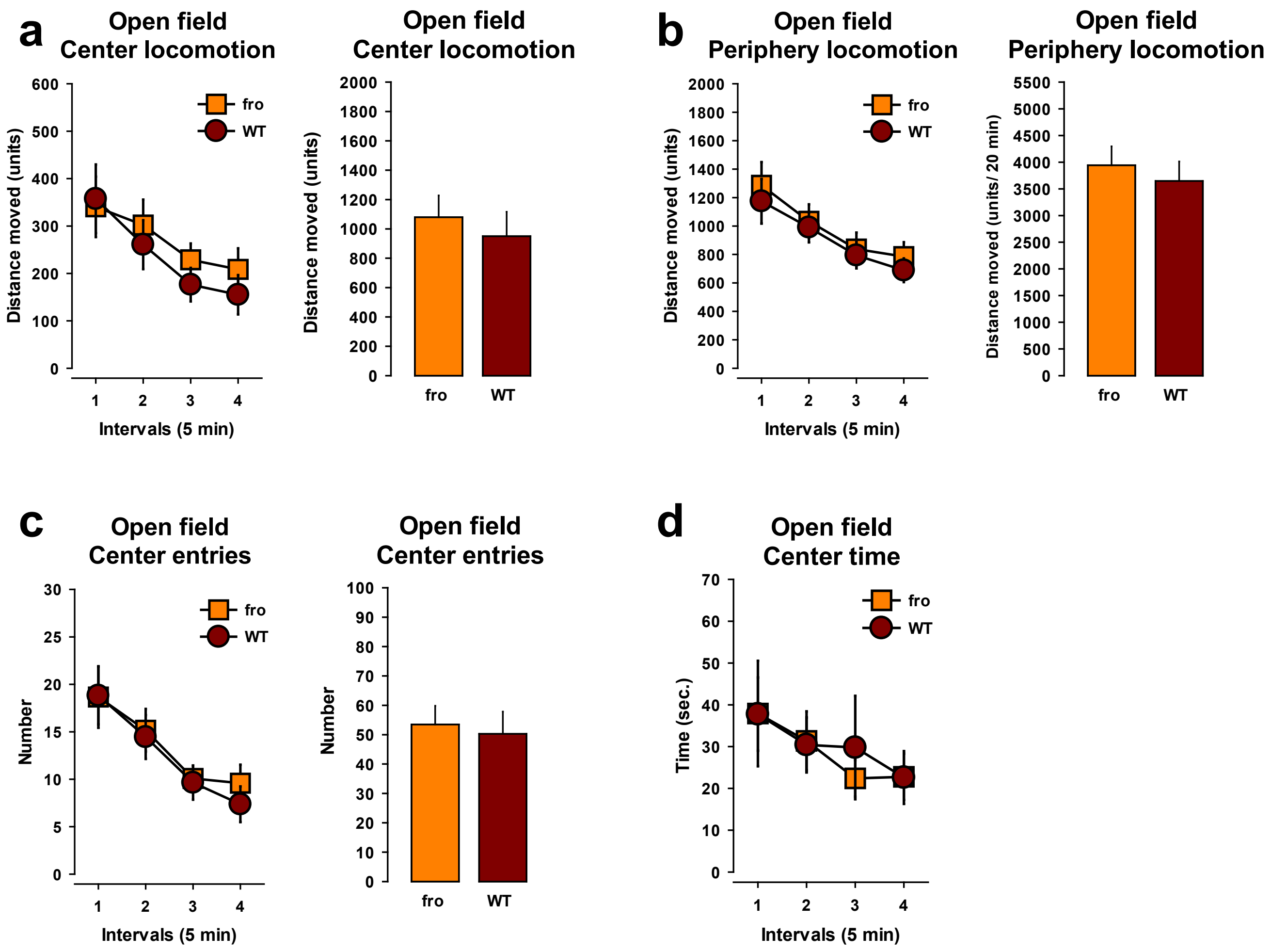

Figure S11 The role of neutral sphingomyelinase-2 (NSM) in emotional behaviour in mice tested in mice with a heterozygous NSM knock out (fro) in the open field test (left: single 5 min intervals, right: area under the curve; WT-wild type).

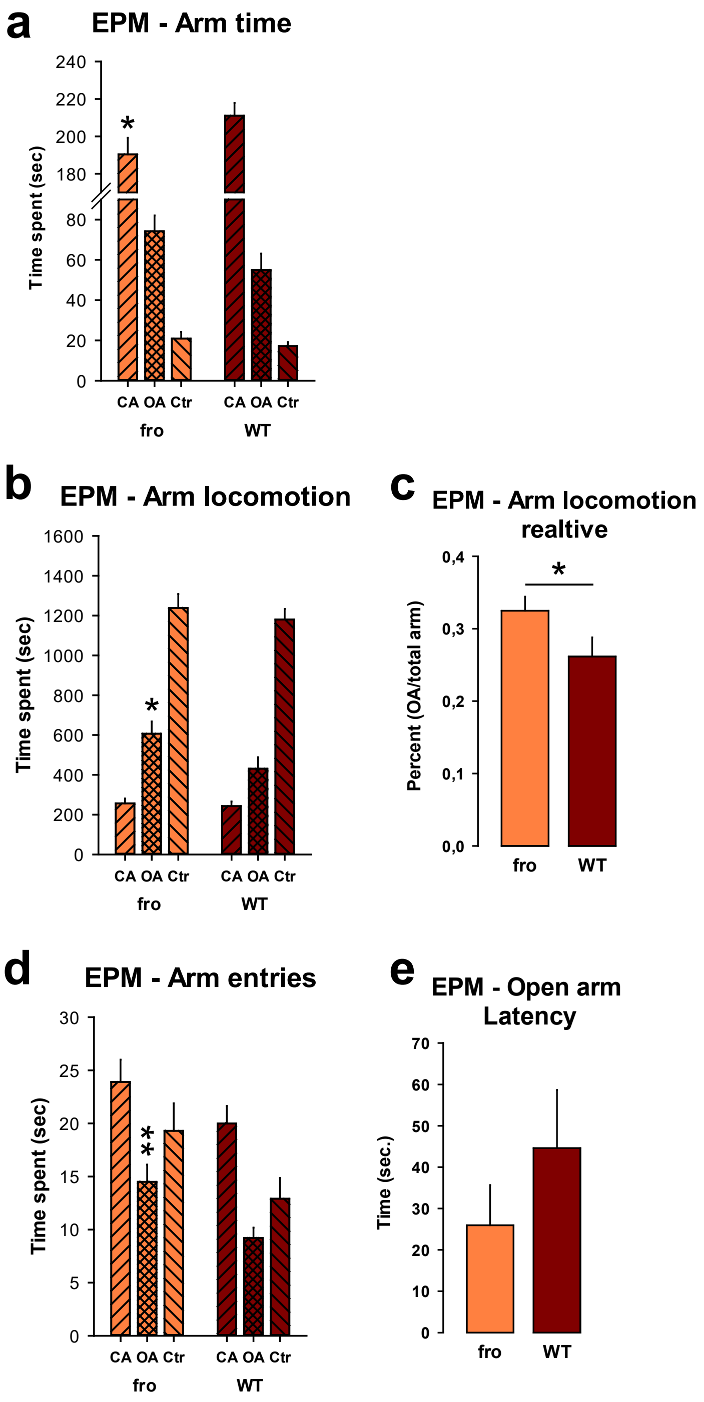

Figure S12 The role of neutral sphingomyelinase-2 (NSM) in emotional behaviour in mice tested in mice with a heterozygous NSM knock out (fro) in the elevated plus maze test (EPM) (CA – closed arm, OA – open arm, Ctr – center, WT-wild type; *p<0.05; **p<0.01 vs WT).

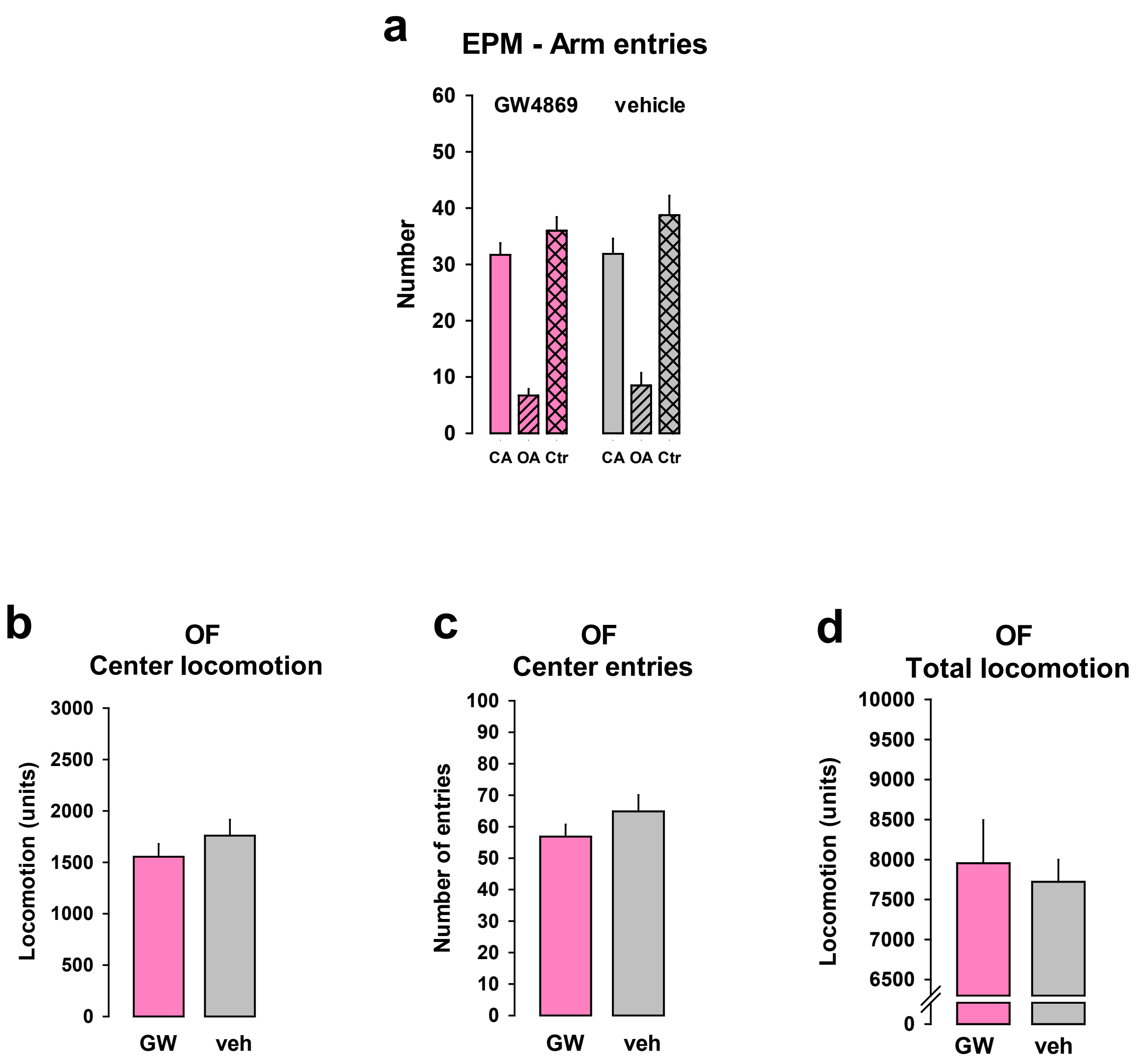

Figure S13 Effects of the neutral sphingomyelinase-2 (NSM) inhibitor GW4869 (GW; i.p.) on anxiety-related behaviour in mice in the **a**, elevated plus maze (EPM) and **b-d**, in the open field test (CA – closed arm, OA – open arm, Ctr – center, WT-wild type).

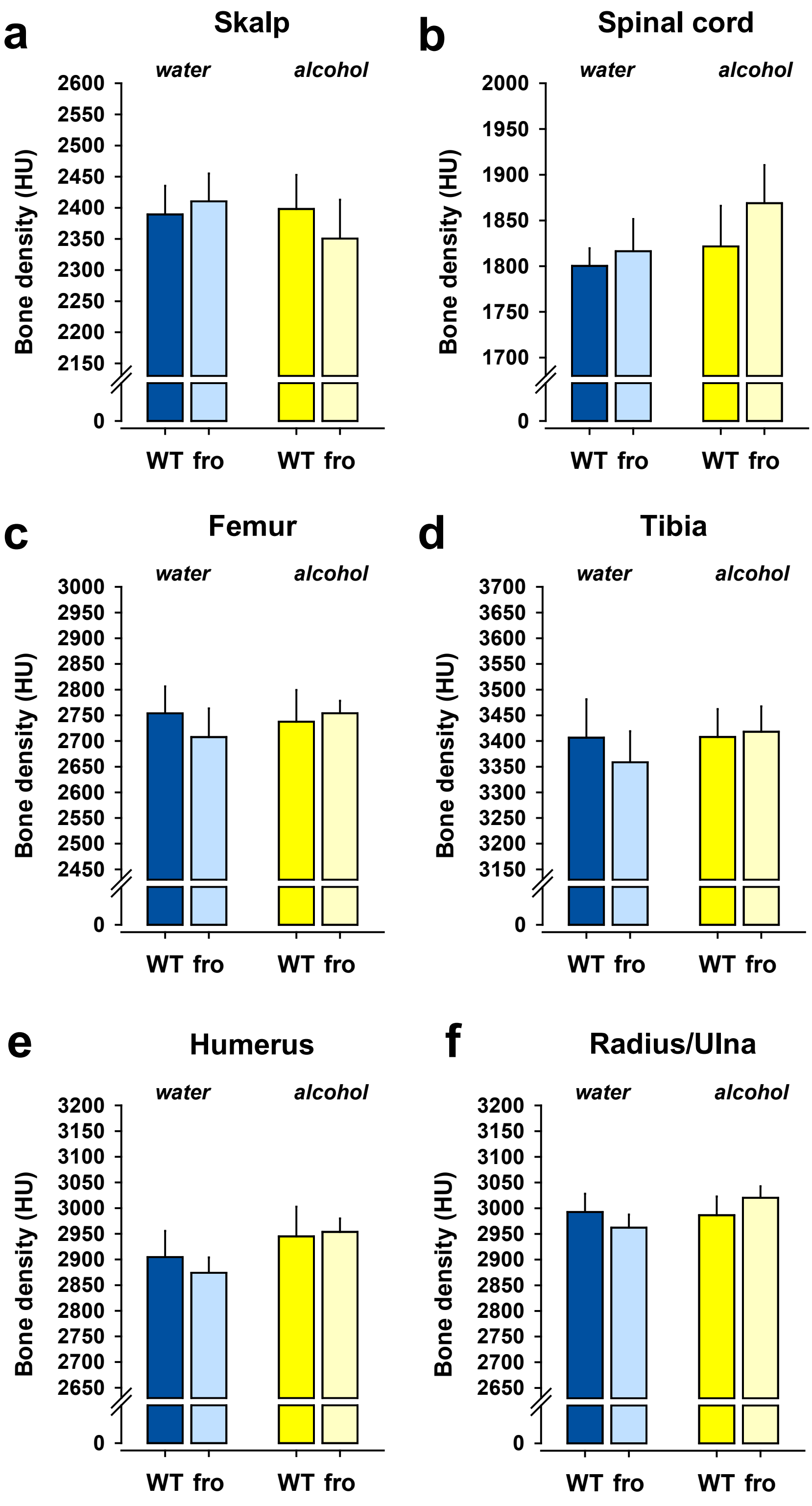

Figure S14 Reduced function of neutral sphingomyelinase-2 (NSM) in heterozygous NSM knock out (fro) mice has no effect on bone density (WT- wild type). Chronic alcohol consumption did not affect bone mineralization in female mice.

Supplementary note 1

RNA-Seq analysis in juvenile primary osteoblasts

In total we found 2622 significantly dysregulated genes in the osteoblasts of fro mice. We did not find significant changes in the expression of osteocalcin gene. However, the previously observed increase in the serum protein level of osteocalcin in fro mice might be associated with an indirect effect of several mediators of its synthesis. In particular dysregulation of the expression of IL-6 receptors and β2-adrenergic receptors as well as IFN-γ signalling pathway in osteoblasts may contribute to the observed osteocalcin up-regulation (Goodman et al., 1999; Kusec et al., 2004).

Fro mice were characterized by enhanced expression of IL-6 (fold 0.136, p=0.021) and reduced expression of its receptor IL-6Rα (fold 2.46, p=8.82*10-7). The expression of α2-adrenergic receptors in the osteoblast cells of fro mice was significantly enhanced (fold 0.542, p=0,009). Several genes responsible for IFN-γ signalling, such as IFN activated gene 203, and IFN regulatory factors 4, 5, and 7, are characterized by significantly reduced expression in osteoblasts of fro mice compared to wt littermates (fold 3,440, p=8.5*10-21; fold 2.930, p=6.4*10-5; fold 2.384, p=6.54*10-5; fold 2.255, p=0.0001). In line with these data, the 'interferon signalling' canonical pathway is also shown to be altered in fro mice (ratio: 0.194, p=10-2.5). Taking into account that IFN treatment affects osteocalcin level in patients (Goodman et al., 1999; Kusec et al., 2004), this cytokine might affect osteocalcin production on posttranslational level.

Another possible mediator of the observed changes in osteocalcin level is osteocrin. Osteocrin is a vitamin D-regulated bone-specific protein regulating the osteoblast functions. Literature data show that the exposure of osteoblastic cell cultures to osteocrin inhibits bone mineralization and induces a reduction in osteocalcin expression in osteoblasts (Thomas et al., 2003). Thus, higher level of serum osteocalcin in fro mice might be at least partly mediated by a slight increase in osteocrin expression in osteoblasts (fold 0.071, p=0.032).

Ingenuity analysis yielded canonical pathways including the 'osteoarthritis pathway' (ratio: 0.095, p=10-2.45), the 'artherosclerosis signalling pathway' (ratio: 0.104, p=10-1.9), and the 'role of osteoblasts in rheuma' (ratio: 0.0853, p=10-1.89) (Suppl. Fig. 33-36). In particular, an upregulation of the osteoarthritis pathway can be responsible for upregulation of osteocalcin levels.

Supplementary note 1

RNA-Seq analysis in juvenile primary osteoblasts

Interestingly, certain alterations in gene functioning found in the osteoblasts of Fro mice might at least partly contribute to the behavioural patterns observed in these animals. For example, functioning of the canonical pathway ‘G-protein coupled receptor signalling’ based on such receptors as Gq, Gs, and Gi is shown to be affected in Fro mice (ratio: 0.091, p=10-2.69). The analysis of single gene expression showed significant downregulation of the expression of G-protein coupled receptors 160, 183, 35, 132, 84, 137b, 34, 146, and 157 (fold 3.762, p=6.01*10-7; fold 0.204, p=1.31*10-5; fold 2.100, p=4.64*10-5; fold 0.111, p=7.88*10-5; fold 1.96, p=0.0005; fold 1.356, p=0.0007; fold 0.059, p=0.001; fold 3.762, p=0.008; fold 0.431, p=0.017) and upregulation of the expression of G-protein coupled receptors 1, 39, 5B, and 37-like 1 (fold 0.801, p=0.0065; fold 0.755, p=0.008; fold 0.387, p=0.024; fold 0.075, p=0.03) in fro mice. The observed changes might determine the alterations in monoaminergic system functioning, which effects are mostly mediated by these types of receptor. Monoamines significantly contribute to the pathogenesis of anxiety/depression and alcohol misuse as well as the interaction between these disorders, thus the osteoblast-mediated alterations in monoamine signalling might affect the behavioural pattern of Fro mice. Another possible pathway of bone-driven alterations in the behavioural phenotype of mice with NSM hypoexpression might be mediated by the alterations in the expression of genes of the ‘Nf-κB signalling’ canonical pathway (ratio: 0.094, p=10-2.11), even though the expression of Nf-κB is not altered. Previous data indicate that Nf-κB is a potential mediator of interaction between stress-induced depression and alcohol misuse in rodents. It should be also noted that lack of NSM is followed by the alteration of several inflammatory canonical pathways mediated by osteoblasts, such as the ‘neuroinflammation signalling pathway’ (ratio: 0.0959, p=10-3.21), ‘osteoarthritis pathway’ (ratio: 0.095, p=10-2.45), ‘interferon signalling pathway’ (ratio: 0.194, p=10-2.5), and others. The importance of inflammatory mediators involved in these pathways, particularly cytokines, Nf-κB, and TLR receptors, in the pathogenesis of depression and alcohol misuse was widely discussed previously. Another possible pathway, which might mediate the effects of osteocalcin on the central nervous system, is related to GABA. Previous data show osteocalcin crosses the blood–brain barrier, and inhibits synthesis of γ-aminobutyric acid (GABA) (Oury et al., 2013). Our study shows that fro mice with increased osteocalcin level in the serum are characterized by enhanced expression of the subunit epsilon of GABA A receptor (fold 0.272, p=0.023) and GABA B receptor (fold 0.339, p=0.027) in osteoblasts. Altogether, NSM-induced alterations in the osteoblast gene expression in Fro mice might have a systemic effect on the inflammatory and non-inflammatory pathways determining emotional state and drinking phenotype.

Supplementary note 1

RNA-Seq analysis in juvenile primary osteoblasts

Thomas G, Moffatt P, Salois P, Gaumond MH, Gingras R, Godin E, Miao D, Goltzman D, Lanctôt C. Osteocrin, a novel bone-specific secreted protein that modulates the osteoblast phenotype. J Biol Chem. 2003 Dec 12;278(50):50563-71.

Goodman GR, Dissanayake IR, Gorodetsky E, Zhou H, Ma YF, Jee WS, Epstein S. Interferon-alpha, unlike interferon-gamma, does not cause bone loss in the rat. Bone. 1999 Oct;25(4):459-63.

Kusec R, Kusec V, Gisslinger B, Woloszczuk W, Gisslinger H. Bone metabolism during interferon-alpha treatment of essential thrombocythemia. Wien Klin Wochenschr. 2004 Jan 31;116(1-2):37-41.

Oury F, Khrimian L, Denny CA, Gardin A, Chamouni A, Goeden N, et al. Maternal and offspring pools of osteocalcin influence brain development and functions. Cell. 2013;155:228–41.

Canonical pathways significantly (p<0.05) altered in osteoblasts of Fro mice

Ingenuity Canonical Pathways	-log(p-val	Ratio	Molecules
Cell Cycle Control of Chromosomal Replication	6,25E00	2,55E-01	CDC45,CDC6,CDK1,DBF4,DNA2,LIG1,MCM2,MCM3,MCM5,MCM6,ORC1,POLE,TOP2A
Cell Cycle: G2/M DNA Damage Checkpoint Regulation	5,72E00	2,5E-01	AURKA,BRCA1,CCNB1,CCNB2,CDC25C,CDK1,CHEK1,CKS2,GADD45A,PLK1,RPS6KA1,TOP2A
Role of Pattern Recognition Receptors in Recognition of Bacte	5,64E00	1,53E-01	CASP1,IFIH1,IRF7,NLRP3,NOD2,OAS2,OSM,PIK3CD,PIK3R6,PLCG2,PRKCB,PRKCH,RNASEL,SYK,TLR1,TLR2,TLR5,TLR7,TL
Role of BRCA1 in DNA Damage Response	4,16E00	1,69E-01	BRCA1,BRIP1,CHEK1,E2F2,E2F7,E2F8,FANCA,GADD45A,PLK1,RAD51,RFC5,STAT1,TOPBP1
TREM1 Signaling	4,15E00	1,79E-01	CASP1,CD40,Naip1 (includes others),NLRP3,NOD2,PLCG2,TLR1,TLR2,TLR5,TLR7,TLR9,TREM1
Phagosome Formation	4,02E00	1,42E-01	FCGR3A/FCGR3B,ITGA2,ITGA4,PIK3CD,PIK3R6,PLCG2,PLCL2,PRKCB,PRKCH,RHOH,SYK,TLR1,TLR2,TLR5,TLR7,TLR9
Mitotic Roles of Polo-Like Kinase	3,88E00	1,8E-01	CCNB1,CCNB2,CDC20,CDC25C,CDK1,ESPL1,FBXO5,KIF11,KIF23,PLK1,PRC1
Role of CHK Proteins in Cell Cycle Checkpoint Control	3,74E00	1,89E-01	BRCA1,CDC25C,CDK1,CHEK1,CLSPN,E2F2,E2F7,E2F8,PLK1,RFC5
Inflammasome pathway	3,72E00	3,16E-01	CASP1,CTSB,NLRP3,NOD2,PANX1,PYCARD
Leukocyte Extravasation Signaling	3,59E00	1,12E-01	ACTG2,ARHGAP12,ARHGAP4,ITGA2,ITGA4,ITGA6,MMP27,MMP3,MMP8,NCF1,PIK3CD,PIK3R6,PLCG2,PRKCB,PRKCH,P
GP6 Signaling Pathway	3,49E00	1,33E-01	APBB1IP,COL10A1,COL2A1,COL9A1,COL9A2,COL9A3,LAMA1,LCP2,LYN,PIK3CD,PIK3R6,PLCG2,PRKCB,PRKCH,SYK
Fcγ Receptor-mediated Phagocytosis in Macrophages and Mor	3,37E00	1,41E-01	ACTG2,FCGR3A/FCGR3B,FYB1,INPP5D,LCP2,LYN,MYO5A,NCF1,PRKCB,PRKCH,PTK2B,SYK,VAV3
Role of Macrophages, Fibroblasts and Endothelial Cells in Rhe	3,26E00	9,4E-02	CEBPA,DKK2,FCGR3A/FCGR3B,FGF2,FZD10,FZD3,IL6R,LEF1,MMP3,NFATC2,OSM,PDGFB,PIK3CD,PIK3R6,PLCG2,PLCL2,
Communication between Innate and Adaptive Immune Cells	3,22E00	1,53E-01	CCL3L3,CD40,HLA-A,HLA-G,IGHM,TLR1,TLR2,TLR5,TLR7,TLR9,TNFSF13B
Neuroinflammation Signaling Pathway	3,21E00	9,59E-02	BIRC5,CASP1,CD200R1,CD40,GDNF,HLA-A,IL6R,IRF7,MMP3,Naip1 (includes others),NFATC2,NLRP3,PIK3CD,PIK3R6,P
Virus Entry via Endocytic Pathways	3,1E00	1,33E-01	ACTG2,AP1S2,HLA-A,ITGA2,ITGA4,ITGA6,ITGB8,PIK3CD,PIK3R6,PLCG2,PRKCB,PRKCH,RAP2B
Natural Killer Cell Signaling	2,94E00	1,27E-01	FCGR3A/FCGR3B,HCST,INPP5D,LCP2,PIK3CD,PIK3R6,PLCG2,PRKCB,PRKCH,RAP2B,SH3BP2,SYK,VAV3
Estrogen-mediated S-phase Entry	2,92E00	2,31E-01	CCNA2,CCNE2,CDK1,E2F2,E2F7,E2F8
DNA damage-induced 14-3-3σ Signaling	2,9E00	2,78E-01	BRCA1,CCNB1,CCNB2,CCNE2,CDK1
Hepatic Fibrosis / Hepatic Stellate Cell Activation	2,78E00	1,04E-01	Agtr1b,CCN2,CD14,CD40,COL10A1,COL2A1,COL9A1,COL9A2,COL9A3,FGF2,IL6R,LAMA1,MYH8,PDGFB,SMAD7,STAT1,T
GADD45 Signaling	2,78E00	2,63E-01	BRCA1,CCNB1,CCNE2,CDK1,GADD45A
G-Protein Coupled Receptor Signaling	2,69E00	9,09E-02	ADCY7,ADORA1,ADRB2,Agtr1b,AGTR2,CNR2,GDPD1,GNA15,HCAR2,HTR2B,P2RY14,PDE1B,PIK3CD,PIK3R6,PRKCB,PTG
Tec Kinase Signaling	2,58E00	1,05E-01	ACTG2,GNA15,GNG2,ITGA2,ITGA4,LYN,PIK3CD,PIK3R6,PLCG2,PRKCB,PRKCH,PTK2B,RHOH,STAT1,STAT2,VAV3
Activation of IRF by Cytosolic Pattern Recognition Receptors	2,56E00	1,57E-01	CD40,DHX58,IFIH1,IRF7,ISG15,STAT1,STAT2,ZBP1
Interferon Signaling	2,5E00	1,94E-01	IFIT3,IFITM3,ISG15,PSMB8,STAT1,STAT2
Colorectal Cancer Metastasis Signaling	2,47E00	9,02E-02	ADCY7,ARRB1,BIRC5,FZD10,FZD3,GNG2,IL6R,LEF1,MMP27,MMP3,MMP8,PIK3CD,PIK3R6,RAP2B,RHOH,STAT1,TLR1,TL
Osteoarthritis Pathway	2,45E00	9,5E-02	ANKH,CASP1,CNMD,COL10A1,COL2A1,FGF2,FZD10,FZD3,H19,HES1,ITGA2,ITGA4,LEF1,MMP3,NKX3-2,RBPJ,SMAD7,TL
DNA Double-Strand Break Repair by Homologous Recombinati	2,45E00	2,86E-01	BRCA1,GEN1,LIG1,RAD51
Reelin Signaling in Neurons	2,44E00	1,3E-01	APOE,CNR2,ITGA2,ITGA4,ITGA6,LYN,MAP3K9,PIK3CD,PIK3R6,RELN
Molecular Mechanisms of Cancer	2,41E00	8,06E-02	ADCY7,Aph1c,AURKA,BMP5,BMP7,BRCA1,CCNE2,CDC25C,CDK1,CHEK1,E2F2,E2F7,E2F8,FZD10,FZD3,GNA15,ITGA2,ITG
T Helper Cell Differentiation	2,39E00	1,36E-01	CD40,GATA3,HLA-A,ICOSLG/LOC102723996,IL10RB,IL2RG,IL6R,STAT1,TNFRSF1B
T Cell Exhaustion Signaling Pathway	2,13E00	0,1	CD274,HLA-A,HLA-G,IL10RB,IL6R,IRF4,NFATC2,PIK3CD,PIK3R6,PLCG2,PRDM1,RAP2B,STAT1,STAT2
NF-κB Signaling	2,11E00	9,41E-02	CD40,MAP3K1,PIK3CD,PIK3R6,PLCG2,PRKCB,RAP2B,TGFA,TLR1,TLR2,TLR5,TLR7,TLR9,TNFRSF11A,TNFRSF1B,TNFSF13I
Factors Promoting Cardiogenesis in Vertebrates	2,1E00	1,16E-01	BMP5,BMP7,CCNE2,CDC6,FZD10,FZD3,LEF1,PRKCB,PRKCH,WNT11
Dendritic Cell Maturation	2,09E00	9,62E-02	CD40,COL10A1,COL2A1,FCGR3A/FCGR3B,HLA-A,IRF8,PIK3CD,PIK3R6,PLCG2,PLCL2,STAT1,STAT2,TLR2,TLR9,TNFRSF1B
Production of Nitric Oxide and Reactive Oxygen Species in Ma	2,04E00	9,25E-02	APOE,CLU,IRF8,MAP3K1,MAP3K9,NCF1,PIK3CD,PIK3R6,PLCG2,PRKCB,PRKCH,RHOH,SIRPA,STAT1,TLR2,TNFRSF1B
Role of Tissue Factor in Cancer	2,04E00	1,04E-01	ARRB1,ARRB2,CCN2,GNA15,HBEGF,ITGA6,LYN,PIK3CD,PIK3R6,PTK2B,RAP2B,RPS6KA1
Macropinocytosis Signaling	2,02E00	1,2E-01	CD14,ITGB8,PDGFB,PIK3CD,PIK3R6,PLCG2,PRKCB,PRKCH,RAP2B
Systemic Lupus Erythematosus Signaling	2	9,14E-02	CD40,FCGR3A/FCGR3B,HLA-A,HLA-G,IGHM,IL6R,INPP5D,LYN,NFATC2,PIK3CD,PIK3R6,PLCG2,RAP2B,TLR7,TLR9,TNFSF
B Cell Receptor Signaling	2	9,14E-02	APBB1IP,BCL2A1,IGHM,INPP5D,LYN,MAP3K1,MAP3K9,NFATC2,PIK3CD,PIK3R6,PLCG2,PRKCB,PTK2B,RAP2B,SYK,VAV
ATM Signaling	1,93E00	1,1E-01	BRCA1,CCNB1,CCNB2,CDC25C,CDK1,CHEK1,GADD45A,RAD51,SMC2,TOPBP1
Atherosclerosis Signaling	1,9E00	1,04E-01	ALOX5,APOE,CCR3,CD40,CLU,COL10A1,COL2A1,ITGA4,MMP3,PDGFB,PLA2G7
Heme Degradation	1,89E00	0,5	BLVRA,BLVRB
Role of Osteoblasts, Osteoclasts and Chondrocytes in Rheuma	1,89E00	8,53E-02	BMP5,BMP7,DKK2,FZD10,FZD3,ITGA2,LEF1,MMP3,MMP8,Naip1 (includes others),NFATC2,PIK3CD,PIK3R6,PTK2B,SFR
Leukotriene Biosynthesis	1,87E00	2,73E-01	ALOX5,LTC4S,MGST3
Crosstalk between Dendritic Cells and Natural Killer Cells	1,8E00	1,18E-01	ACTG2,CD40,HLA-A,HLA-G,IL2RG,TLR7,TLR9,TNFRSF1B
Glioma Signaling	1,79E00	0,1	E2F2,E2F7,E2F8,PDGFB,PIK3CD,PIK3R6,PLCG2,PRKCB,PRKCH,RAP2B,TGFA
Th1 Pathway	1,79E00	0,1	CD274,CD40,GATA3,HLA-A,ICOSLG/LOC102723996,IL10RB,IL6R,NFATC2,PIK3CD,PIK3R6,STAT1
UVA-Induced MAPK Signaling	1,78E00	1,04E-01	PARP10,PARP14,PARP9,PIK3CD,PIK3R6,PLCG2,PLCL2,RAP2B,RPS6KA1,STAT1
Growth Hormone Signaling	1,77E00	1,16E-01	CEBPA,PIK3CD,PIK3R6,PLCG2,PRKCB,PRKCH,RPS6KA1,STAT1
Fc Epsilon RI Signaling	1,76E00	9,91E-02	INPP5D,LCP2,LYN,PIK3CD,PIK3R6,PLCG2,PRKCB,PRKCH,RAP2B,SYK,VAV3
NF-κB Activation by Viruses	1,75E00	1,08E-01	ITGA2,ITGA4,ITGA6,MAP3K1,PIK3CD,PIK3R6,PRKCB,PRKCH,RAP2B
Th1 and Th2 Activation Pathway	1,75E00	8,97E-02	CCR3,CD274,CD40,GATA3,GFI1,HLA-A,ICOSLG/LOC102723996,IL10RB,IL2RG,IL6R,NFATC2,PIK3CD,PIK3R6,STAT1
Toll-like Receptor Signaling	1,73E00	1,14E-01	CD14,MAP3K1,TICAM2,TLR1,TLR2,TLR5,TLR7,TLR9
Cardiac Hypertrophy Signaling (Enhanced)	1,71E00	7,03E-02	ADCY7,ADRB2,DIAPH3,FGF2,FZD10,FZD3,GDPD1,GNA15,GNG2,IL10RB,IL2RG,IL6R,ITGA2,ITGA4,MAP3K1,MAP3K9,NFA
GM-CSF Signaling	1,7E00	1,13E-01	BCL2A1,CSF2RA,LYN,PIK3CD,PIK3R6,PRKCB,RAP2B,STAT1
Salvage Pathways of Pyrimidine Ribonucleotides	1,66E00	1,05E-01	AK4,APOBEC1,CDK1,CMPK2,MAP3K9,NEK2,PLK1,PRKCH,TTK
PDGF Signaling	1,66E00	1,05E-01	INPP5D,MAP3K1,PDGFB,PIK3CD,PIK3R6,PLCG2,PRKCB,RAP2B,STAT1
Glioblastoma Multiforme Signaling	1,64E00	8,7E-02	E2F2,E2F7,E2F8,FZD10,FZD3,LEF1,PDGFB,PIK3CD,PIK3R6,PLCG2,PLCL2,RAP2B,RHOH,WNT11
Axonal Guidance Signaling	1,62E00	6,97E-02	BMP5,BMP7,FZD10,FZD3,GNA15,GNG2,ITGA2,ITGA4,L1CAM,MAG,MMP27,MMP3,MMP8,NFATC2,PDGFB,PIK3CD,PIK3
Renin-Angiotensin Signaling	1,61E00	9,4E-02	ADCY7,AGTR2,MAP3K1,PIK3CD,PIK3R6,PLCG2,PRKCB,PRKCH,PTK2B,RAP2B,STAT1
Hereditary Breast Cancer Signaling	1,58E00	9,02E-02	BRCA1,CCNB1,CDC25C,CDK1,CHEK1,FANCA,GADD45A,PIK3CD,PIK3R6,RAD51,RAP2B,RFC5
ErbB Signaling	1,55E00	0,1	EREG,HBEGF,PIK3CD,PIK3R6,PLCG2,PRKCB,PRKCH,RAP2B,TGFA
Cyclins and Cell Cycle Regulation	1,54E00	1,05E-01	CCNA2,CCNB1,CCNB2,CCNE2,CDK1,E2F2,E2F7,E2F8
Pentose Phosphate Pathway (Non-oxidative Branch)	1,52E00	3,33E-01	TALDO1,TKT
Notch Signaling	1,51E00	1,35E-01	DTX4,HES1,MAG,MFNG,RBPJ
Neuregulin Signaling	1,49E00	9,78E-02	EREG,HBEGF,ITGA2,ITGA4,PLCG2,PRKCB,PRKCH,RAP2B,TGFA
Thrombopoietin Signaling	1,48E00	1,09E-01	PIK3CD,PIK3R6,PLCG2,PRKCB,PRKCH,RAP2B,STAT1
Pyrimidine Ribonucleotides Interconversion	1,47E00	1,32E-01	AK4,CMPK2,ENTPD1,RAD54L,ZRANB3
PD-1, PD-L1 cancer immunotherapy pathway	1,47E00	9,68E-02	CD274,CIP2A,HLA-A,HLA-G,IL2RG,LCP2,PIK3CD,PIK3R6,TNFRSF1B
Pancreatic Adenocarcinoma Signaling	1,46E00	9,26E-02	BIRC5,E2F2,E2F7,E2F8,HBEGF,PIK3CD,PIK3R6,RAD51,STAT1,TGFA
Agranulocyte Adhesion and Diapedesis	1,45E00	8,39E-02	ACTG2,Ccl2,CCL3L3,Ccl7,CXCL16,Cxcl9,ITGA2,ITGA4,ITGA6,MMP27,MMP3,MMP8,MYH8
HGF Signaling	1,44E00	9,17E-02	ITGA2,ITGA4,MAP3K1,MAP3K9,PIK3CD,PIK3R6,PLCG2,PRKCB,PRKCH,RAP2B
Altered T Cell and B Cell Signaling in Rheumatoid Arthritis	1,43E00	0,1	CD40,HLA-A,TLR1,TLR2,TLR5,TLR7,TLR9,TNFSF13B
IL-4 Signaling	1,43E00	0,1	HLA-A,IL2RG,INPP5D,IRF4,NFATC2,PIK3CD,PIK3R6,RAP2B
Prolactin Signaling	1,4E00	9,88E-02	NMI,PIK3CD,PIK3R6,PLCG2,PRKCB,PRKCH,RAP2B,STAT1
Pyrimidine Ribonucleotides De Novo Biosynthesis	1,39E00	1,25E-01	AK4,CMPK2,ENTPD1,RAD54L,ZRANB3
B Cell Development	1,37E00	1,43E-01	CD40,HLA-A,IGHM,SPN
Antigen Presentation Pathway	1,37E00	1,43E-01	HLA-A,HLA-G,PSMB8,PSMB9
Human Embryonic Stem Cell Pluripotency	1,36E00	8,59E-02	BMP5,BMP7,FGF2,FZD10,FZD3,LEF1,PDGFB,PIK3CD,PIK3R6,SMAD7,WNT11
Role of JAK1 and JAK3 in γc Cytokine Signaling	1,36E00	1,03E-01	IL2RG,PIK3CD,PIK3R6,PTK2B,RAP2B,STAT1,SYK
Primary Immunodeficiency Signaling	1,35E00	1,22E-01	CD40,DCLRE1C,IGHM,IL2RG,UNG
IL-12 Signaling and Production in Macrophages	1,33E00	8,77E-02	APOE,CD40,CLU,IRF8,PIK3CD,PIK3R6,PRKCB,PRKCH,STAT1,TLR2
Thio-molybdenum Cofactor Biosynthesis	1,32E00	1	MOCOS
Oncostatin M Signaling	1,31E00	1,19E-01	MMP3,OSM,PLAU,RAP2B,STAT1
Granulocyte Adhesion and Diapedesis	1,3E00	8,16E-02	Ccl2,CCL3L3,Ccl7,CXCL16,Cxcl9,ITGA2,ITGA4,ITGA6,MMP27,MMP3,MMP8,TNFRSF1B

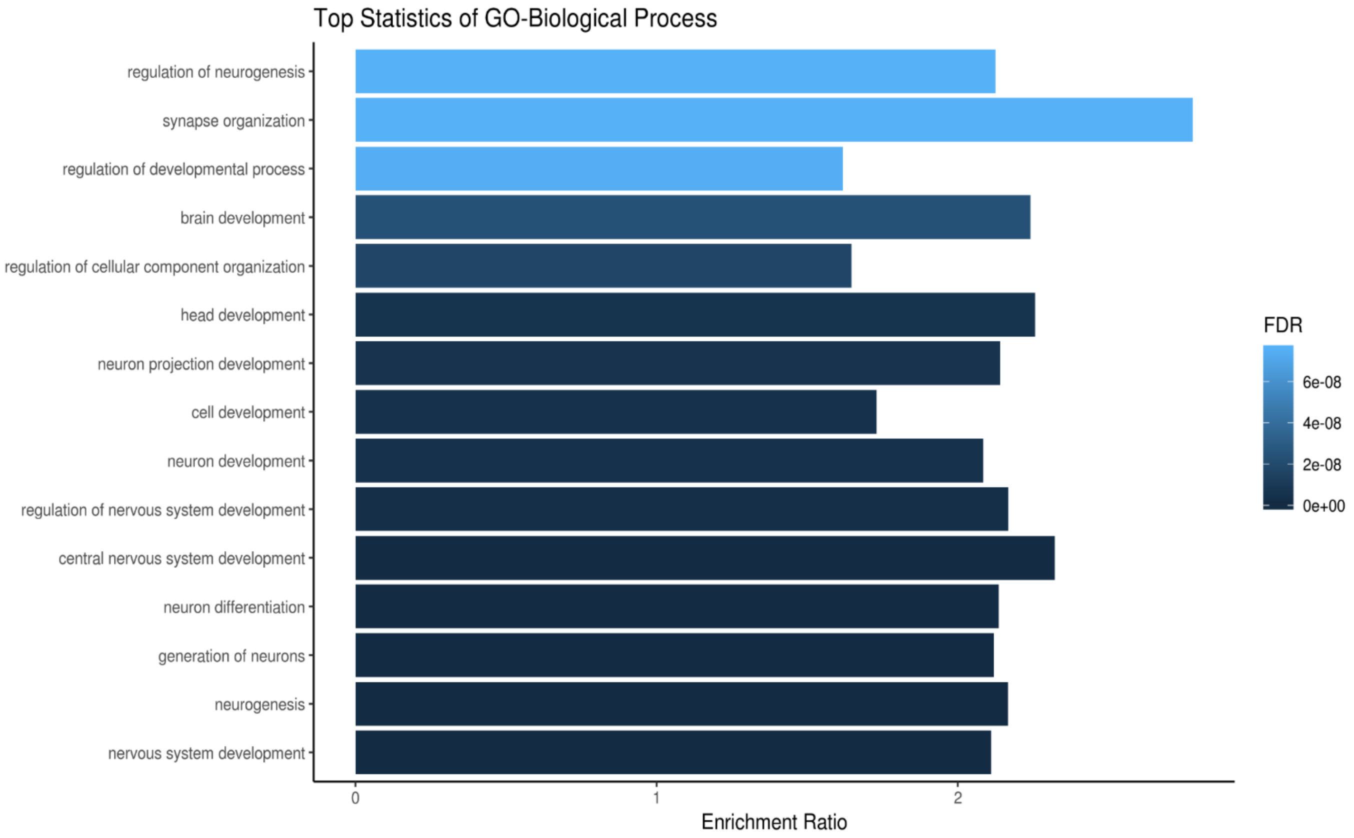

Figure S15 Gene ontology (GO) of biological processes affected by heterozygous knock out of neutral sphingomyelinase-2 in osteoblasts of mice.

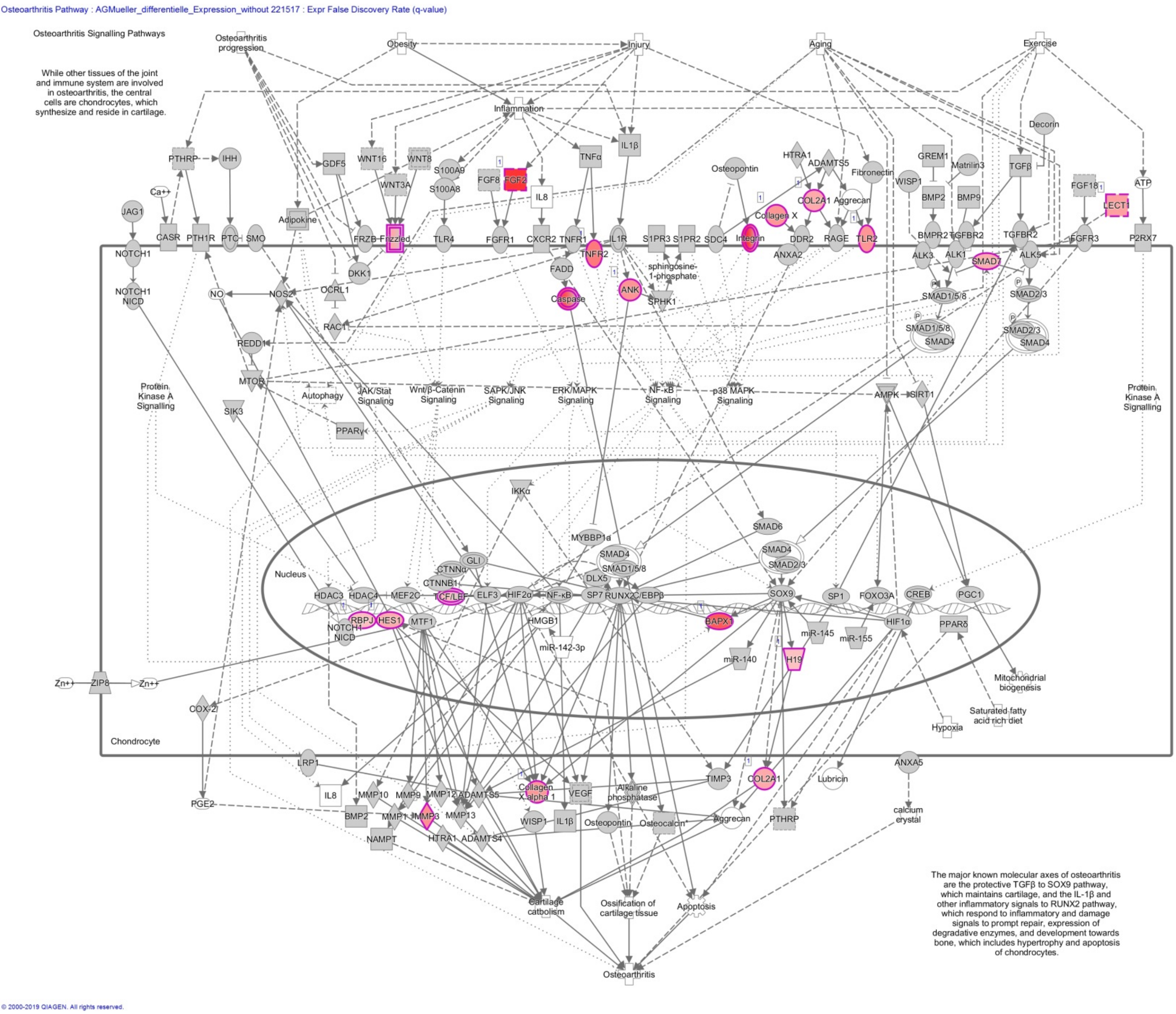

Figure S16 Osteparthritis pathway affected by heterozygous knock out of neutral sphingomyelinase-2 in osteoblasts of mice.

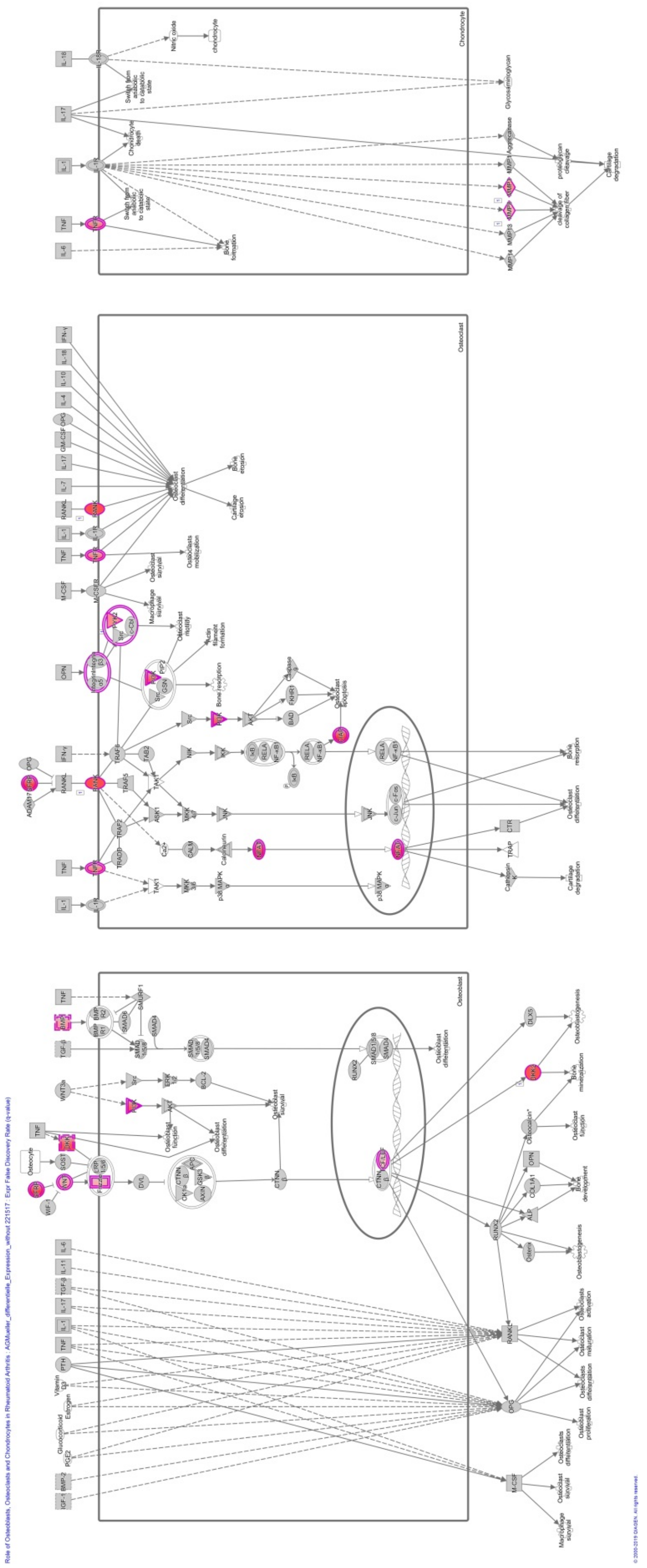

Figure S17 Bone cells in arthritis pathway affected by heterozygous knock out of neutral sphingomyelinase-2 in osteoblasts of mice.

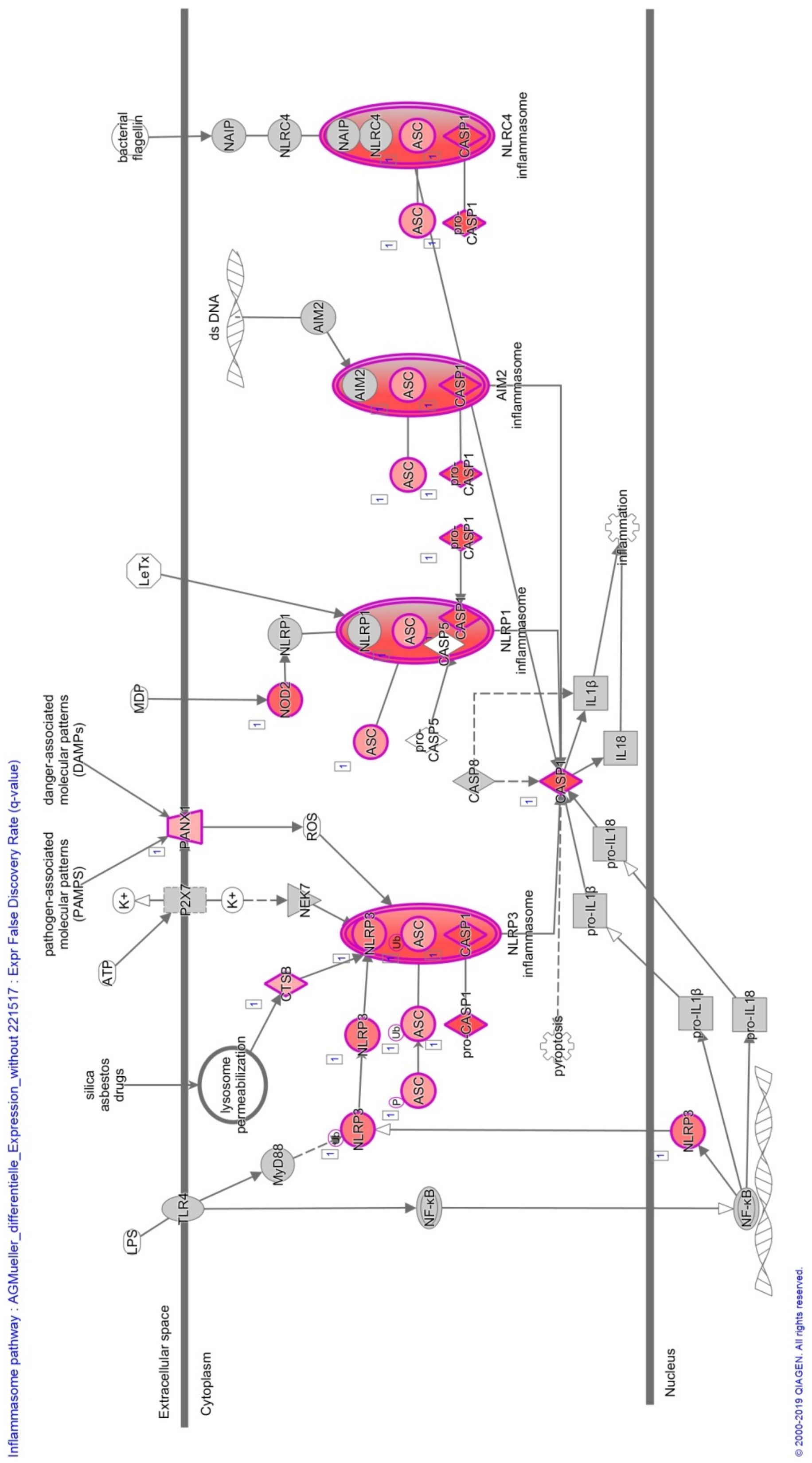

Figure S18 Inflammasome pathway affected by heterozygous knock out of neutral sphingomyelinase-2 in osteoblasts of mice.

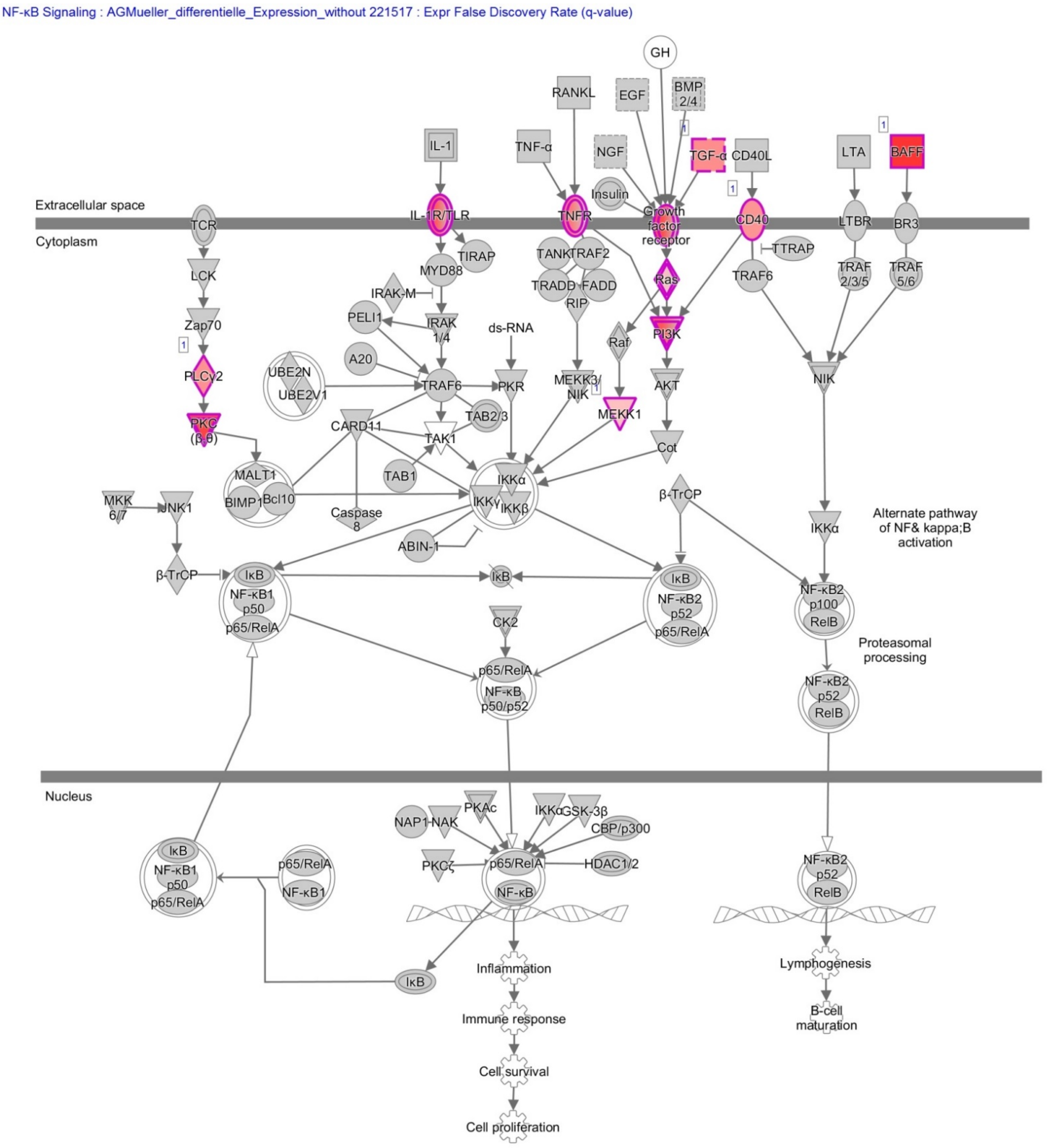

© 2000-2019 QIAGEN. All rights reserved.

Figure S19 NF-kB signalling pathway affected by heterozygous knock out of neutral sphingomyelinase-2 in osteoblasts of mice.

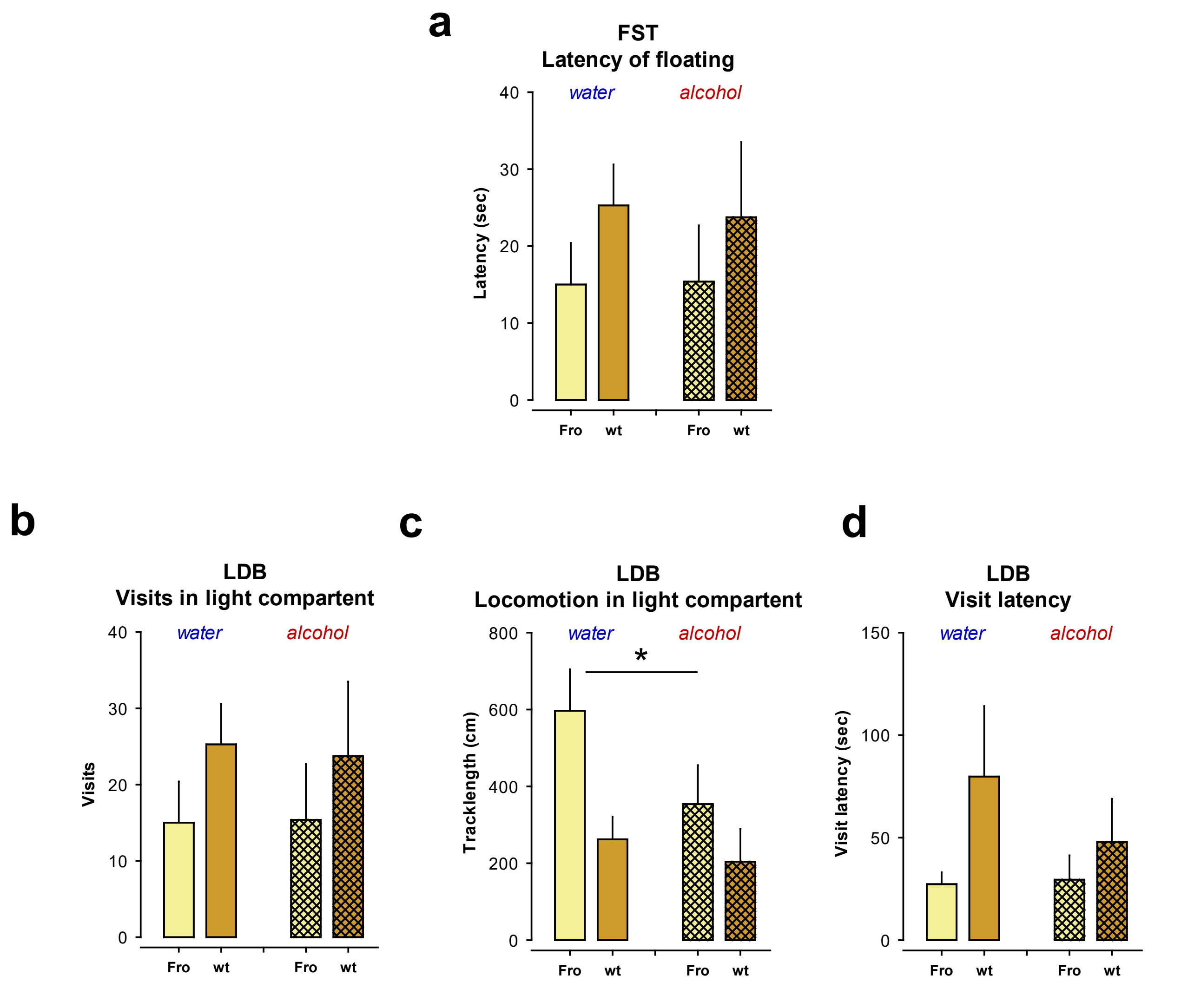

Figure S20 Self-regulation of emotional state with alcohol is controlled by neutral sphingomyelinase-2 (NSM) in mice with a heterozygous NSM knock out (fro) tested in **a**, the forced swim test (FST) and **b-d**, the light-dark box test (LDB; WT-wild type; *p<0.05).

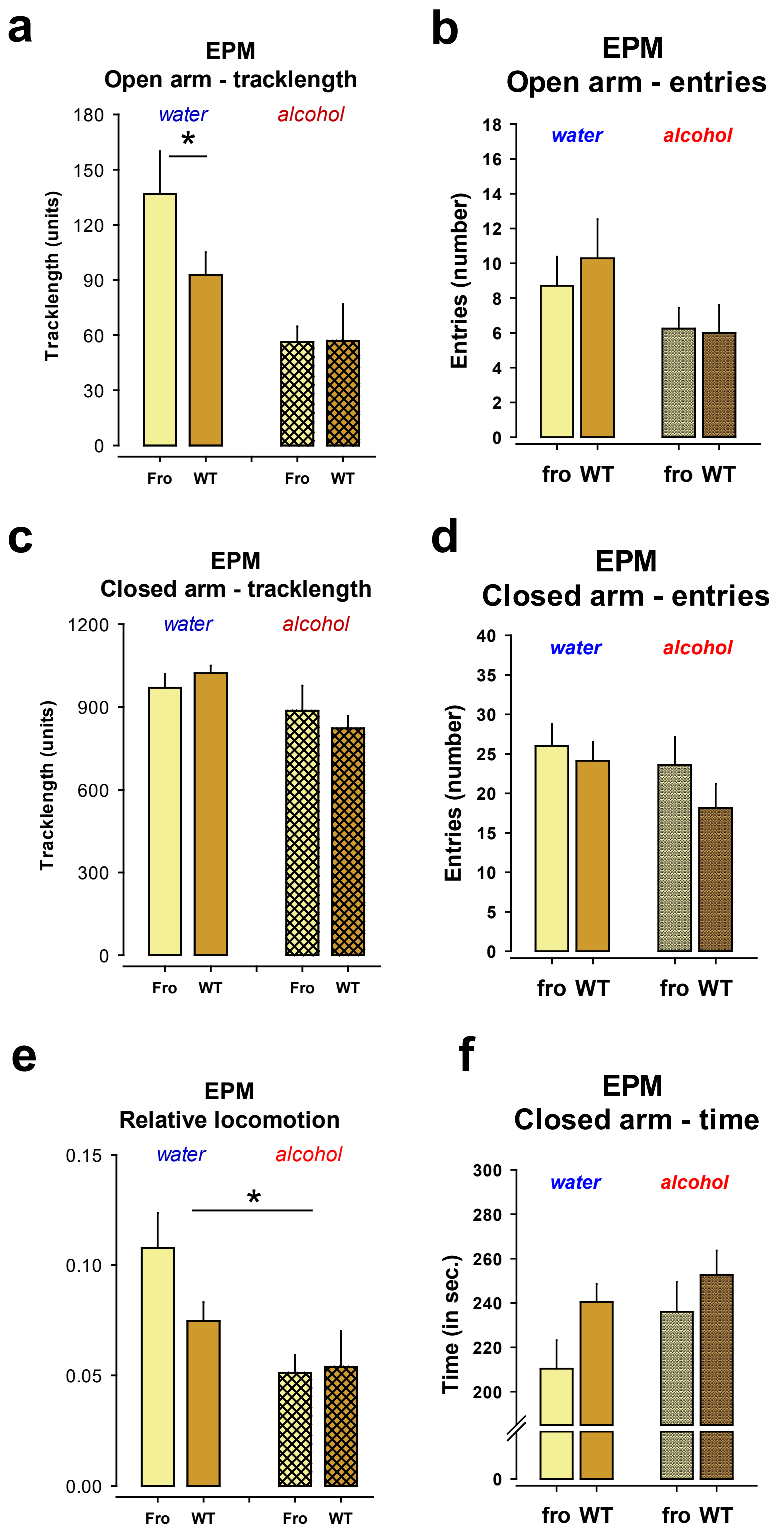

Figure S21 Self-regulation of emotional state with alcohol is controlled by neutral sphingomyelinase-2 (NSM) in mice with a heterozygous NSM knock out (fro) tested in the elevated plus maze (EPM; WT-wild type; *p<0.05).

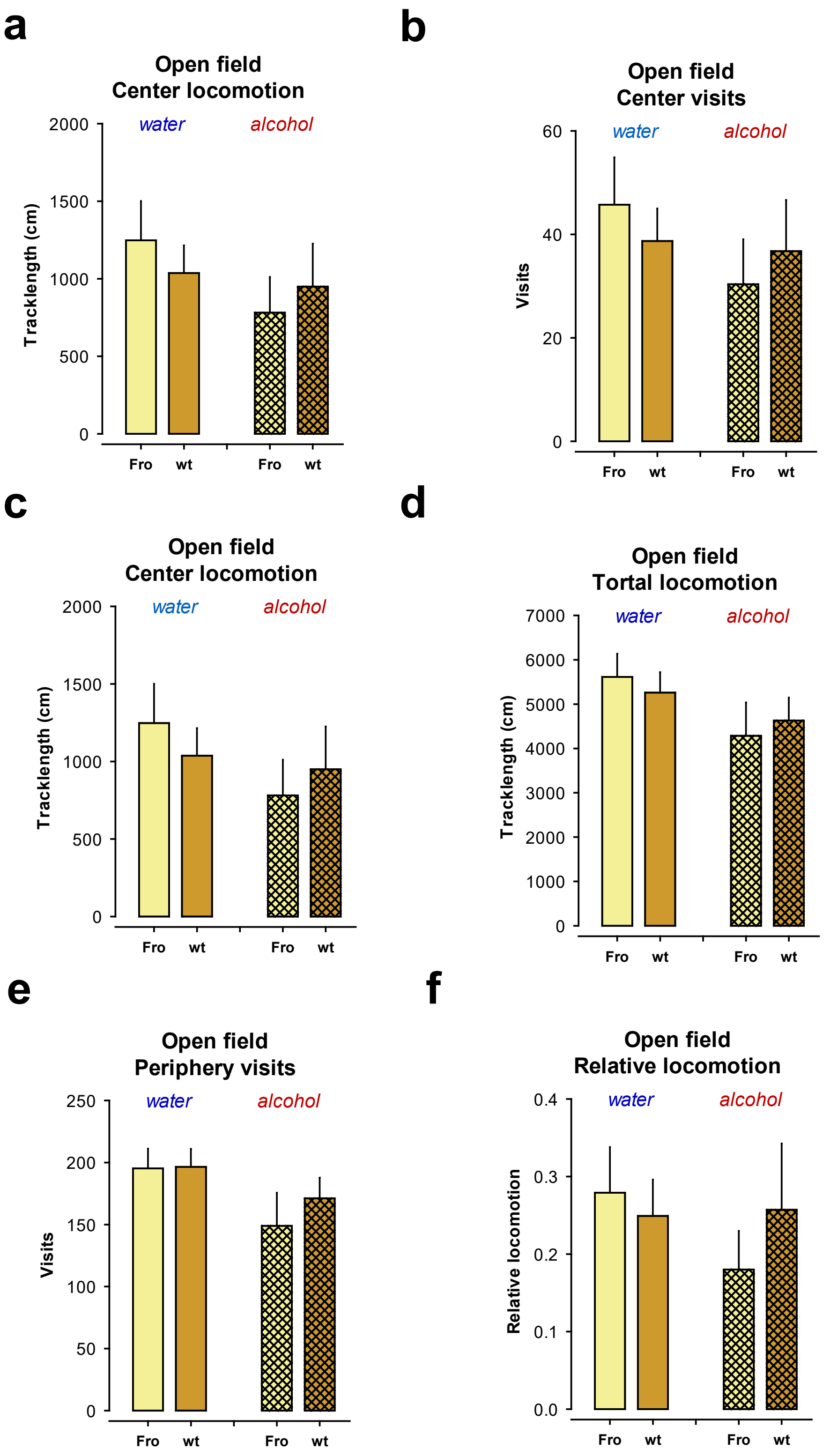

Figure S22 Self-regulation of emotional state with alcohol is controlled by neutral sphingomyelinase-2 (NSM) in mice with a heterozygous NSM knock out (fro) tested in the open field test (WT-wild type; *$p<0.05$).

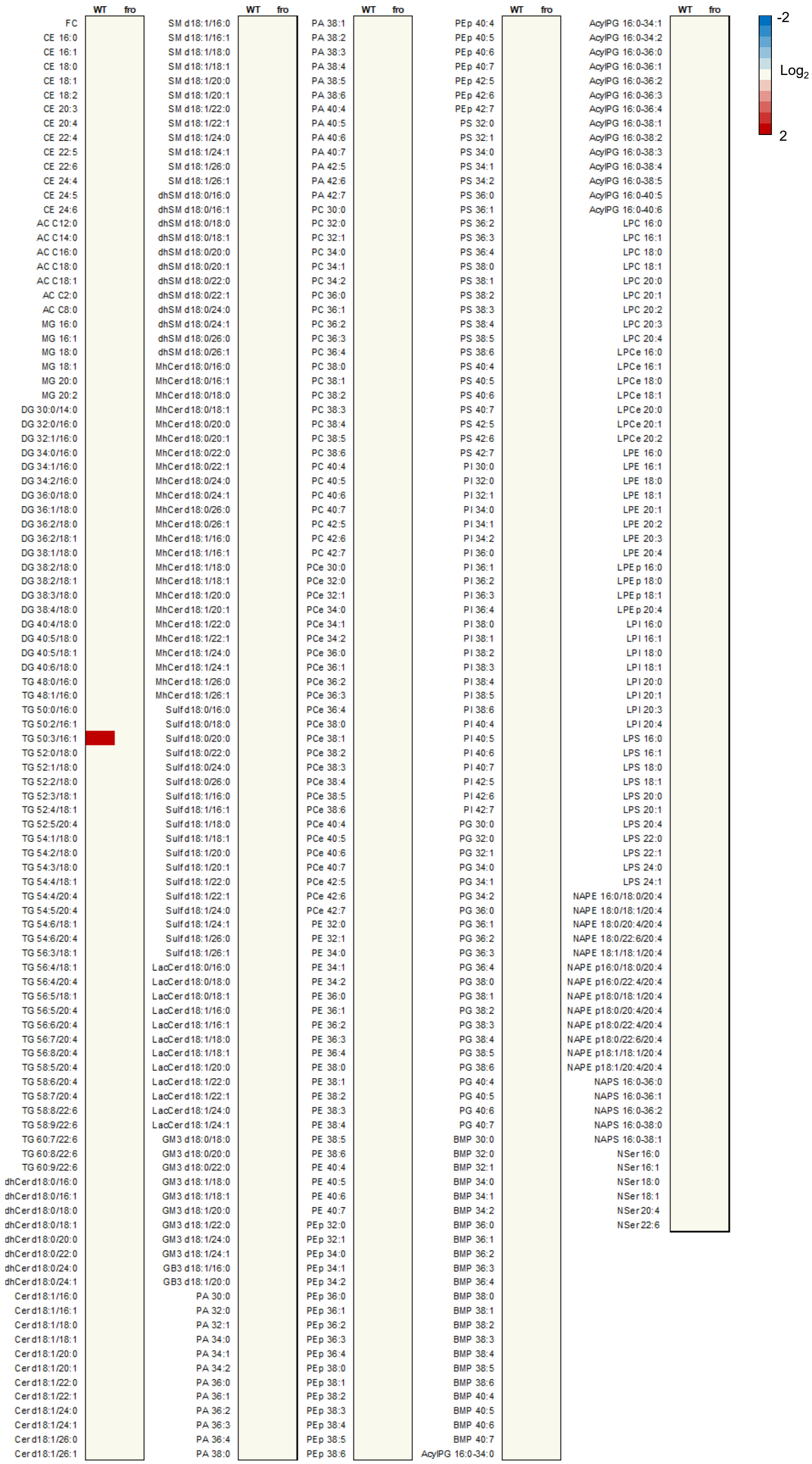
WT
fro
-2
Log2
2
FC
CE 16:0
CE 16:1
CE 18:0
CE 18:1
CE 18:2
CE 20:3
CE 20:4
CE 22:4
CE 22:5
CE 22:6
CE 24:4
CE 24:5
CE 24:6
AC C12:0
AC C14:0
AC C16:0
AC C18:0
AC C18:1
AC C2:0
AC C8:0
MG 16:0
MG 16:1
MG 18:0
MG 18:1
MG 20:0
MG 20:2
DG 30:0/14:0
DG 32:0/16:0
DG 32:1/16:0
DG 34:0/16:0
DG 34:1/16:0
DG 34:2/16:0
DG 36:0/18:0
DG 36:1/18:0
DG 36:2/18:0
DG 36:2/18:1
DG 38:1/18:0
DG 38:2/18:0
DG 38:2/18:1
DG 38:3/18:0
DG 38:4/18:0
DG 40:4/18:0
DG 40:5/18:0
DG 40:5/18:1
DG 40:6/18:0
TG 48:0/16:0
TG 48:1/16:0
TG 50:0/16:0
TG 50:2/16:1
TG 50:3/16:1
TG 52:0/18:0
TG 52:1/18:0
TG 52:2/18:0
TG 52:3/18:1
TG 52:4/18:1
TG 52:5/20:4
TG 54:1/18:0
TG 54:2/18:0
TG 54:3/18:0
TG 54:4/18:1
TG 54:4/20:4
TG 54:5/20:4
TG 54:6/18:1
TG 54:6/20:4
TG 56:3/18:1
TG 56:4/18:1
TG 56:4/20:4
TG 56:5/18:1
TG 56:5/20:4
TG 56:6/20:4
TG 56:7/20:4
TG 56:8/20:4
TG 58:5/20:4
TG 58:6/20:4
TG 58:7/20:4
TG 58:8/22:6
TG 58:9/22:6
TG 60:7/22:6
TG 60:8/22:6
TG 60:9/22:6
dhCer d18:0/16:0
dhCer d18:0/16:1
dhCer d18:0/18:0
dhCer d18:0/18:1
dhCer d18:0/20:0
dhCer d18:0/22:0
dhCer d18:0/24:0
dhCer d18:0/24:1
Cer d18:1/16:0
Cer d18:1/16:1
Cer d18:1/18:0
Cer d18:1/18:1
Cer d18:1/20:0
Cer d18:1/20:1
Cer d18:1/22:0
Cer d18:1/22:1
Cer d18:1/24:0
Cer d18:1/24:1
Cer d18:1/26:0
Cer d18:1/26:1
WT
fro
SM d18:1/16:0
SM d18:1/16:1
SM d18:1/18:0
SM d18:1/18:1
SM d18:1/20:0
SM d18:1/20:1
SM d18:1/22:0
SM d18:1/22:1
SM d18:1/24:0
SM d18:1/24:1
SM d18:1/26:0
SM d18:1/26:1
dhSM d18:0/16:0
dhSM d18:0/16:1
dhSM d18:0/18:0
dhSM d18:0/18:1
dhSM d18:0/20:0
dhSM d18:0/20:1
dhSM d18:0/22:0
dhSM d18:0/22:1
dhSM d18:0/24:0
dhSM d18:0/24:1
dhSM d18:0/26:0
dhSM d18:0/26:1
MhCer d18:0/16:0
MhCer d18:0/16:1
MhCer d18:0/18:0
MhCer d18:0/18:1
MhCer d18:0/20:0
MhCer d18:0/20:1
MhCer d18:0/22:0
MhCer d18:0/22:1
MhCer d18:0/24:0
MhCer d18:0/24:1
MhCer d18:0/26:0
MhCer d18:0/26:1
MhCer d18:1/16:0
MhCer d18:1/16:1
MhCer d18:1/18:0
MhCer d18:1/18:1
MhCer d18:1/20:0
MhCer d18:1/20:1
MhCer d18:1/22:0
MhCer d18:1/22:1
MhCer d18:1/24:0
MhCer d18:1/24:1
MhCer d18:1/26:0
MhCer d18:1/26:1
Sulf d18:0/16:0
Sulf d18:0/18:0
Sulf d18:0/20:0
Sulf d18:0/22:0
Sulf d18:0/24:0
Sulf d18:0/26:0
Sulf d18:1/16:0
Sulf d18:1/16:1
Sulf d18:1/18:0
Sulf d18:1/18:1
Sulf d18:1/20:0
Sulf d18:1/20:1
Sulf d18:1/22:0
Sulf d18:1/22:1
Sulf d18:1/24:0
Sulf d18:1/24:1
Sulf d18:1/26:0
Sulf d18:1/26:1
LacCer d18:0/16:0
LacCer d18:0/18:0
LacCer d18:0/18:1
LacCer d18:1/16:0
LacCer d18:1/16:1
LacCer d18:1/18:0
LacCer d18:1/18:1
LacCer d18:1/20:0
LacCer d18:1/22:0
LacCer d18:1/22:1
LacCer d18:1/24:0
LacCer d18:1/24:1
GM3 d18:0/18:0
GM3 d18:0/20:0
GM3 d18:0/22:0
GM3 d18:1/18:0
GM3 d18:1/18:1
GM3 d18:1/20:0
GM3 d18:1/22:0
GM3 d18:1/24:0
GM3 d18:1/24:1
GB3 d18:1/16:0
GB3 d18:1/20:0
PA 30:0
PA 32:0
PA 32:1
PA 34:0
PA 34:1
PA 34:2
PA 36:0
PA 36:1
PA 36:2
PA 36:3
PA 36:4
PA 38:0
WT
fro
PA 38:1
PA 38:2
PA 38:3
PA 38:4
PA 38:5
PA 38:6
PA 40:4
PA 40:5
PA 40:6
PA 40:7
PA 42:5
PA 42:6
PA 42:7
PC 30:0
PC 32:0
PC 32:1
PC 34:0
PC 34:1
PC 34:2
PC 36:0
PC 36:1
PC 36:2
PC 36:3
PC 36:4
PC 38:0
PC 38:1
PC 38:2
PC 38:3
PC 38:4
PC 38:5
PC 38:6
PC 40:4
PC 40:5
PC 40:6
PC 40:7
PC 42:5
PC 42:6
PC 42:7
PCe 30:0
PCe 32:0
PCe 32:1
PCe 34:0
PCe 34:1
PCe 34:2
PCe 36:0
PCe 36:1
PCe 36:2
PCe 36:3
PCe 36:4
PCe 38:0
PCe 38:1
PCe 38:2
PCe 38:3
PCe 38:4
PCe 38:5
PCe 38:6
PCe 40:4
PCe 40:5
PCe 40:6
PCe 40:7
PCe 42:5
PCe 42:6
PCe 42:7
PE 32:0
PE 32:1
PE 34:0
PE 34:1
PE 34:2
PE 36:0
PE 36:1
PE 36:2
PE 36:3
PE 36:4
PE 38:0
PE 38:1
PE 38:2
PE 38:3
PE 38:4
PE 38:5
PE 38:6
PE 40:4
PE 40:5
PE 40:6
PE 40:7
PEp 32:0
PEp 32:1
PEp 34:0
PEp 34:1
PEp 34:2
PEp 36:0
PEp 36:1
PEp 36:2
PEp 36:3
PEp 36:4
PEp 38:0
PEp 38:1
PEp 38:2
PEp 38:3
PEp 38:4
PEp 38:5
PEp 38:6
WT
fro
PEp 40:4
PEp 40:5
PEp 40:6
PEp 40:7
PEp 42:5
PEp 42:6
PEp 42:7
PS 32:0
PS 32:1
PS 34:0
PS 34:1
PS 34:2
PS 36:0
PS 36:1
PS 36:2
PS 36:3
PS 36:4
PS 38:0
PS 38:1
PS 38:2
PS 38:3
PS 38:4
PS 38:5
PS 38:6
PS 40:4
PS 40:5
PS 40:6
PS 40:7
PS 42:5
PS 42:6
PS 42:7
PI 30:0
PI 32:0
PI 32:1
PI 34:0
PI 34:1
PI 34:2
PI 36:0
PI 36:1
PI 36:2
PI 36:3
PI 36:4
PI 38:0
PI 38:1
PI 38:2
PI 38:3
PI 38:4
PI 38:5
PI 38:6
PI 40:4
PI 40:5
PI 40:6
PI 40:7
PI 42:5
PI 42:6
PI 42:7
PG 30:0
PG 32:0
PG 32:1
PG 34:0
PG 34:1
PG 34:2
PG 36:0
PG 36:1
PG 36:2
PG 36:3
PG 36:4
PG 38:0
PG 38:1
PG 38:2
PG 38:3
PG 38:4
PG 38:5
PG 38:6
PG 40:4
PG 40:5
PG 40:6
PG 40:7
BMP 30:0
BMP 32:0
BMP 32:1
BMP 34:0
BMP 34:1
BMP 34:2
BMP 36:0
BMP 36:1
BMP 36:2
BMP 36:3
BMP 36:4
BMP 38:0
BMP 38:1
BMP 38:2
BMP 38:3
BMP 38:4
BMP 38:5
BMP 38:6
BMP 40:4
BMP 40:5
BMP 40:6
BMP 40:7
AcylPG 16:0-34:0
WT
fro
AcylPG 16:0-34:1
AcylPG 16:0-34:2
AcylPG 16:0-36:0
AcylPG 16:0-36:1
AcylPG 16:0-36:2
AcylPG 16:0-36:3
AcylPG 16:0-36:4
AcylPG 16:0-38:1
AcylPG 16:0-38:2
AcylPG 16:0-38:3
AcylPG 16:0-38:4
AcylPG 16:0-38:5
AcylPG 16:0-40:5
AcylPG 16:0-40:6
LPC 16:0
LPC 16:1
LPC 18:0
LPC 18:1
LPC 20:0
LPC 20:1
LPC 20:2
LPC 20:3
LPC 20:4
LPCe 16:0
LPCe 16:1
LPCe 18:0
LPCe 18:1
LPCe 20:0
LPCe 20:1
LPCe 20:2
LPE 16:0
LPE 16:1
LPE 18:0
LPE 18:1
LPE 20:1
LPE 20:2
LPE 20:3
LPE 20:4
LPEp 16:0
LPEp 18:0
LPEp 18:1
LPEp 20:4
LPI 16:0
LPI 16:1
LPI 18:0
LPI 18:1
LPI 20:0
LPI 20:1
LPI 20:3
LPI 20:4
LPS 16:0
LPS 16:1
LPS 18:0
LPS 18:1
LPS 20:0
LPS 20:1
LPS 20:4
LPS 22:0
LPS 22:1
LPS 24:0
LPS 24:1
NAPE 16:0/18:0/20:4
NAPE 18:0/18:1/20:4
NAPE 18:0/20:4/20:4
NAPE 18:0/22:6/20:4
NAPE 18:1/18:1/20:4
NAPE p16:0/18:0/20:4
NAPE p16:0/22:4/20:4
NAPE p18:0/18:1/20:4
NAPE p18:0/20:4/20:4
NAPE p18:0/22:4/20:4
NAPE p18:0/22:6/20:4
NAPE p18:1/18:1/20:4
NAPE p18:1/20:4/20:4
NAPS 16:0-36:0
NAPS 16:0-36:1
NAPS 16:0-36:2
NAPS 16:0-38:0
NAPS 16:0-38:1
NSer 16:0
NSer 16:1
NSer 18:0
NSer 18:1
NSer 20:4
NSer 22:6

FC	Free Cholesterol	PCe	Ether phosphatidylcholine
CE	Cholesterol Ester	PE	Phosphatidylethanolamine
AC	Acyl Carnitine	PEp	Plasmalogen phosphatidylethanolamine
MG	Monoacylglycerol	PS	Phosphatidylserine
DG	Diacylglycerol	PI	Phosphatidylinositol
TG	Triacylglycerol	PG	Phosphatidylglycerol
dhCer	Dihydroceramide	BMP	Bis(monoacylglycero)phosphate
Cer	Ceramide	AcylPG	Acyl Phosphatidylglycerol
SM	Sphingomyelin	LPC	Lysophosphatidylcholine
dhSM	Dihydrosphingomyelin	LPCe	Ether lysophosphatidylcholine
Sulf	Sulfatide	LPE	Lysophosphatidylethanolamine
MHCer	Monohexosylceramide	LPEp	Plasmogen Lysophosphatidylethanolamine
LacCer	Lactosylceramide	LPI	Lysophosphatidylinositol
GM3	Monosialodihexosylganglioside	LPS	Lysophosphatidylserine
GB3	Globotriaosylceramide	NAPE	N-Acyl Phosphatidylethanolamine
PA	Phosphatidic acid	NAPS	N-Acyl Phosphatidylserine
PC	Phosphatylcholine	NSer	N-Acyl Serine

Figure S23 Lipidome heat map of alcohol (EtOH) and fro effects in mice in the ventral striatum (blue- significantly decreased levels, red – significantly increased levels; t-test vs WT-water drinking).

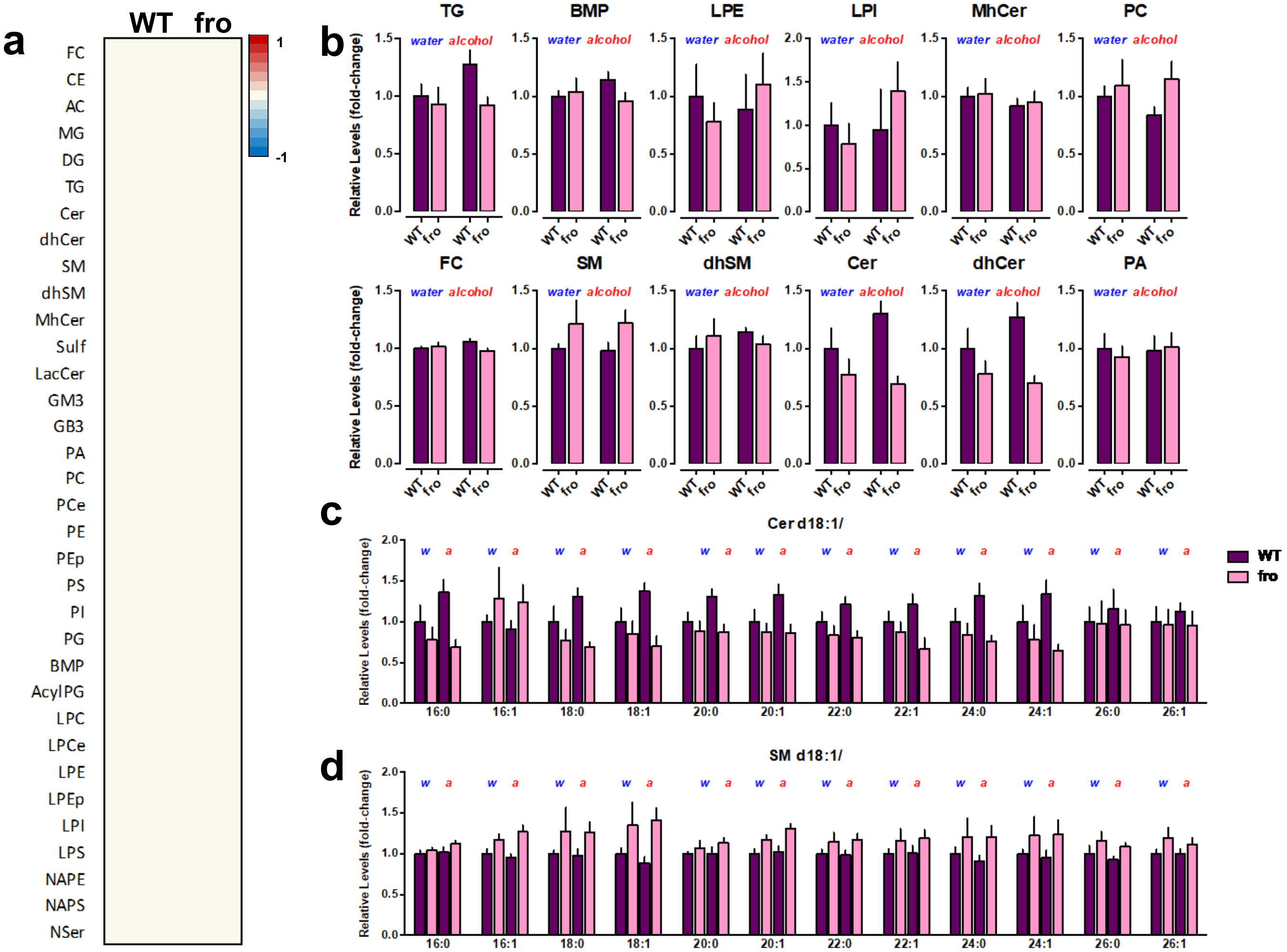

Figure S24 No effects of reduced neurtral sphingomyelinase-2 activity or alcohol consumption on the lipidome in mice.

a

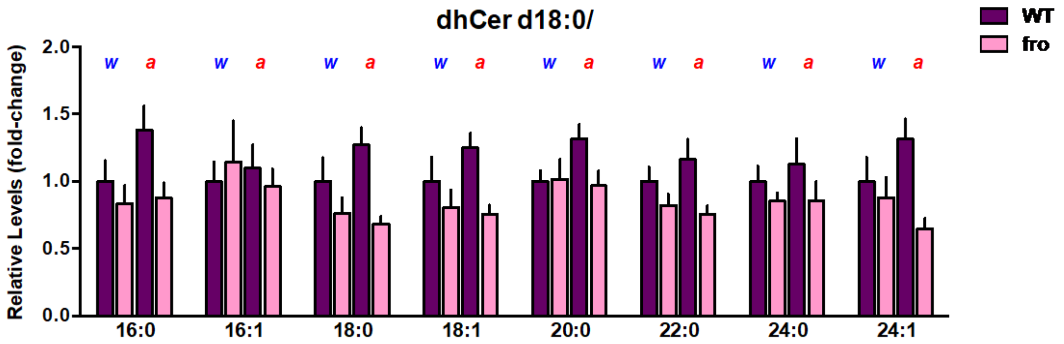

b

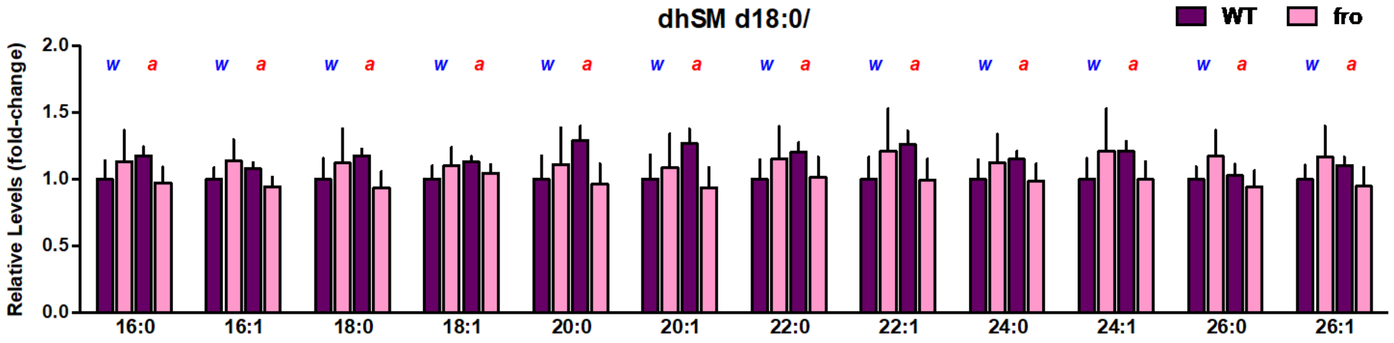

Figure S25 Lipidome target analysis of sphingolipids after alcohol (a) or water (w) drinking in the ventral striatum of mice with reduced neutral sphingomyelinase activity (fro) and wild types. **a**, dihydroceramide species (dhCer) levels, **b**, dihydrosphinghomyeline (dhSM) species levels.

a

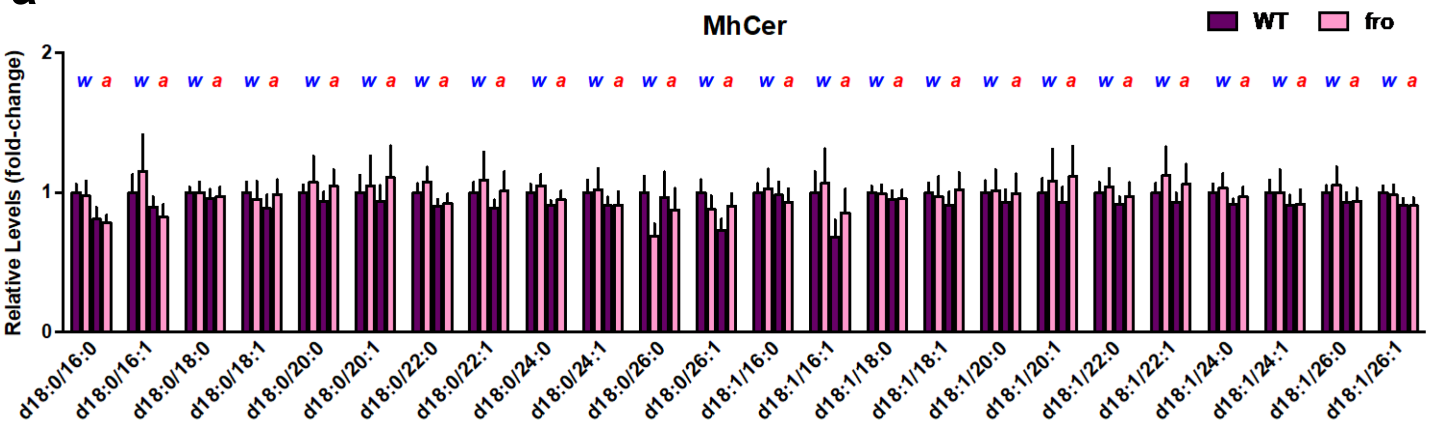

b

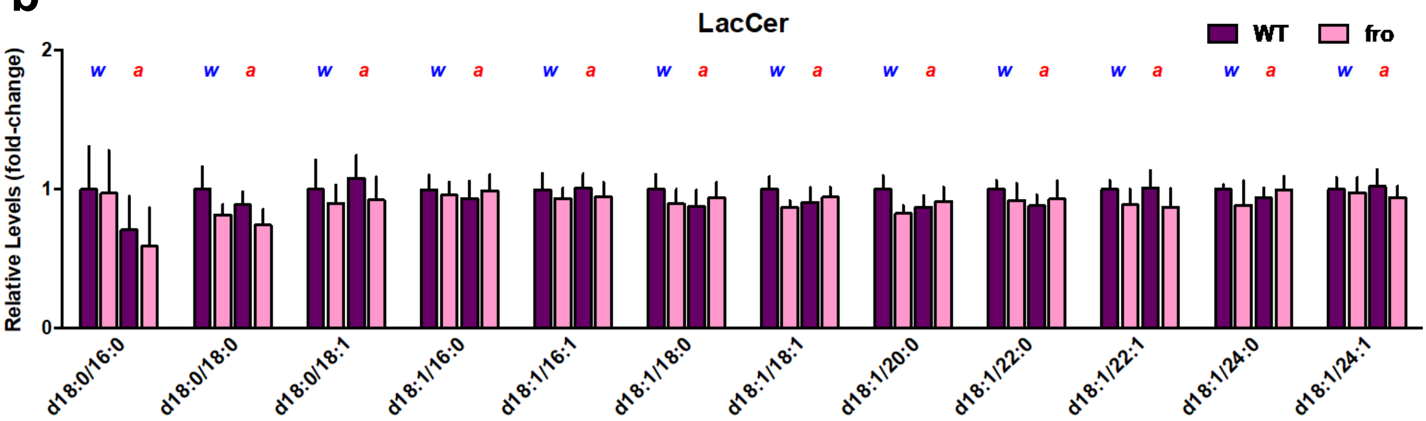

Figure S26 Lipidome target analysis of sphingolipids after alcohol (a) or water (w) drinking in the ventral striatum of mice with reduced neutral sphingomyelinase activity (fro) and wild types. **a**, monohexosylceramide species (MhCer) levels, **b**, lactosylceramide (LacCer) species levels.

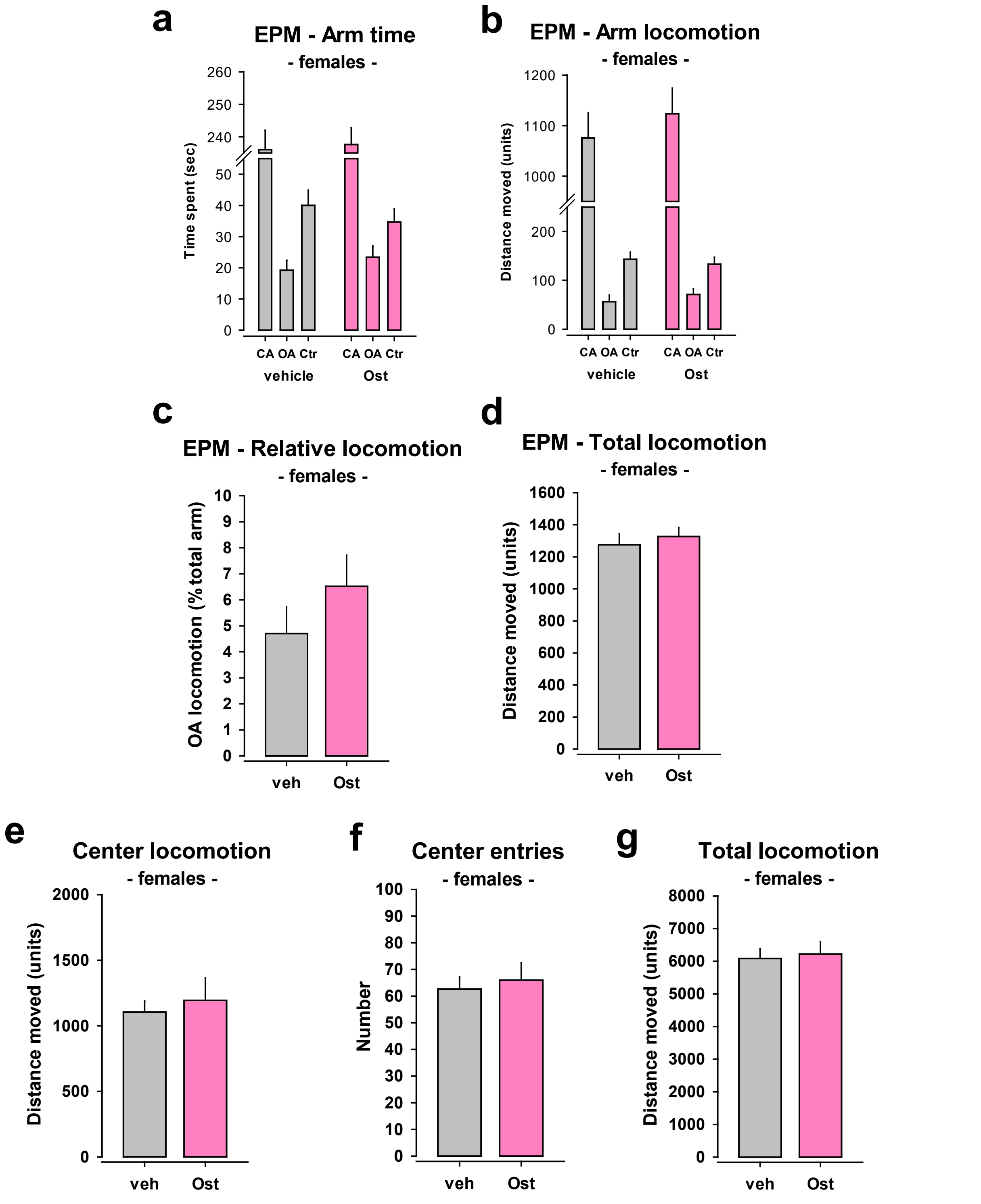

Figure S27 The effects of chronic treatment with an osteocalcin (Ost; i.p.) on anxiety-related behaviour in mice in **a-d**, the elevated plus maze (EPM) and the **e-g**, open field test (OA – open arms, CA – closed arms, Ctr – center).

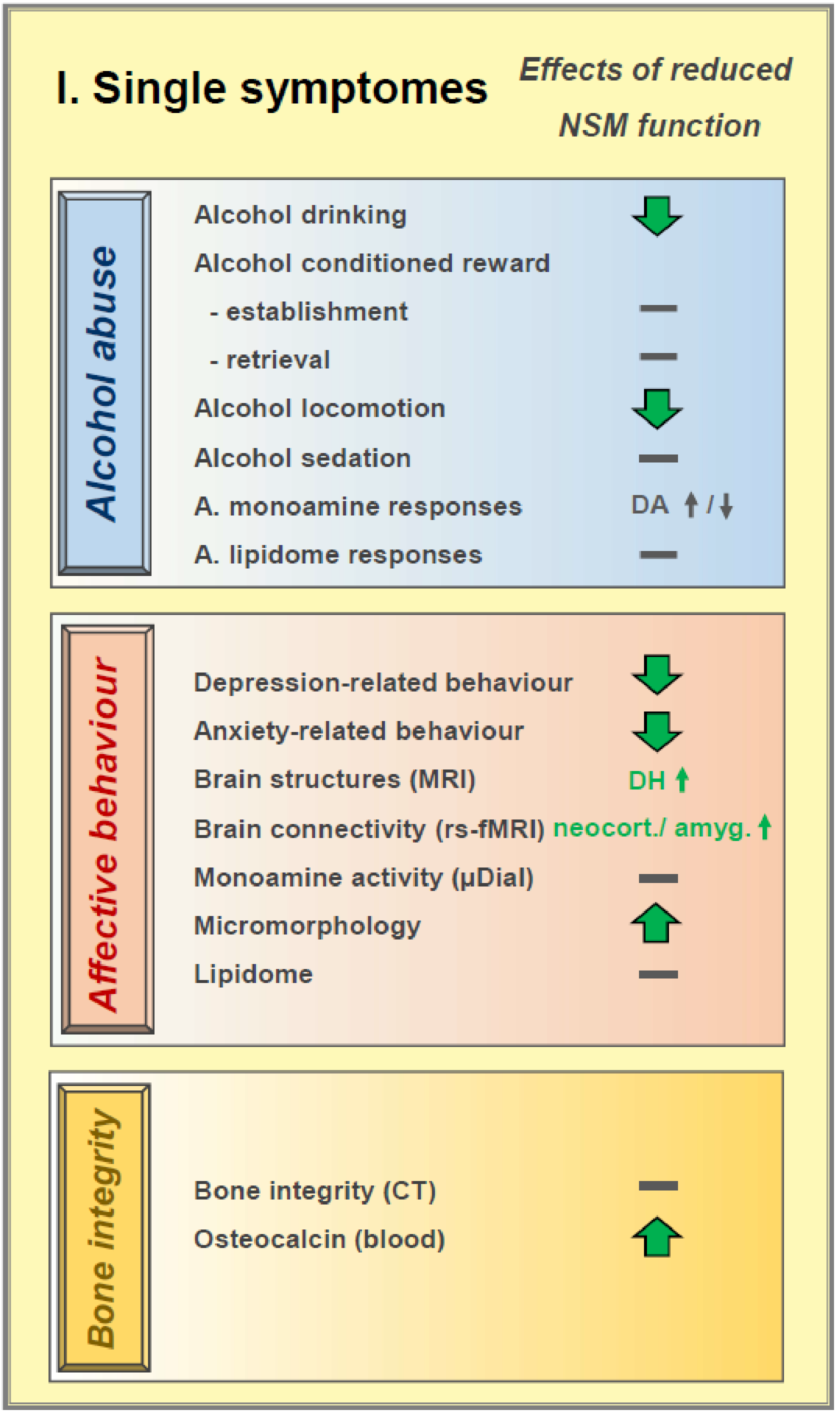

Figure S28 Neutral sphingomyelinase (NSM) mediates the comorbidity trias of alcohol abuse, major depression and bone defects at the level of single syndromes. Main findings of this study. Arrows indicate increase/ decrease of a specific behaviour or brain parameter (green - desirable effect and potential underlying mechanisms; red – undesirable effect and potential underlying mechanisms; grey – no effect and mechanisms with unknown behavioural relevance; A – alcohol, Cereb – cerebellum, DA- dopamine, DH – dorsal hippocampus, dhSM – dihydrosphingomyelin, 5-HT – serotonin, vStr – ventral striatum).

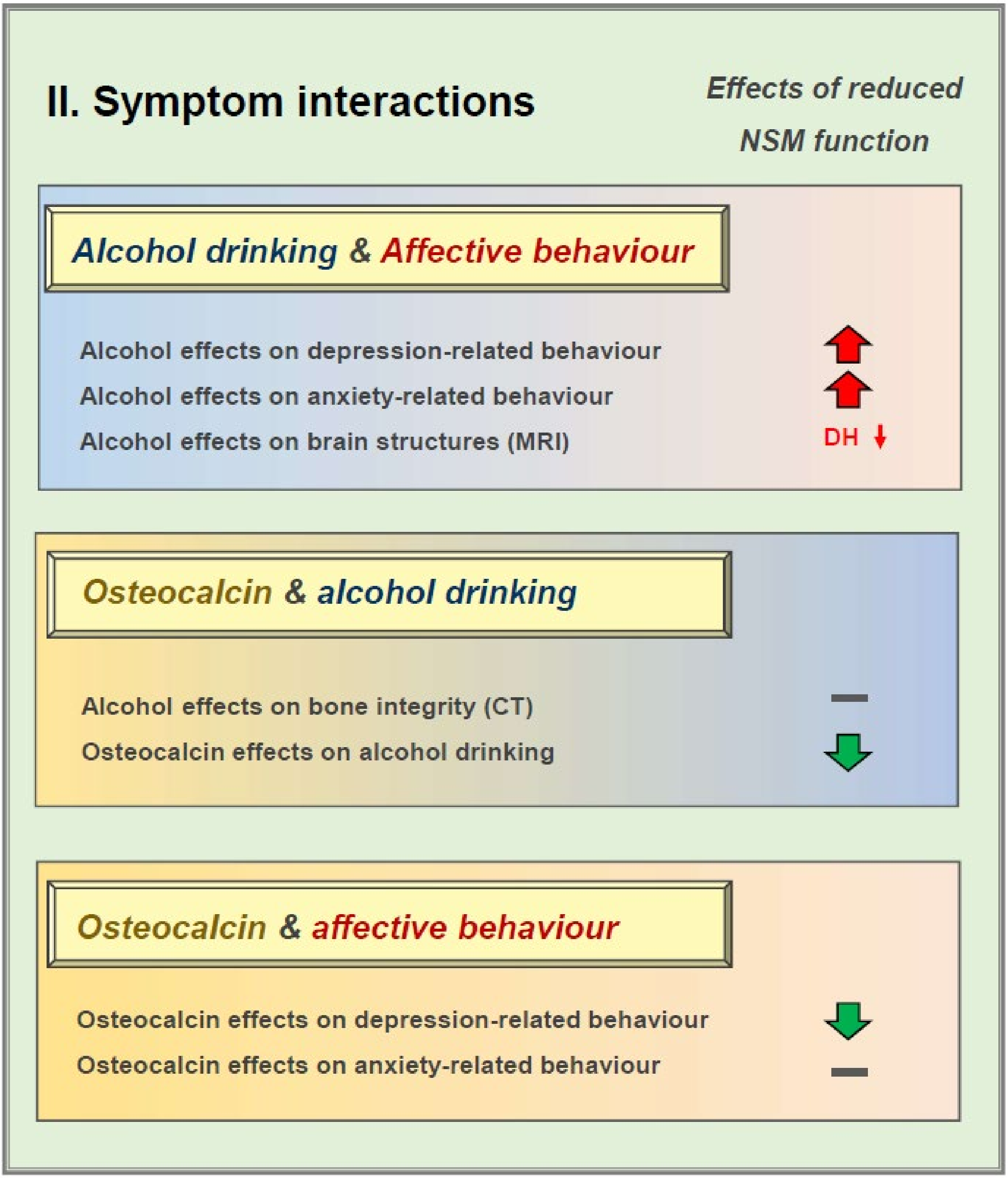

Figure S29 Neutral sphingomyelinase (NSM) mediates the comorbidity trias of alcohol abuse, major depression and bone defects at the level of syndrome interactions. Main findings of this study. Arrows indicate increase/ decrease of a specific behaviour or brain parameter (green - desirable effect and potential underlying mechanisms; red – undesirable effect and potential underlying mechanisms; grey – no effect; A – alcohol, DH – dorsal hippocampus, O – osteocalcin).
