# Supplementary material for: Neutral sphingomyelinase mediates the co-morbidity trias of alcohol abuse, major depression and bone defects
Source: Mol Psychiatry. 2021 Sep 28;26(12):7403–16. doi: 10.1038/s41380-021-01304-w (PMC8872992; doi:10.1038/s41380-021-01304-w)
Supplement: Supplementary file 2 — Supplementary tables [file 41380_2021_1304_MOESM2_ESM.pdf]

Table S1: Descriptive data for the study participants

	N	Mean	SD	Min	Max
Genotype data	456693				
Behavioural data					
Alcohol Drink Frequency	456693	2.95	1.522	1	6
Nervous Feelings	456693	0.236	0.423	0	1
Worrier/Anxious Feelings	456693	0.565	0.497	0	1
Alcohol Drinker Status	456693	1.874	0.437	0	2
Frequency of Depressed Mood in the last 2 weeks	456693	1.308	0.624	1	4
Bone Density data					
Right Femur Bone Mineral Density	4515	0.7467	0.157	0	1.476
Left Femur Bone Mineral Density	4515	0.7396	0.157	0	1.408
Neuroimaging data					
T1 grey matter					
Insula	4515	12589	1309.9	7645	18600
Hippocampus	4515	8596	817.5	5700	11870

Table S2: List of 26 haplotype within SMPD3 with MHF > 0.01.

	rs117949548_TC	rs71395853_TC	rs79978301_GA	rs117668880_AG	rs55847153_TC	rs11646700_GA	rs8051517_CT	rs9940558_TA	rs12444999_TC	rs1111232_CT	rs1364344_CT	rs1125848_CT	rs80343315_AG	rs16957943_TC	Frequency
Hap1	C	C	A	G	C	G	T	T	C	C	C	C	G	C	0.323763
Hap2	C	C	A	G	C	A	T	A	C	C	C	C	G	C	0.322046
Hap3	C	C	A	G	C	G	T	A	C	C	C	C	G	C	0.246824
Hap4	C	C	A	G	C	A	T	A	C	T	T	T	G	C	0.189457
Hap5	C	C	G	G	C	G	T	T	C	C	C	C	G	C	0.181035
Hap6	C	C	A	G	C	A	T	A	C	C	T	T	G	C	0.053996
Hap7	C	T	A	G	C	A	C	A	C	C	C	C	G	C	0.051039
Hap8	C	C	A	G	C	A	T	A	C	C	T	C	G	C	0.048444
Hap9	C	C	A	G	T	A	T	A	C	C	C	C	G	C	0.046985
Hap10	T	C	A	G	C	A	T	A	C	T	C	C	G	C	0.04297
Hap11	C	C	A	A	C	A	T	A	C	T	T	T	G	C	0.037925
Hap12	C	C	A	G	C	G	T	T	T	C	C	C	G	C	0.037026
Hap13	C	C	A	G	C	A	T	A	C	T	C	C	G	C	0.036998
Hap14	C	C	A	G	C	A	T	A	C	T	C	T	G	C	0.036643
Hap15	C	C	A	G	C	G	T	T	C	C	C	C	G	T	0.032435
Hap16	C	C	A	G	C	G	T	A	C	T	C	C	G	C	0.027291
Hap17	C	T	A	G	C	A	C	A	C	T	C	C	G	C	0.02349
Hap18	C	C	A	G	C	A	T	A	C	C	C	T	G	C	0.023313
Hap19	C	C	A	G	C	G	C	A	C	C	C	C	G	C	0.019138
Hap20	C	C	A	G	C	G	T	T	C	C	C	C	A	C	0.018689
Hap21	C	C	A	G	C	A	T	A	C	T	T	C	G	C	0.0163
Hap22	C	C	A	G	C	G	T	T	C	C	T	T	G	C	0.015851
Hap23	C	C	A	G	C	G	C	T	C	C	C	C	G	C	0.013018
Hap24	C	C	A	G	C	G	T	T	C	T	C	C	G	C	0.012519
Hap25	C	C	A	A	C	A	T	A	C	T	C	C	G	C	0.011625
Hap26	C	C	A	G	C	G	T	T	C	C	C	T	G	C	0.010564

Table S3: Hotelling’s t-test between Haplotype phases and behavioural phenotypes. P-values are significant at level 0.05 *, 0.01**, 0.001***.

	Combined behavioural outcomes	Alcohol Intake Frequency	Nervous Feelings	Worrier/Anxious Feelings	Alcohol Drinker Status	Frequency of Depressed Mood
Hap1	F=15.3; p=1.567×10^{-14}***	F=34.926; p=3.427×10^{-9}***	p=0.4974	p=0.4937	F=63.89; p=1.317×10^{-15}***	F=7.376; p=0.00661**
Hap2	p=0.58832	p=0.1253	p=0.9984	p=0.6978	p=0.1312	p=0.4100
Hap3	F=24.6; p=4.096×10^{-24}***	F=100.19; p=2.17×10^{-29}***	p=0.1003	F=11.694; p=0.000627***	F=60.55; p=7.203×10^{-15}***	p=0.7528
Hap4	F=5.0; p =2.663×10^{-4}***	F=17.87; p=2.371×10^{-5}***	p=0.5611	p=0.9964	F=18.85; p=1.414×10^{-5}***	p=0.2104
Hap5	F=32.0; p =6.157×10^{-32}***	F=128.2; p=2.88×10^{-36}***	p=0.4631	p=0.057	F=72.57; p=6.53×10^{-23}***	F=17.38; p=3.058×10^{-5}***
Hap6	F=17.2; p =1.963×10^{-16}***	F=55.74; p=8.279×10^{-14}***	F=5.383; p=0.0203*	F=9.004; p=0.00269**	F=39.42; p=3.413×10^{-10}***	F=6.317; p=0.0120*
Hap7	F=3.1; p =0.0110*	F=4.070; p=0.0437	p=0.1234	p=0.3543	F=5.37; p=0.0204*	F=4.5693; p=0.0326*
Hap8	F=36.2; p=2.964×10^{-36}***	F=140.1; p=7.72×10^{-41}***	p=0.0844	p=0.1569	F=101.74; p=4.60×10^{-33}***	F=13.28; p=0.0002685***
Hap9	F=8.1; p=3.133×10^{-7}***	F=26.57; p=2.54×10^{-7}***	F=4.154; p=0.04154*	F=4.953; p=0.0260*	F=22.95; p=1.665×10^{-6}***	p=0.9857
Hap10	F=13.5; p=1.219×10^{-12}***	F=51.43; p=7.445×10^{-13}***	p=0.4151	p=0.6205	F=35.82; p=2.16×10^{-9}***	F=9.988; p=0.001575**
Hap11	F=10.2; p=2.359×10^{-9}***	F=20.48; p=6.02×10^{-6}***	p=0.1972	p=0.8324	F=43.24; p=4.84×10^{-11}***	p=0.0808
Hap12	F=46.3; p=7.275×10^{-47}***	F=168.55; p= 1.36×10^{-51}***	p=0.1826	p=0.2448	F=175.59; p=3.37×10^{-59}***	F=5.938; p=0.0148*
Hap13	p=0.4391	p=0.4339	p=0.1190	p=0.3344	p=0.2916	p=0.1940
Hap14	F=3.7; p =0.00395**	F=8.4095; p=0.00444**	p=0.1856	F=5.8921; p=0.01521*	F=10.20; p=0.001404**	p=0.6021
Hap15	F=3.6; p=0.00444**	F=9.724; p=0.001819**	F=6.167; p=0.01302*	p=0.11223	F=7.898; p=0.00495**	p=0.09850
Hap16	F=5.0; p=2.663×10^{-4}***	F=15.32; p=9.091×10^{-5}***	F=4.039; p=0.04445*	p=0.8194	F=11.0838; p=0.0008709***	p=0.2207
Hap17	p=0.89544	p=0.391	p=0.7211	p=0.4326	p=0.9490	p=0.9283
Hap18	p=0.28586	p=0.8308	p=0.1735	p=0.3768	p=0.3030	p=0.8959
Hap19	F=5.2; p=1.944×10^{-4}***	F=18.47; p=1.727×10^{-5}***	p=0.4144	p=0.1234	F=14.11; p=0.000172***	p=0.5145
Hap20	p=0.081301	p=0.2569	F=4.984; p=0.02559*	p=0.2115	p=0.2006	p=0.3440
Hap21	F=69.4; p=2.283×10^{-71}***	F=183.45; p=7.37×10^{-55}***	p=0.2613	F=4.450; p=0.0349*	F=315.58; p=1.08×10^{-92}***	p=0.2099
Hap22	p=0.26642	F=4.874; p=0.0273*	p=0.3403	p=0.2270	p=0.1814	p=0.6923
Hap23	F=4.9; p=3.185×10^{-4}***	F=19.36; p=1.080×10^{-5}***	p=0.9430	p=0.4608	F=13.06; p=0.0003021***	p=0.1343
Hap24	p=0.10100	p=0.8170	F=7.3551; p=0.006688**	p=0.1602	p=0.4619	p=0.1412
Hap25	F=4.5; p=7.025×10^{-4}***	F=20.09; p=7.394×10^{-6}***	p=0.7720	p=0.9488	F=11.62; p=0.0006512***	p=0.2301
Hap26	p=0.36528	p=0.1502	p=0.0975	p=0.9199	p=0.7987	p=0.5087

Table S4: Univariate association between Haplotype phases and bone mineral density. P-values are significant at level 0.05 *, 0.01**, 0.001***.

	Correlation with Right Femur Bone Mineral Density	Correlation with Left Femur Bone Mineral Density
**Hap1**	p=0.793731	p=0.796045
**Hap2**	p=0.831496	p=0.443547
**Hap3**	p=0.323869	p=0.292664
**Hap4**	p=0.924677	p=0.88726
**Hap5**	p=0.81418	p=0.65231
**Hap6**	p=0.133779	p=0.108344
**Hap7**	p=0.934147	p=0.777612
**Hap8**	p=0.333305	p=0.646753
**Hap9**	p=0.417121	p=0.424202
**Hap10**	p=0.630329	p=0.521556
**Hap11**	p=0.389839	p=0.322977
**Hap12**	p=0.241528	p=0.314492
**Hap13**	p=0.272385	p=0.594835
**Hap14**	p=0.326566	p=0.314511
**Hap15**	p=0.577672	p=0.481141
**Hap16**	p=0.052785	p=0.241304
**Hap17**	p=0.548409	p=0.792932
**Hap18**	p=0.523122	p=0.462524
**Hap19**	p=0.774004	p=0.666006
**Hap20**	p=0.720126	p=0.606242
**Hap21**	p=0.332789	p=0.562485
**Hap22**	p=0.932951	p=0.577543
**Hap23**	p=0.231819	p=0.183841
**Hap24**	p=0.131046	p=0.086701
**Hap25**	p=0.248203	p=0.431569
**Hap26**	t=3.109, p=0.001888, p-FDR=0.04909*	t=3.329, p=0.000878, p-FDR=0.04563*
